# Supplementary material for: Xyloglucan processing machinery in Xanthomonas pathogens and its role in the transcriptional activation of virulence factors
Source: Nat Commun. 2021 Jun 30;12:4049. doi: 10.1038/s41467-021-24277-4 (PMC8245568; doi:10.1038/s41467-021-24277-4)
Supplement: Supplementary file 1 — Supplementary Information [file 41467_2021_24277_MOESM1_ESM.pdf]

SUPPLEMENTARY INFORMATION

Xyloglucan processing machinery in *Xanthomonas* pathogens and its role in the transcriptional activation of virulence factors

Plinio S. Vieira[1#], Isabela M. Bonfim[1,2#], Evandro A. Araujo[1,3], Ricardo R. Melo[1], Augusto R. Lima[1], Melissa R. Fessel[4], Douglas A. A. Paixão[1], Gabriela F. Persinoti[1], Silvana A. Rocco[5], Tatiani B. Lima[1], Renan A. S. Pirolla[1], Mariana A. B. Morais[1], Jessica B. L. Correa[1], Leticia M. Zanphorlin[1], Jose A. Diogo[1,2], Evandro A. Lima[1], Adriana Grandis[6], Marcos S. Buckeridge[6], Fabio C. Gozzo[7], Celso E. Benedetti[5], Igor Polikarpov[8], Priscila O. Giuseppe[1*] and Mario T. Murakami[1*]

[1]Brazilian Biorenewables National Laboratory (LNBR), Brazilian Center for Research in Energy and Materials (CNPEM), Campinas, São Paulo, Brazil.

[2]Graduate Program in Functional and Molecular Biology, Institute of Biology, University of Campinas, Campinas, São Paulo, Brazil.

[3]Brazilian Synchrotron Light Laboratory (LNLS), Brazilian Center for Research in Energy and Materials (CNPEM), Campinas, São Paulo, Brazil.

[4]Butantan Institute, Butantan Foundation, São Paulo, São Paulo, Brazil.

[5]Brazilian Biosciences National Laboratory (LNBio), Brazilian Center for Research in Energy and Materials (CNPEM), Campinas, São Paulo, Brazil.

[6]Department of Botany, Institute of Biosciences, University of São Paulo, São Paulo, Brazil.

[7]Institute of Chemistry, University of Campinas, Campinas, São Paulo, Brazil.

[8]São Carlos Institute of Physics, University of São Paulo, São Carlos, São Paulo, Brazil.

[#]These authors have equally contributed to this work

[*]Correspondence and requests should be addressed to M.T.M (mario.murakami@lnbr.cnpem.br) or P.O.G (priscila.giuseppe@lnbr.cnpem.br)

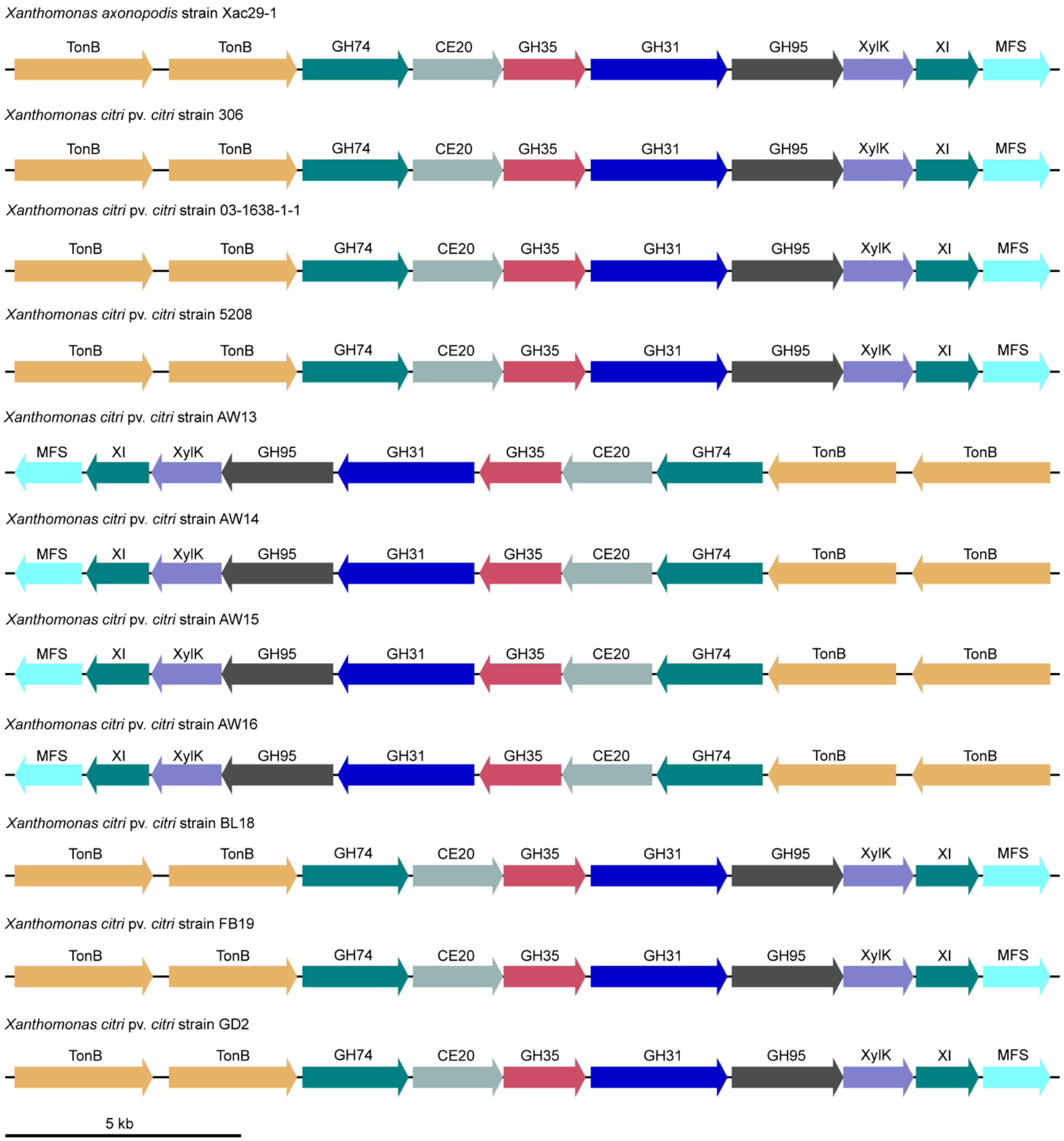

Supplementary Figure 1. XyGUL genes are conserved among *Xanthomonas citri* strains. Conservation of XyGUL genes and downstream genes corresponding to xylose metabolism and MFS transporter was evaluated in all complete genomes of *X. citri* pv. *citri* currently available at the Refseq database. Each gene was colored according to the family it belongs to or its functional annotation.

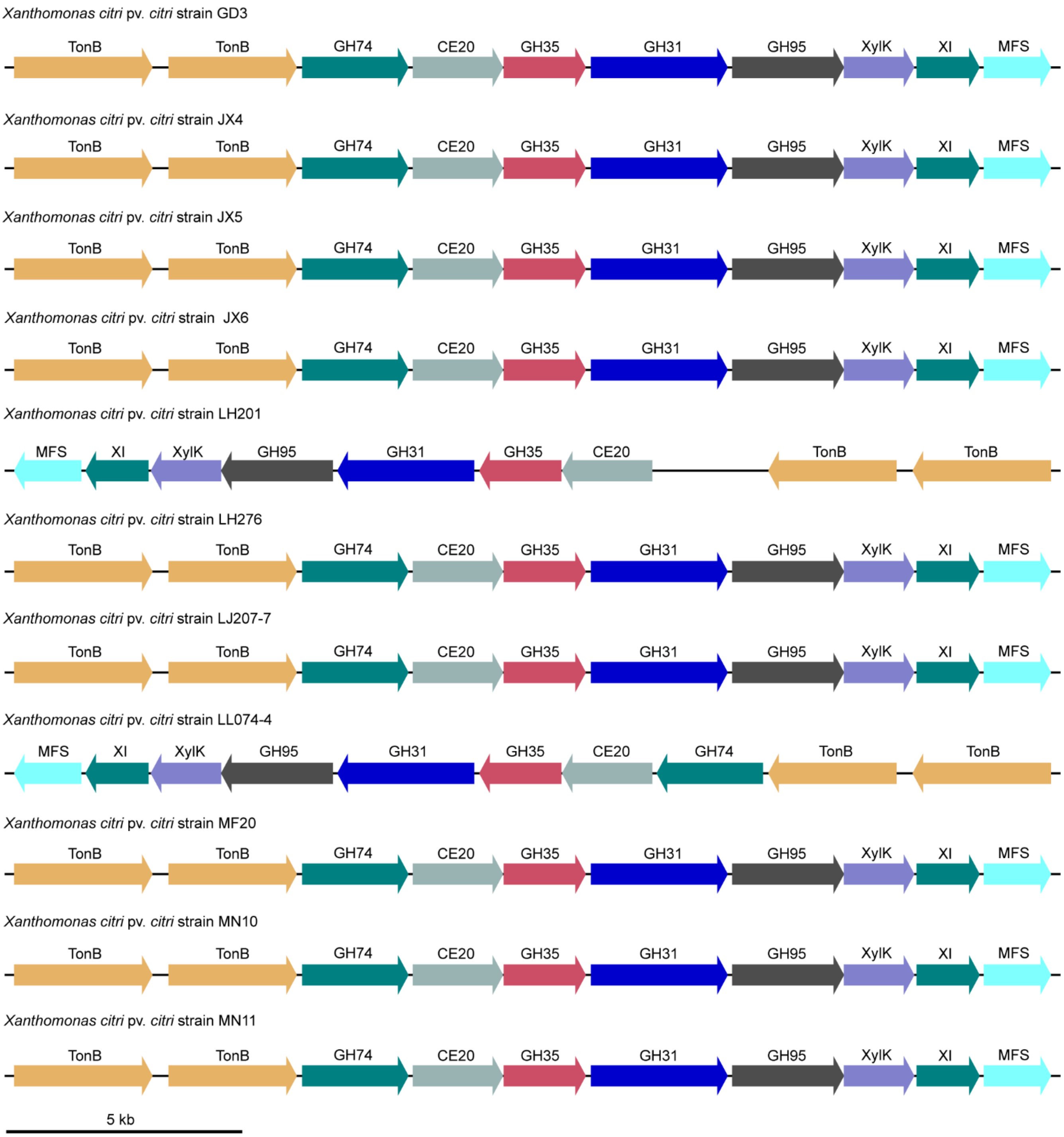

Supplementary Figure 1. (Continued) XyGUL genes are conserved among *Xanthomonas citri* strains. Conservation of XyGUL genes and downstream genes corresponding to xylose metabolism and MFS transporter was evaluated in all complete genomes of *X. citri* pv. *citri* currently available at the Refseq database. Each gene was colored according to the family it belongs to or its functional annotation.

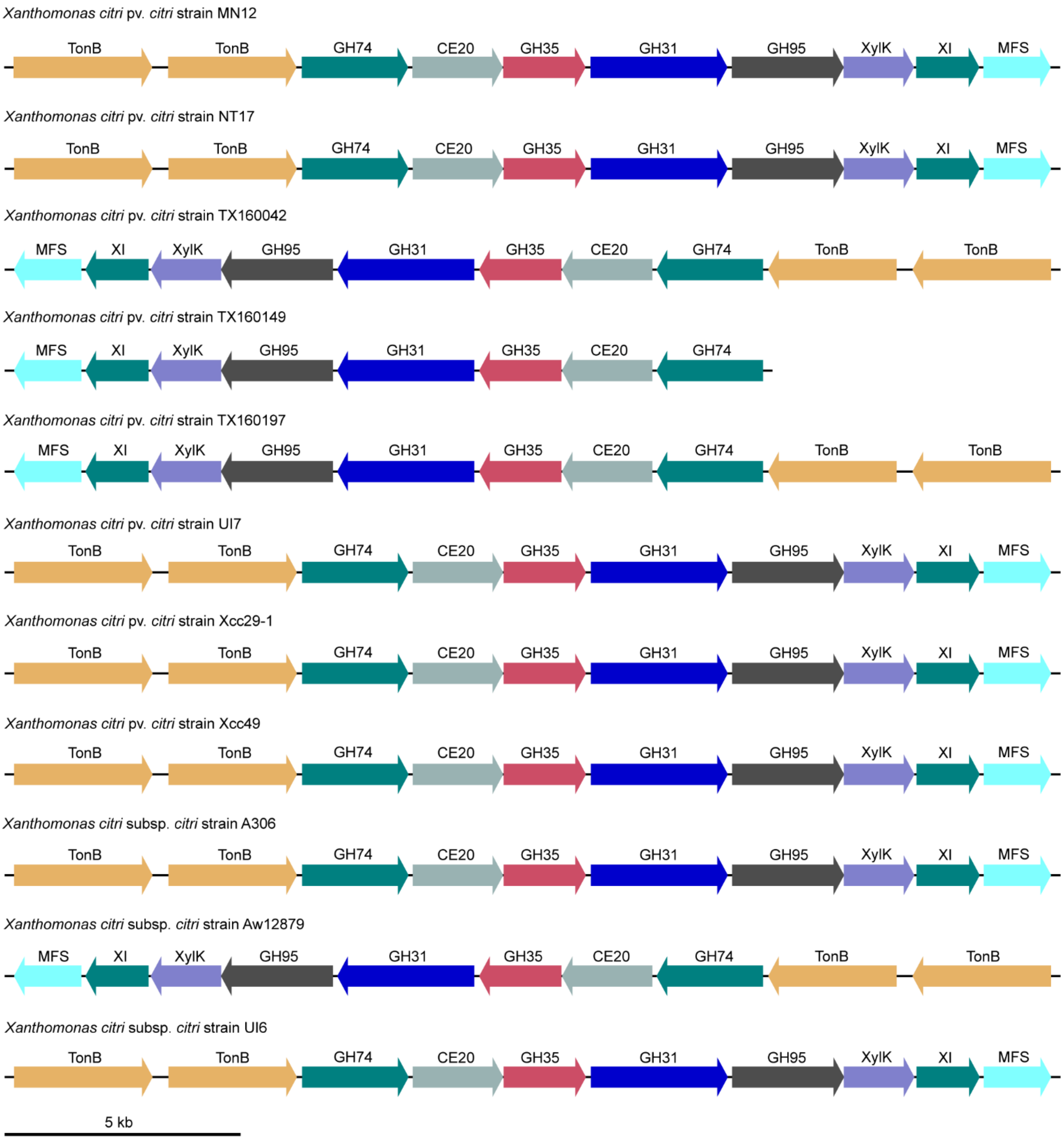

Supplementary Figure 1. (Continued) XyGUL genes are conserved among *Xanthomonas citri* strains. Conservation of XyGUL genes and downstream genes corresponding to xylose metabolism and MFS transporter was evaluated in all complete genomes of *X. citri* pv. *citri* currently available at the Refseq database. Each gene was colored according to the family it belongs to or its functional annotation.

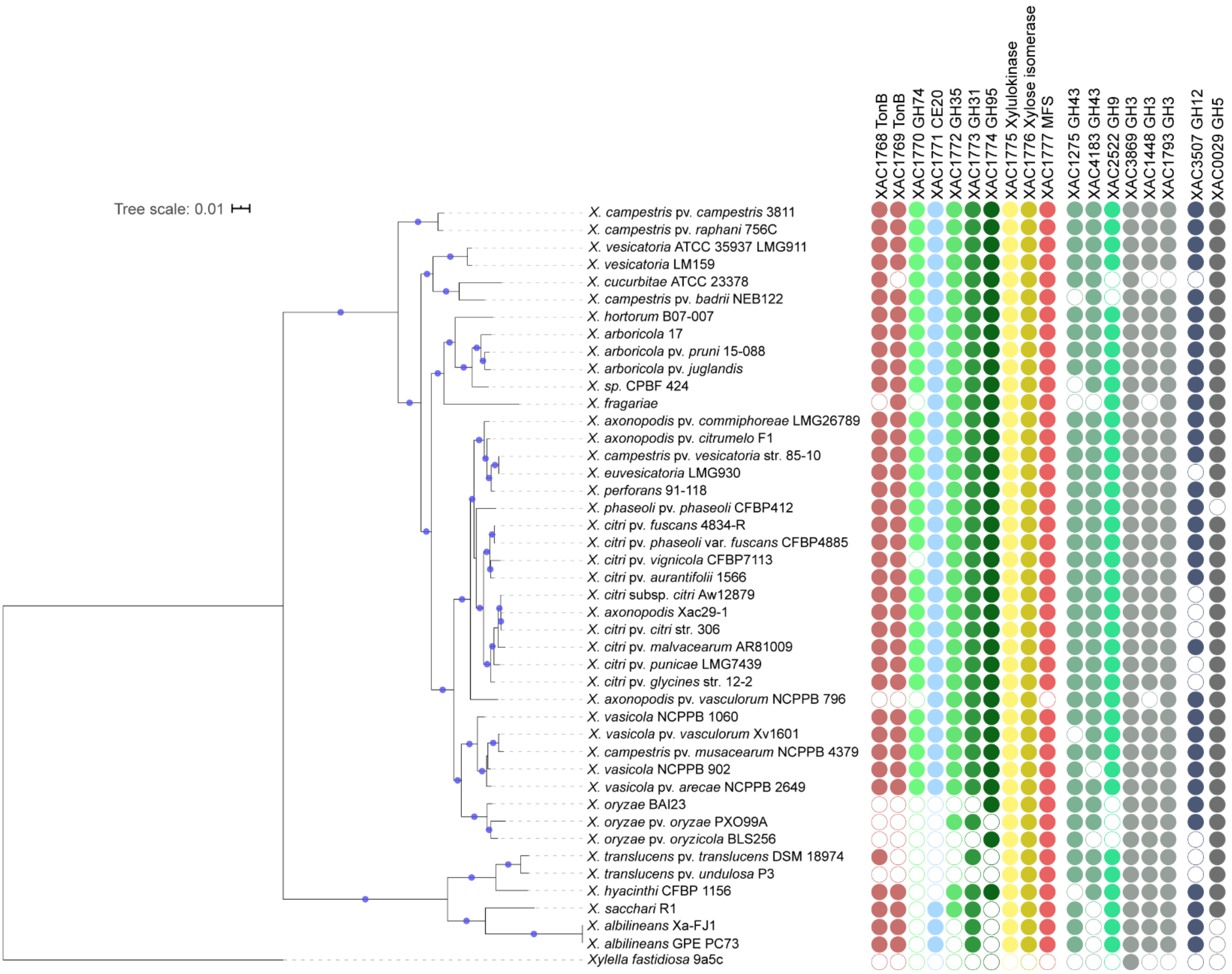

Supplementary Figure 2. XyGUL conservation in *Xanthomonas* species. Maximum likelihood phylogenetic reconstruction of *Xanthomonas* species, as depicted in Fig. 1, showing the presence (filled circles) or absence (open circles) of XyGUL genes (from XAC1768 to XAC1774), downstream genes corresponding to xylose metabolism (XAC1775 and XAC1776) and MFS transporter (XAC1777), and genes encoding for accessory enzymes (from XAC1275 to XAC0029, details in Supplementary Table 3). Each gene is color-coded according to the family it belongs to or its functional annotation. Nodes with bootstrap support values > 80 are shown with a blue circle. *Xylella fastidiosa* 9a5c was used as outgroup.

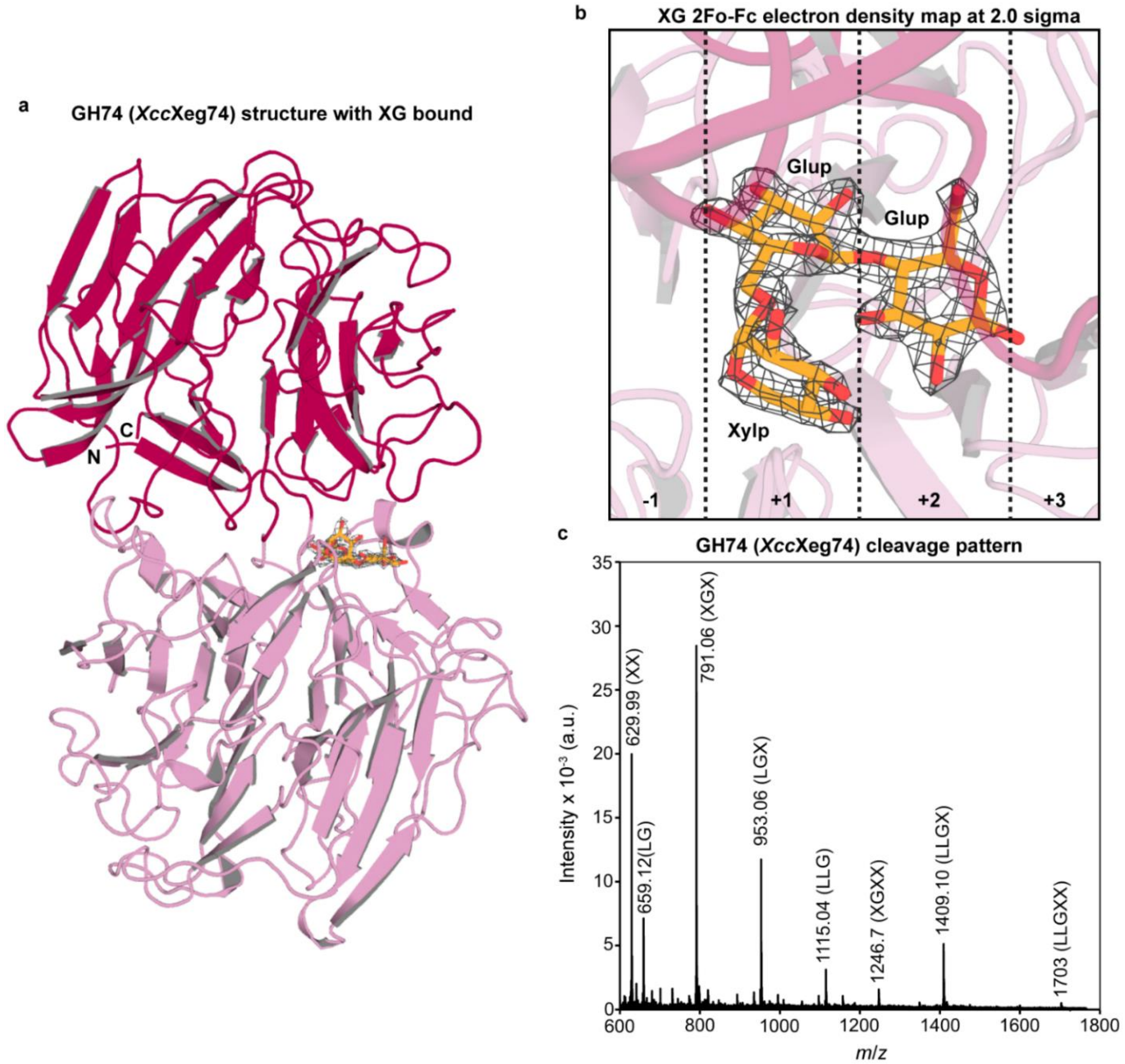

Supplementary Figure 3. Structure of *Xanthomonas* GH74 (*Xcc*Xeg74) in complex with XG and mass spectrometry spectrum of cleavage products. (a) Cartoon representation of the crystal structure of *Xcc*Xeg74 monomer with XG oligosaccharide (sticks, yellow C-atoms) bound to the positive subsite region. The two seven-bladed β-propellers are shown in shades of pink. XG = α-D-xylopyranosyl-(1->6)-β-D-glucopyranosyl-(1->4)-β-D-glucopyranose. (b) Structural details of the active site including the XG oligosaccharide, and the $2F_O$-F_C electron density map contoured at 2σ level. Numbers -1, +1, +2, +3 indicate subsites. Glup = β-D-glucopyranosyl moiety. Xylp= α-D-xylopyranosyl moiety. (c) MALDI-TOF spectrum of the GH74 released products from *Copaifera langsdorfii* xyloglucan. Numbers above the peaks represent *m/z* values of each product (see Fig. 2). Letters indicate the type of substitutions appended to the glucose backbone of the identified oligosaccharides. G = non-substituted glucose; X = glucose substituted with a xylose at C-6; L = X with a galactose appended at xylose C-2.

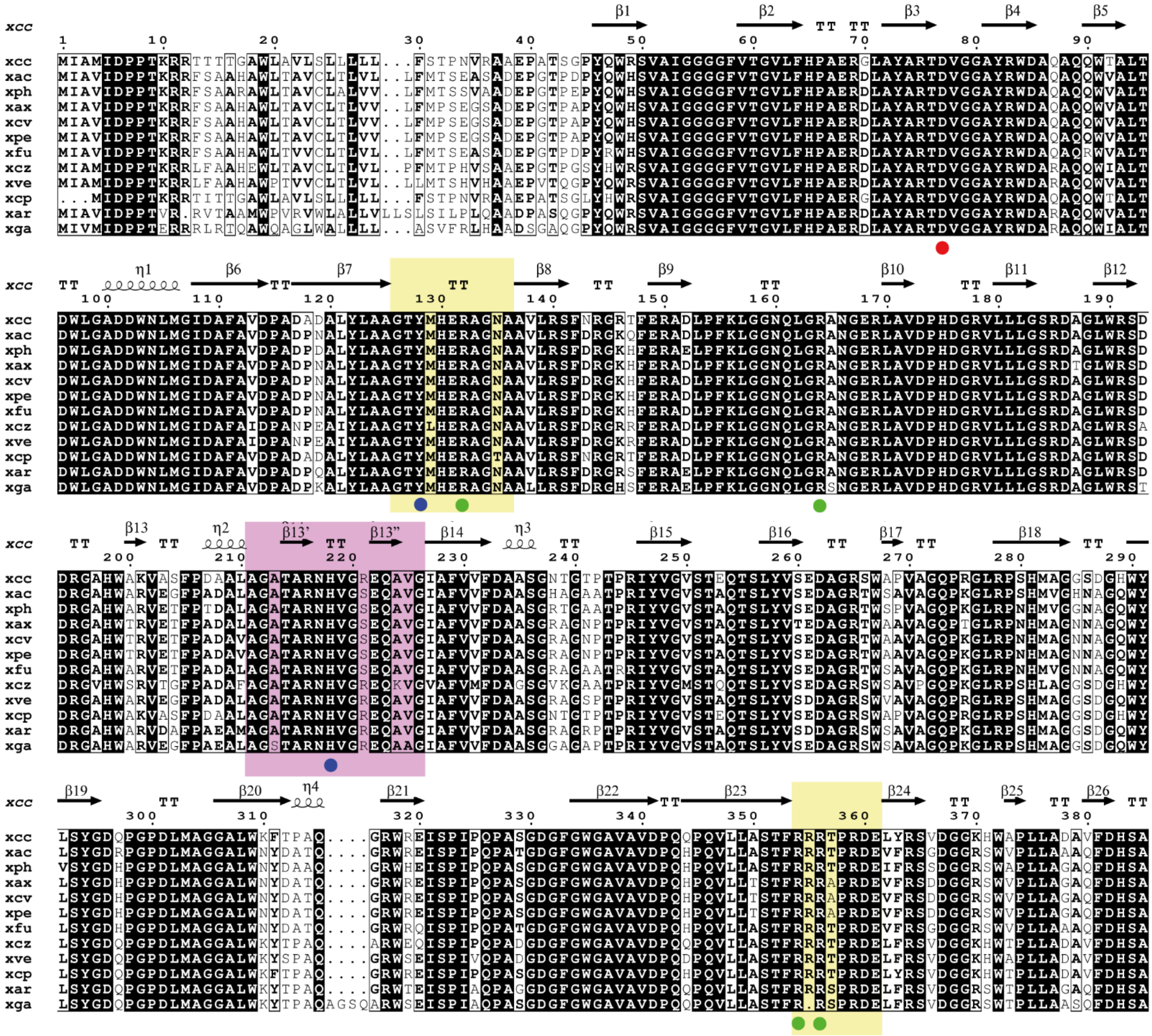

Supplementary Figure 4. Alignment of GH74 sequences from representative *Xanthomonas* species. Numbers above the alignment represent the amino acid residues numbers of *Xcc*Xeg74 in the sequence alignment. Secondary structure elements are also depicted above and labeled according to the crystallographic model of *Xcc*Xeg74. Arrows = β-sheets, helices = α-helices and T = β-turn. Yellow boxes delimit the loop extensions observed in *Xanthomonas* enzymes. The light purple box indicates the β-hairpin insertion conserved across *Xanthomonas* sequences. Green circles mark the arginine residues involved in substrate binding, blue circles indicate the residues that make stacking interactions with the substrate, and red circles identify the catalytic residues D77 (acid) and D466 (base) (See Fig. 2). Residues in black boxes are fully conserved. The representative species are *xcc*: *Xanthomonas campestris* pv. *campestris* ATCC 33913, *xac*: *Xanthomonas citri* pv. *citri* 306, *xph*: *Xanthomonas phaseoli* pv. *phaseoli* CFBP6546R, *xax*, *Xanthomonas axonopodis* pv. *citrumelo* F1, *xcv*, *Xanthomonas campestris* pv. *vesicatoria* 85-10, *xpe*, *Xanthomonas perforans* LH3, *xfu*, *Xanthomonas citri* pv. *fuscans* 4834-R, *xcz*, *Xanthomonas cucurbitae* ATCC 23378, *xve*, *Xanthomonas vesicatoria* ATCC 35937 LMG911, *xcp*, *Xanthomonas campestris* pv. *raphani* 756C, *xar*, *Xanthomonas arboricola* 17, *xga*, *Xanthomonas gardneri* ICMP 7383. Alignment performed with the Clustal Ω[1] server and image produced with ESPript 3.0 server[2].

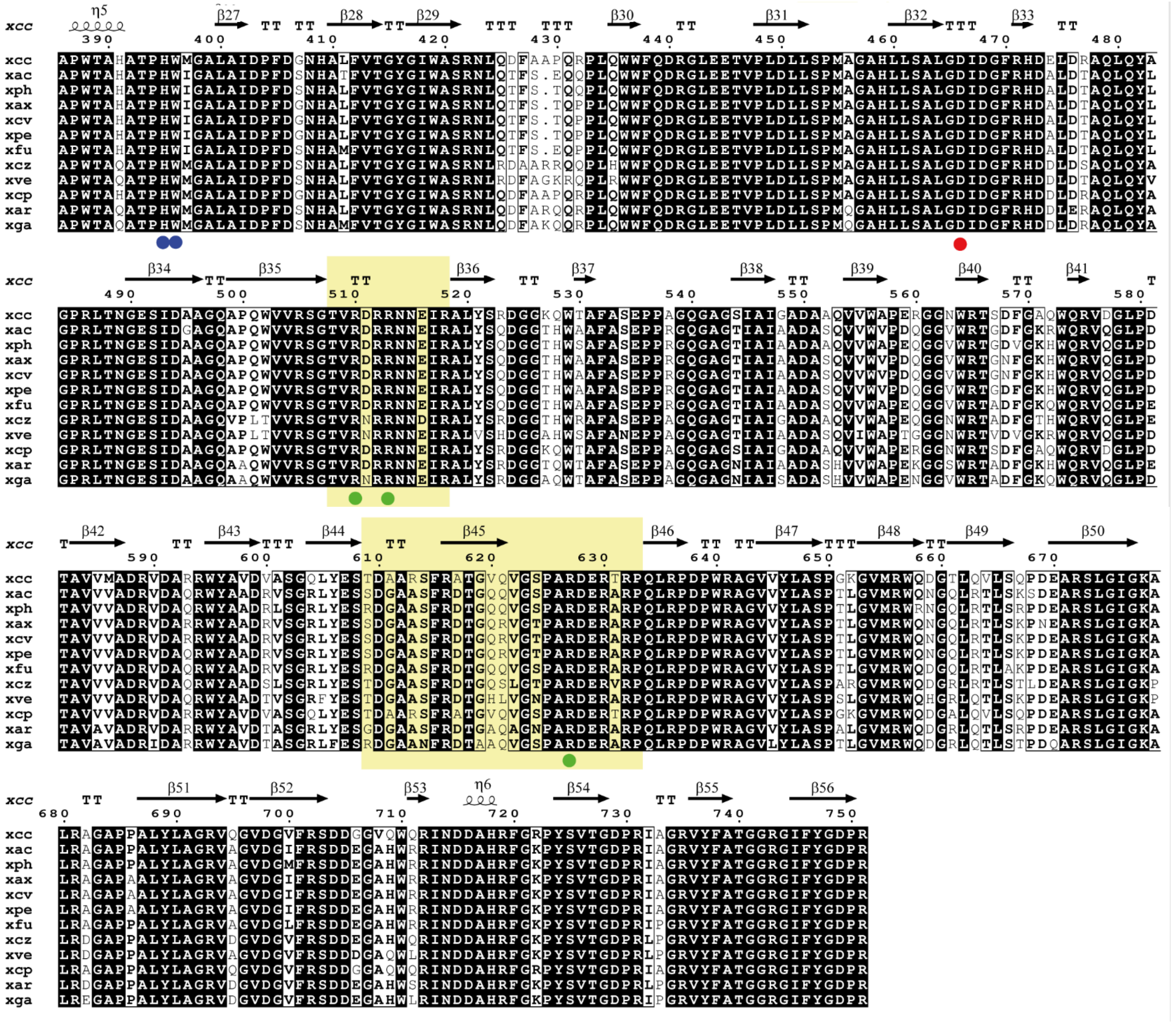

Supplementary Figure 4. Alignment of GH74 sequences from representative *Xanthomonas* species (Continued). Numbers above the alignment represent the amino acid residues numbers of *Xcc*Xeg74 in the sequence alignment. Secondary structure elements are also depicted above and labeled according to the crystallographic model of *Xcc*Xeg74. Arrows = β-sheets, helices = α-helices and T = β-turn. Yellow boxes delimit the loops extensions observed for *Xanthomonas* enzymes. The light purple box marks the β-hairpin insertion, also conserved in other *Xanthomonas*. Below the alignment, green circles mark the arginine residues involved in substrate binding, blue circles mark the residues that make stacking interactions with the substrate, and red circles identify the catalytic residues D77 (acid) and D466 (base) (See Fig. 2). Residues in black boxes are fully conserved. The representative species are *xcc*: *Xanthomonas campestris* pv. *campestris* ATCC 33913, *xac*: *Xanthomonas citri* pv. *citri* 306, *xph*: *Xanthomonas phaseoli* pv. *phaseoli* CFBP6546R, *xax*, *Xanthomonas axonopodis* pv. *citrumelo* F1, *xcv*, *Xanthomonas campestris* pv. *vesicatoria* 85-10, *xpe*, *Xanthomonas perforans* LH3, *xfu*, *Xanthomonas citri* pv. *fuscans* 4834-R, *xcz*, *Xanthomonas cucurbitae* ATCC 23378, *xve*, *Xanthomonas vesicatoria* ATCC 35937 LMG911, *xcp*, *Xanthomonas campestris* pv. *raphani* 756C, *xar*, *Xanthomonas arboricola* 17, *xga*, *Xanthomonas gardneri* ICMP 7383. Alignment performed with the Clustal Ω[1] server and image produced by ESPript 3.0 server[2].

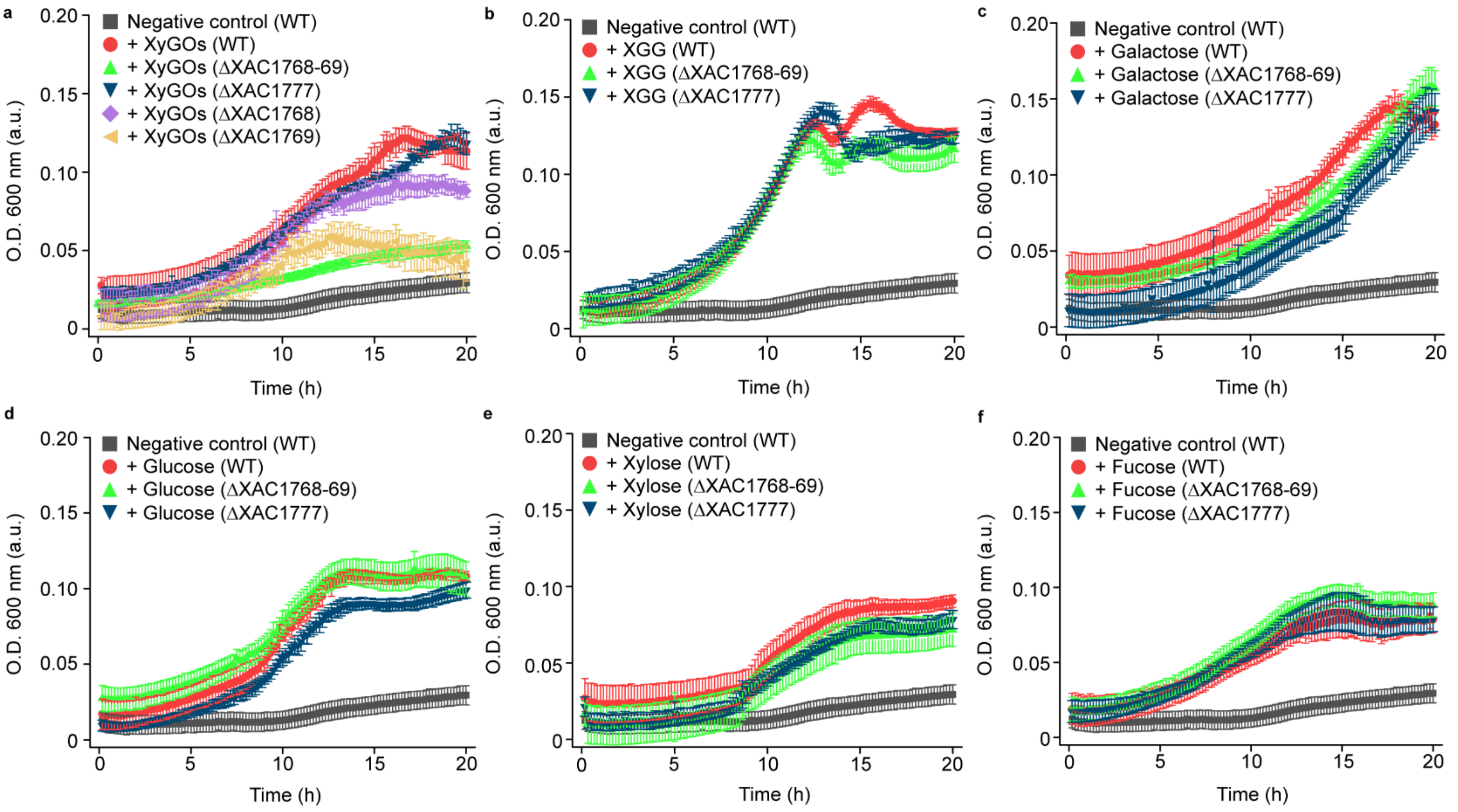

Supplementary Figure 5. Deletion of XyGUL TonB-dependent transporter *cirA* (XAC1769) impaired XyGOs uptake, but the lack of XyGUL MFS transporter did not affect growth. Growth curves of *X. citri* strains (WT in red, ΔXAC1768-69 in green, ΔXAC1768 in lilac, ΔXAC1769 in wheat, ΔXAC1777 in blue) in minimal medium supplemented with XyGOs (a); a mixture of xylose, glucose, and galactose (XGG) in the same molar proportion found in tamarind XyGOs (b); galactose (c); glucose (d); xylose (e); and fucose (f). The OD_{600nm} of WT in minimal medium without carbohydrate was used as a negative control (gray). Data are presented as mean ± SD from three independent experiments (n=3). Data points are shown as filled symbols as described in each legend. Source data are provided as a source datafile.

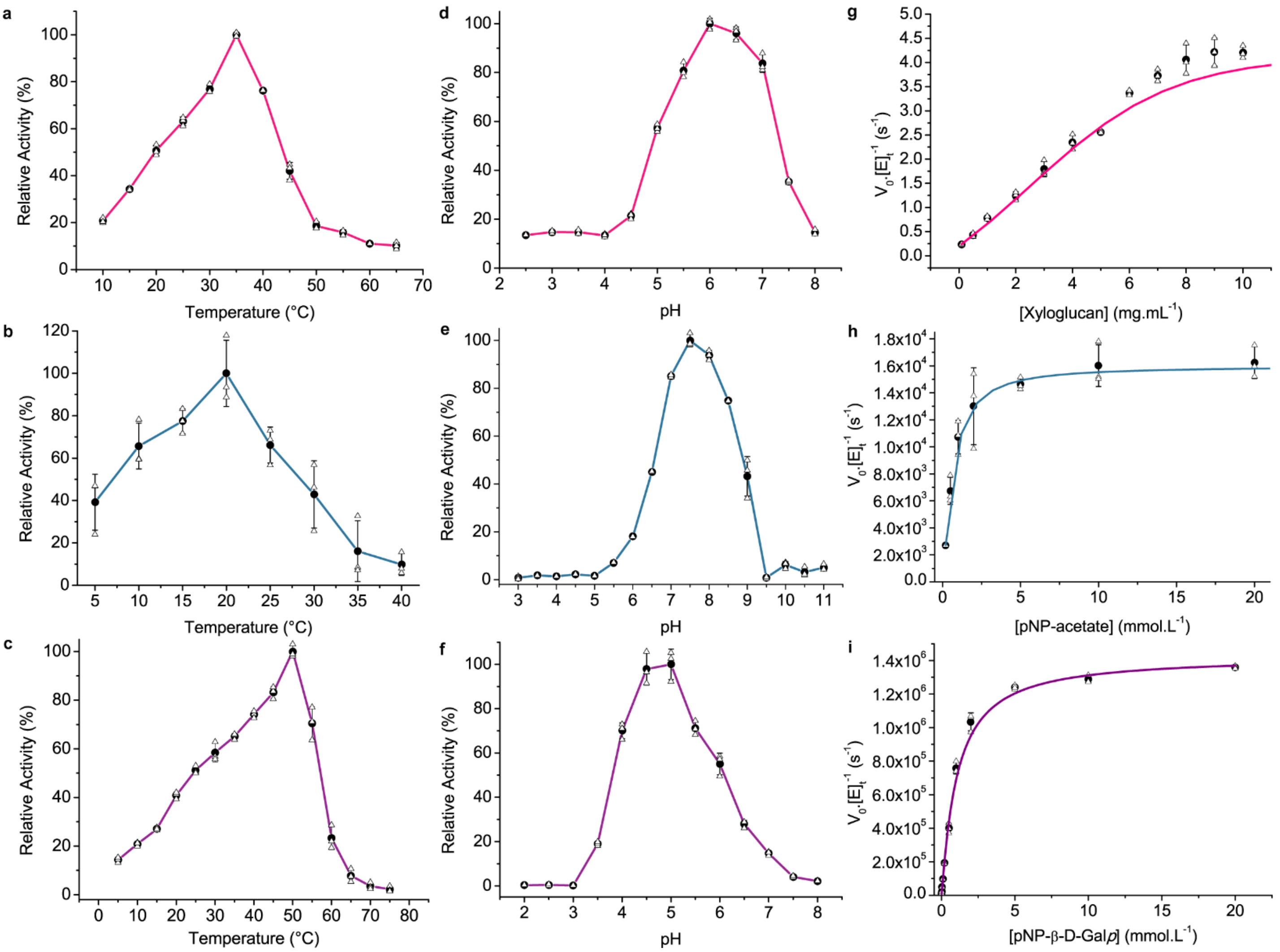

Supplementary Figure 6. Biochemical and kinetic characterization of XyGUL enzymes. *Left*, temperature dependence curves of *Xac*Xeg74 (a), *Xac*XaeA (b) and *Xac*GalD (c). *Middle*, pH dependence curves of *Xac*Xeg74 (d), *Xac*XaeA (e) and *Xac*GalD (f). *Right*, substrate saturation curves of *Xac*Xeg74 using tamarind xyloglucan (g), *Xac*XaeA using *para*-nitrophenyl-acetate (h) and *Xac*GalD using *para*-nitrophenyl-β-D-galactopyranoside (i). Data are shown as mean ± SD from three independent experiments (n=3). (V_0=initial velocity; $[E]_t$=enzyme concentration). Mean values are shown as filled circles, while measured data are shown as empty circles. Standard deviations are shown as black lines. Source data are provided as a source data file.

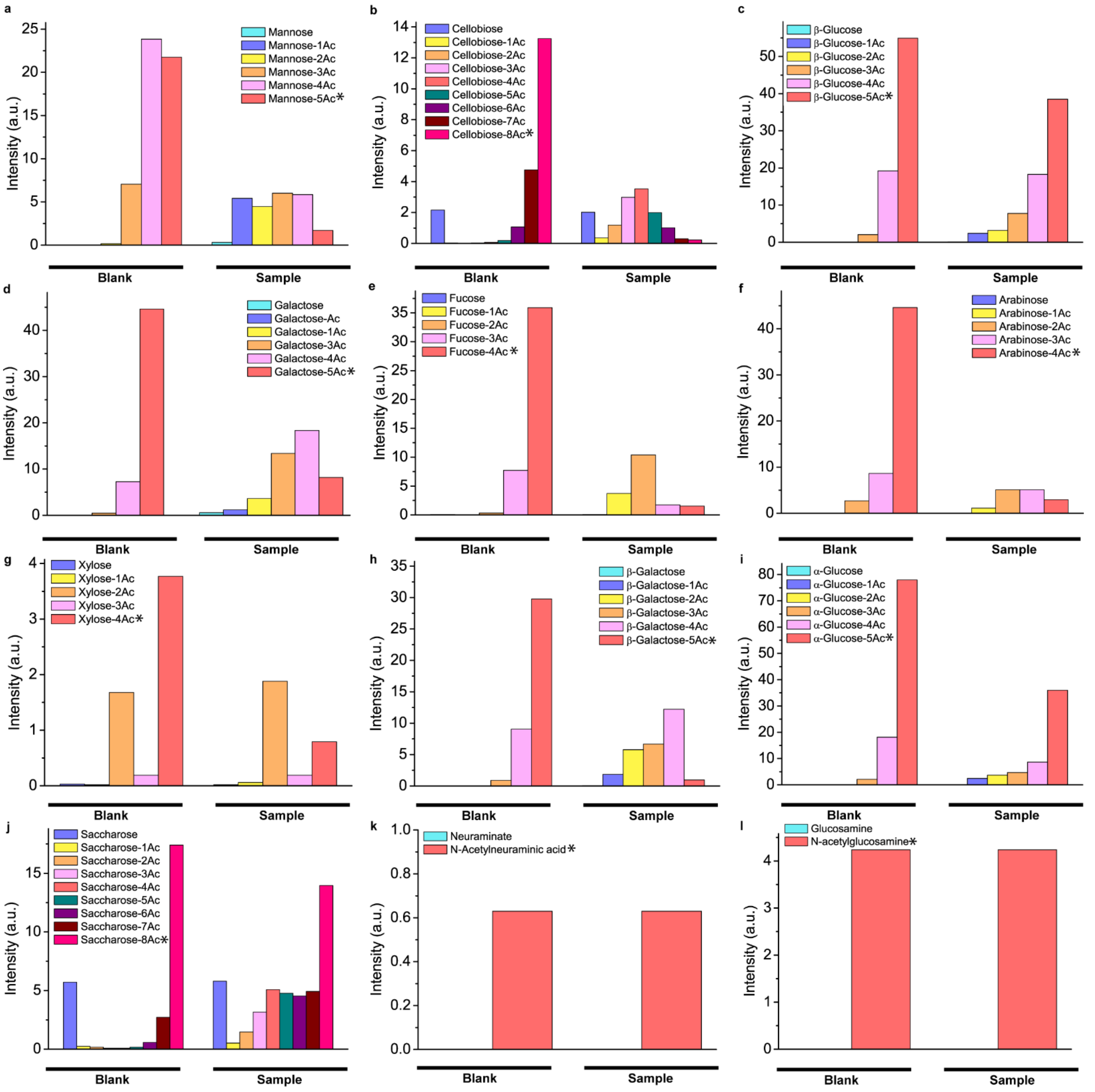

Supplementary Figure 7. Substrate preference of *Xac*XaeA. Fully *O*-acetylated mono- or disaccharides were synthesized and used as a library screening for the enzyme activity detection test. *N*- and *O*-acetylated substrates were also used for the bond type preference analysis. Reactions were carried out at 20° C, 80 mmol.L^{-1} HEPES buffer (pH 7.5) and 600 rpm for 15 min. Samples and blanks (the same respective mix without the enzyme) were collected and added by 40 µL methanol to stop the reaction. A total of 15 µl of the quenched reactions were added to 183 µl of water and 2 µl of xylotetraose (an internal standard used to increase the reliability of the method) and analyzed by ESI (+) mass spectrometry in scan mode (*m*/*z* 150-900) (See Methods for details). Each bar represents the detection of acetylated/non-acetylated carbohydrate species as specified in each legend (the symbol * represents the initial substrate used in each reaction). Ac = acetate.

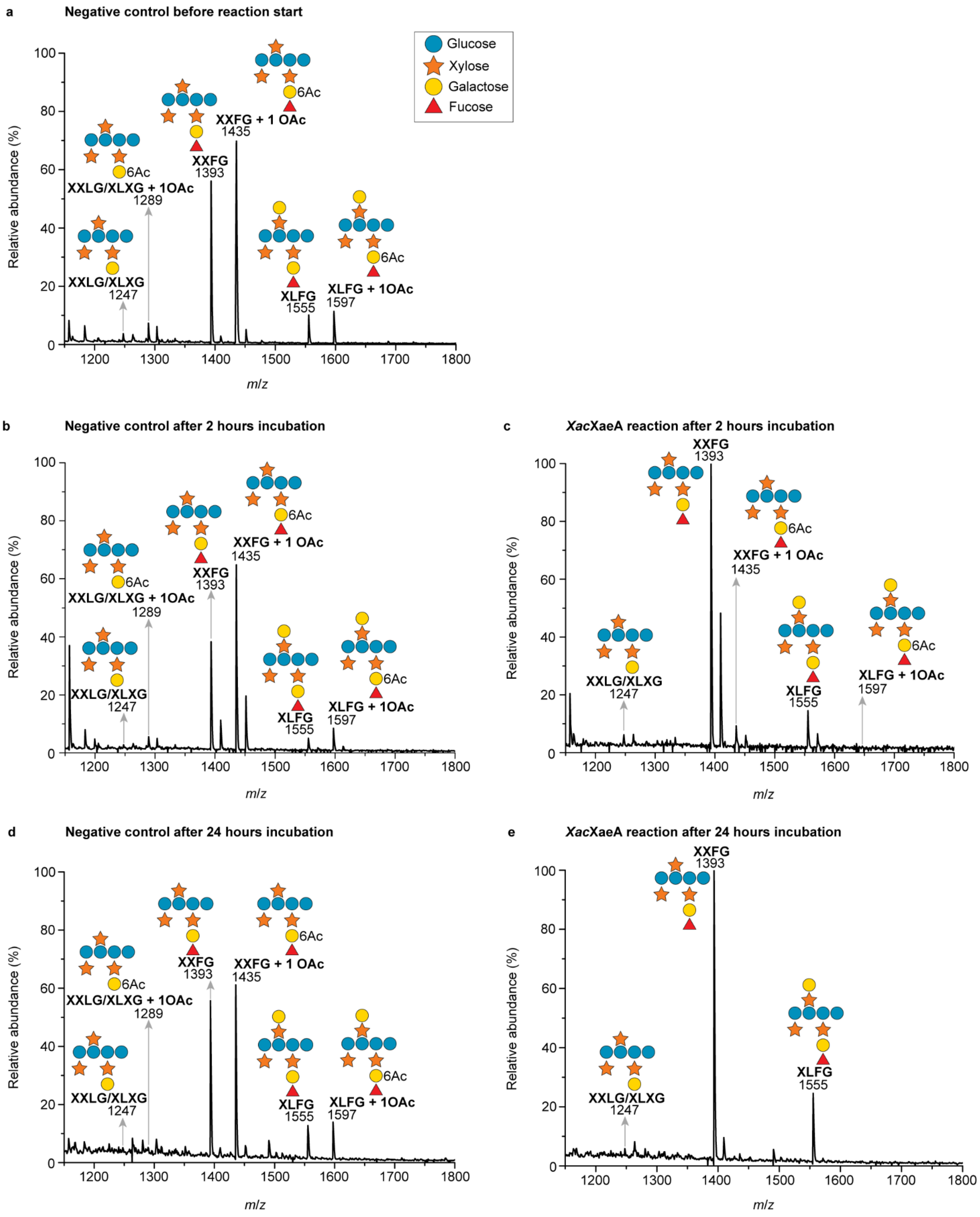

Supplementary Figure 8. *Xac*XaeA activity assay on xyloglucan oligosaccharides. Fucosylated polysaccharides were extracted from *A. thaliana* cell walls and cleaved to oligosaccharides by the action of *Xac*Xeg74. Products were analyzed by ESI(+)-MS/MS and peaks assigned to acetylated or non-acetylated (fuco)galactoxyloglucooligosaccharides considering the literature[3–5]. Negative control before reaction (a), after 2 h (b) and 24 h (d) incubation. *Xac*XaeA reaction after 2 h (c) and 24 h (e) incubation. Note that most of the acetyl moieties were removed in 2 h, indicating that *Xac*XaeA is active on the three identified acetylated oligosaccharides (See Supplementary Table 9 and Supplementary Fig. 9). Carbohydrates are represented by geometric shapes (glucose: blue circles, xylose: orange stars, galactose: yellow circles and fucose: red triangles).

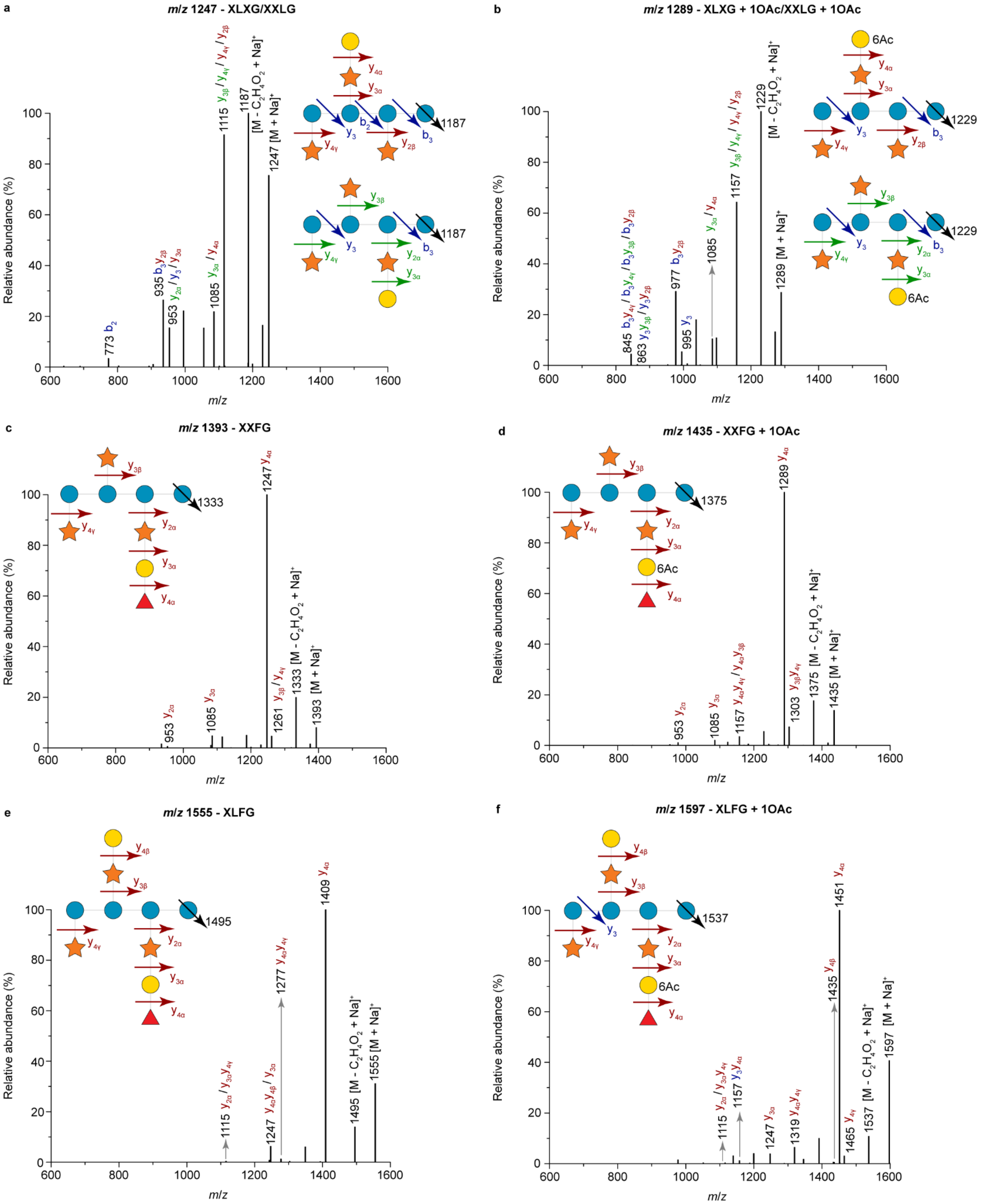

Supplementary Figure 9. Fragmentation patterns of the annotated xyloglucan oligosaccharides assessed by tandem mass spectrometry. In the represented oligosaccharides, arrows indicate the fragmentation position with each ion identified in the corresponding spectra of XLXG/XXLG (a), XXFG (c), XLFG (e) and the acetylated equivalents (b, d, and f, respectively). Internal fragmentations are in black, fragmentations in the main chain are in blue, fragmentations in the side-chains are in red or green to differentiate ions from distinct isomers. See also Supplementary Table 9 and Supplementary Fig. 8. Fragmentations were annotated following the literature[6]. Carbohydrates are represented by geometric shapes (glucose: blue circles, xylose: orange stars, galactose: yellow circles and fucose: red triangles).

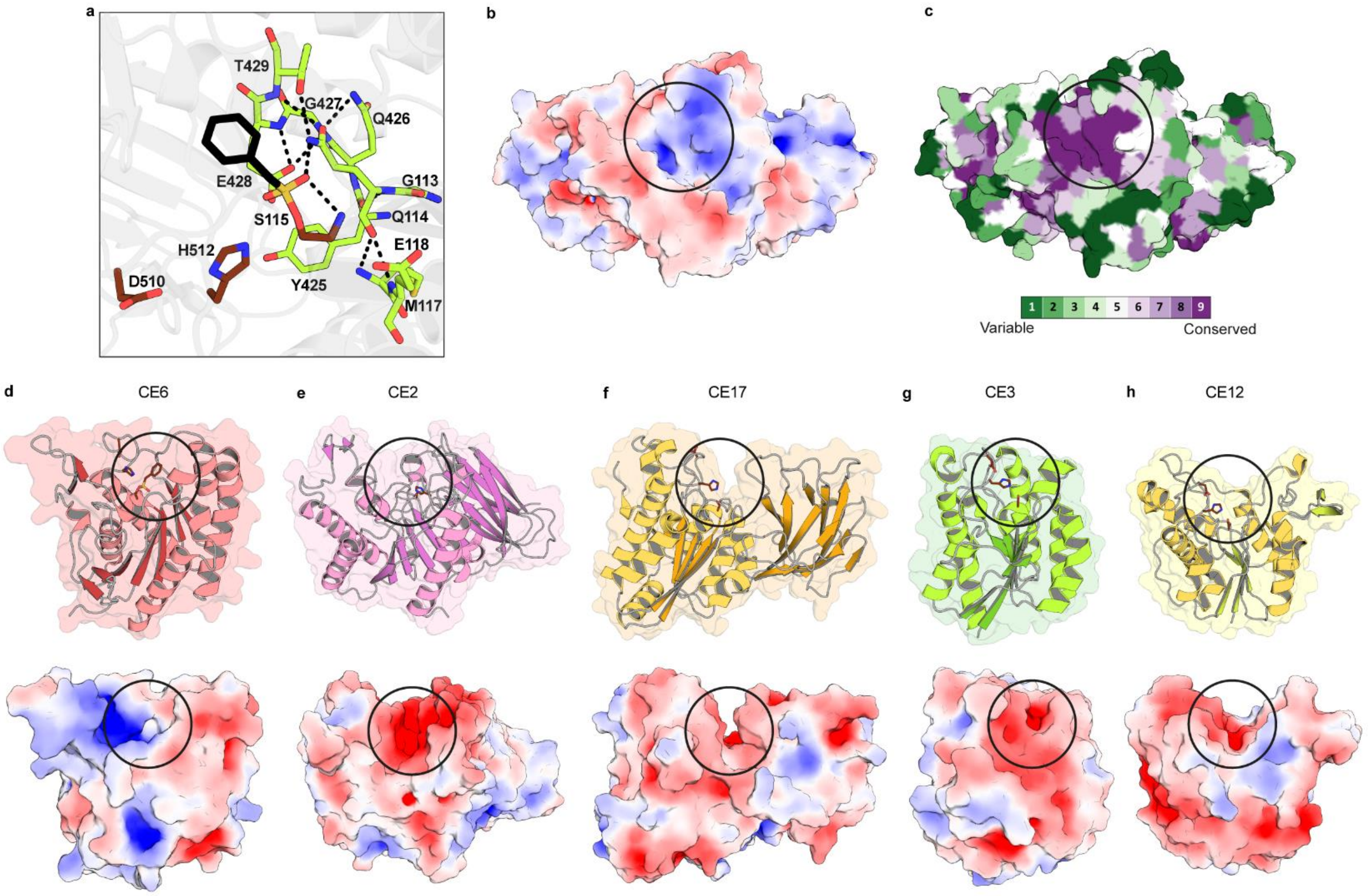

Supplementary Figure 10. CE20 xyloglucan acetylesterase *Xac*XaeA structural analysis. (a) Active site residues represented in sticks. In brown, the C-atoms of the catalytic triad Asp-His-Ser, where the serine is covalently bound to PMSF (black C-atoms) used during the purification steps. C-atoms from the residues forming the oxyanion hole are shown in light green. Dashed lines represent the hydrogen bonds. (b) The electrostatic surface potential (generated using APBS[7]) of *Xac*XaeA structure, colored from blue (positively charged) to red (negatively charged). The black circles delineate the active site. (c) Conservation score derived from ConSurf[8,9], projected over the structure surface representation, with highly conserved amino acid residues in dark purple, semi-conserved residues in white, and variable residues in dark green. (d to h) Representative crystallographic structures from other CAZy carbohydrate esterase families with SGNH-hydrolase fold (cartoon representation) and their respective electrostatic surface potential colored as in (b). CE6 (PDB ID 2APJ[10]) (d), CE2 (PDB ID 3U37[11]) (e), CE17 (PDB ID 6HFZ[12]) (f), CE3 (PDB ID 2VPT[13]) (g) and CE12 (PDB ID 1DEO[14]) (h). (See also Fig. 3).

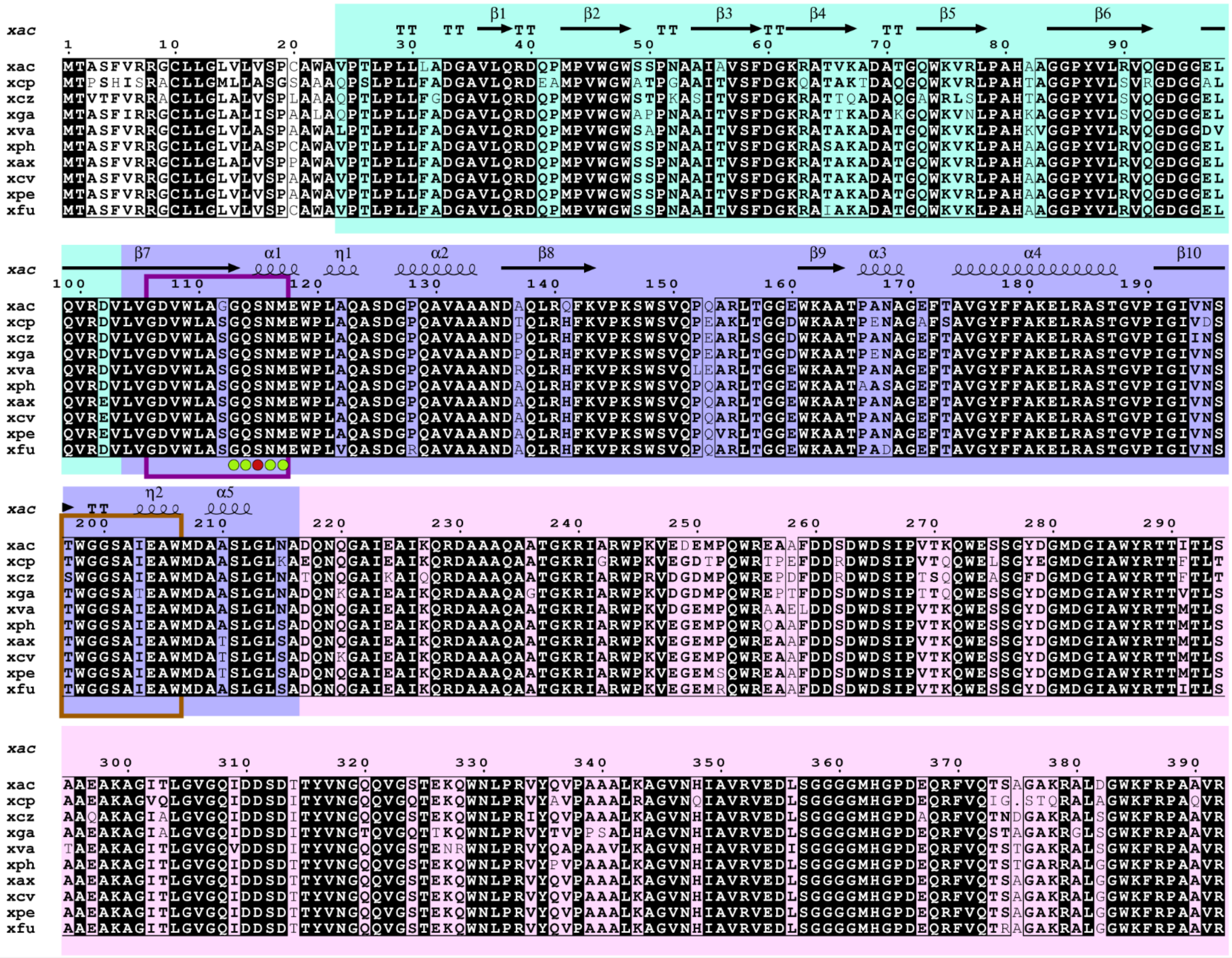

Supplementary Figure 11. Alignment of CE20 xyloglucan acetylesterase sequences from representative *Xanthomonas* species. Sequence numbering based on *Xac*XaeA. Secondary structure elements are represented and labeled according to the crystallographic structure of *Xac*XaeA. Arrows = β-sheets, helices = α-helices and T = β-turn. Each box color represents a specific domain (See Fig. 3): Light green - the β-sandwich domains at the N- and C-termini, light blue - the catalytic core SGNH-hydrolase domain, and light pink - the X448 domain. Green and red circles indicate the residues that form the oxyanion hole and the catalytic triad, respectively. The four boxes delineate the characteristic SGNH hydrolase motifs known as Blocks I (purple), II (brown), III (dark green) and V (yellow). Residues shaded in black are fully conserved. The representative species are: *xac*, *Xanthomonas citri* pv. *citri* 306, *xcp*, *Xanthomonas campestris* pv. *raphani* 756C, *xcx*, *Xanthomonas cucurbitae* ATCC 23378, *xga*, *Xanthomonas gardneri* ICMP 7383, *xva*, *Xanthomonas vasicola* pv. *vasculorum* SAM119, *xph*, *Xanthomonas phaseoli* pv. *phaseoli* CFBP6546R, *xax*, *Xanthomonas axonopodis* pv. *citrumelo* F1, *xcv*, *Xanthomonas campestris* pv. *vesicatoria* 85-10, *xpe*, *Xanthomonas perforans* LH3, *xfu*, *Xanthomonas citri* pv. *fuscans* 4834-R. Alignment was generated with the Clustal Ω[1] server and the figure was produced using the ESPript 3.0 server[2].

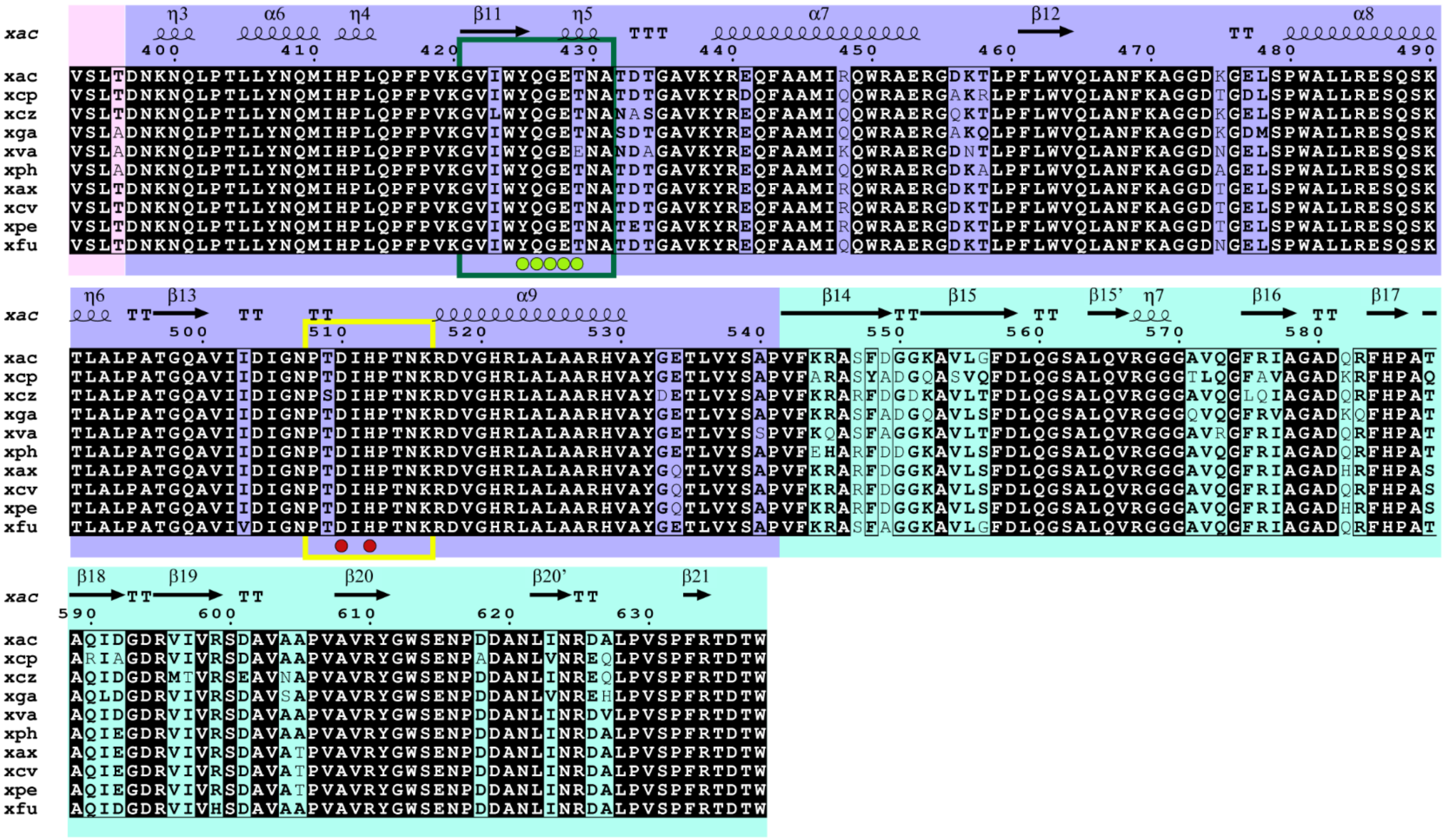

Supplementary Figure 11. Alignment of CE20 xyloglucan acetylesterase sequences from representative *Xanthomonas* species (Continued). Sequence numbering based on *Xac*XaeA. Secondary structure elements are represented and labeled according to the crystallographic structure of *Xac*XaeA. Arrows = β-sheets, helices = α-helices and T = β-turn. Each box color represents a specific domain (See Fig. 3): Light green - the β-sandwich domains at the N- and C-termini, light blue - the catalytic core SGNH-hydrolase domain, and light pink - the X448 domain. Green and red circles indicate the residues that form the oxyanion hole and the catalytic triad, respectively. The four boxes delineate the characteristic SGNH hydrolase motifs known as Blocks I (purple), II (brown), III (dark green) and V (yellow). Residues shaded in black are fully conserved. The representative species are: *xac*, *Xanthomonas citri* pv. *citri* 306, *xcp*, *Xanthomonas campestris* pv. *raphani* 756C, *xcx*, *Xanthomonas cucurbitae* ATCC 23378, *xga*, *Xanthomonas gardneri* ICMP 7383, *xva*, *Xanthomonas vasicola* pv. *vasculorum* SAM119, *xph*, *Xanthomonas phaseoli* pv. *phaseoli* CFBP6546R, *xax*, *Xanthomonas axonopodis* pv. *citrumelo* F1, *xcv*, *Xanthomonas campestris* pv. *vesicatoria* 85-10, *xpe*, *Xanthomonas perforans* LH3, *xfu*, *Xanthomonas citri* pv. *fuscans* 4834-R. Alignment was generated with the Clustal Ω[1] server and the figure was produced using the ESPript 3.0 server[2].

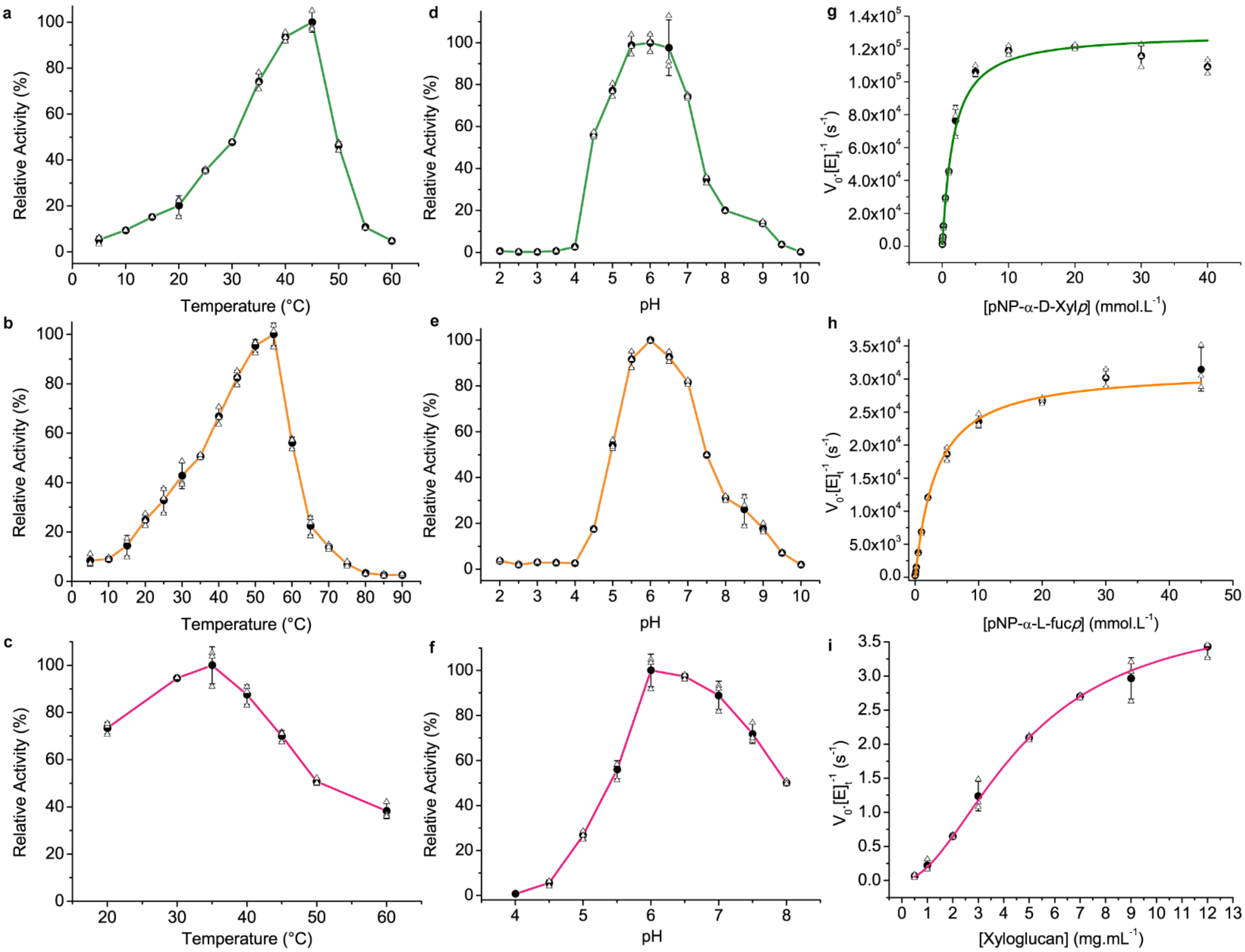

Supplementary Figure 12. Biochemical and kinetic characterization of XyGUL enzymes. *Left*, temperature dependence curves of *Xac*Xyl31 (a), *Xac*Afc95 (b) and *Xac*Egl9 (c). *Middle*, pH dependence curves of *Xac*Xyl31 (d), *Xac*Afc95 (e) and *Xac*Egl9 (f). *Right*, substrate saturation curves of *Xac*Xyl31 using *para*-nitrophenyl-α-D-xylopyranoside (g), *Xac*Afc95 using *para*-nitrophenyl-α-L-fucopyranoside (h) and *Xac*Egl9 using tamarind xyloglucan (i). Curves are colored according to the enzyme: *Xac*Xyl31 (green), *Xac*Afc95 (orange), *Xac*Egl9 (pink). Data are shown as mean ± SD (three independent experiments, n=3). (V_0=initial velocity; $[E]_t$=enzyme concentration). Mean values are shown as filled circles, while measured data are shown as empty circles. Standard deviations are shown as black lines. Source data are provided as a source data file.

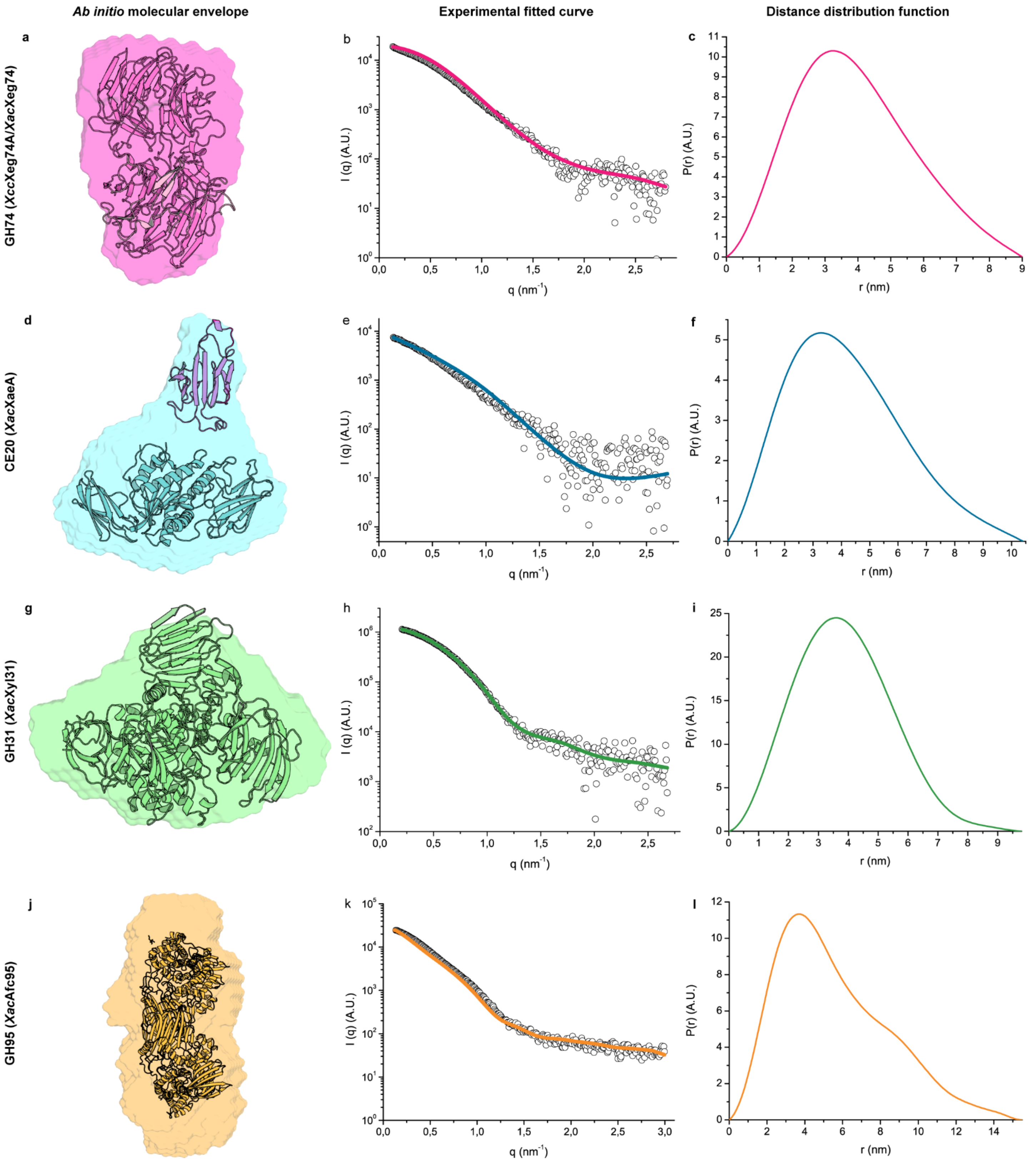

Supplementary Figure 13. SAXS analysis of XyGUL enzymes. *Left*, crystal structures (represented as cartoons) fitted into the SAXS envelopes for *Xac*Xeg74 monomer (in pink), (with crystallographic structure of homologue *Xcc*Xeg74, SeqId 84%) (a), *Xac*XaeA monomer with the core module in a blue cartoon and the homology-modeled X448 domain in light pink (d), *Xac*Xyl31 monomer in green) (g) and *Xac*Afc95 dimer in orange) (j). *Middle*, Experimental (empty circles) and calculated (lines) scattering curves of each enzyme in solution: *Xac*Xeg74 (b), *Xac*XaeA (e), *Xac*Xyl31 (h) and *Xac*Afc95 (k). *Right*, Distance distribution curves used for envelope generation and theoretical scattering calculation based on each experimental curve: *Xac*Xeg74 (c), *Xac*XaeA (f), *Xac*Xyl31 (i) and *Xac*Afc95 (l).

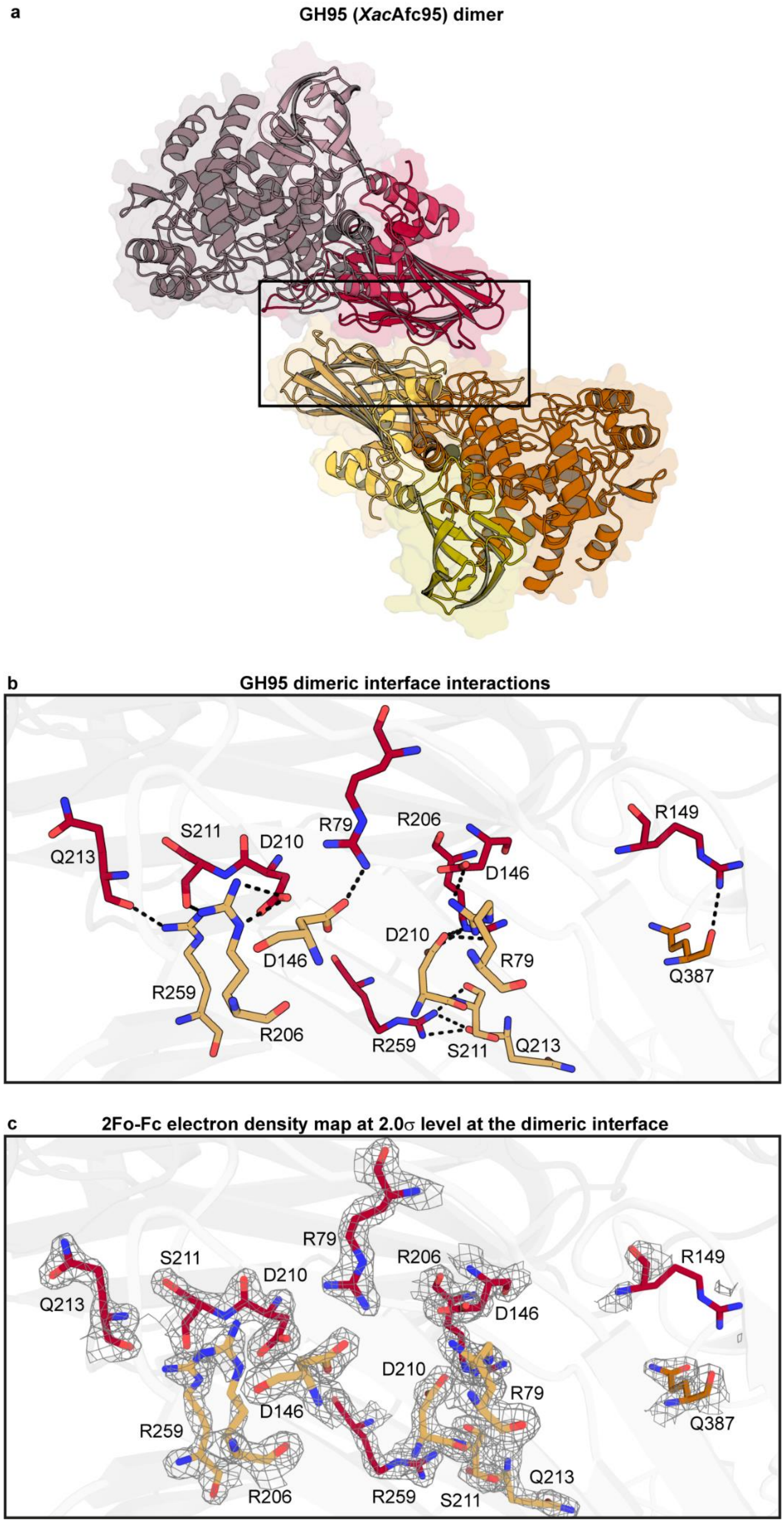

Supplementary Figure 14. GH95 *Xac*Afc95 oligomeric interface. (a) Cartoon representation of the *Xac*Afc95 dimer with C-atoms of each protomer colored in shades of pink and yellow, respectively, to highlight the domains. The rectangle delimits the oligomeric interface. (b) Residues forming the dimeric interface are labeled and represented as sticks with C carbons colored as in (a). Dashed lines represent hydrogen bonds. (c) Dimeric interface as in (b), with $2F_O$-F_C electron density map contoured at 2σ level.

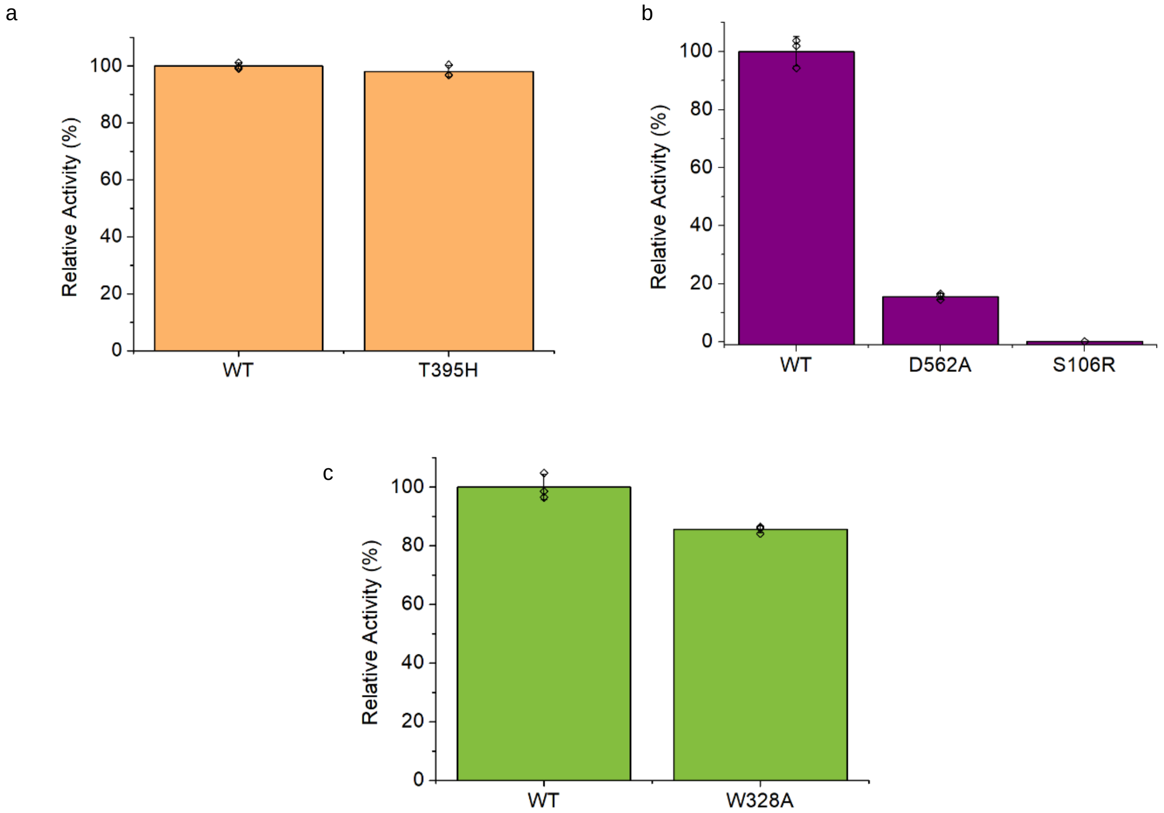

Supplementary Figure 15. Effect of point mutations on the activity of XyGUL enzymes. (a) Wild-type *Xac*Afc95 (WT) and mutant T395H tested with *para*-nitrophenyl-α-L-fucopyranoside. (b) Wild-type *Xac*GalD and mutants D562A and S106R tested with *para*-nitrophenyl-β-D-galactopyranoside. (c) Wild-type *Xac*Xyl31 and mutant W328A tested with *para*-nitrophenyl-α-D-xylopyranoside. Data are shown as mean ± SD from three independent experiments (n=3). Measured data are shown as empty circles. Standard deviations are shown as black lines. Source data are provided as a data file.

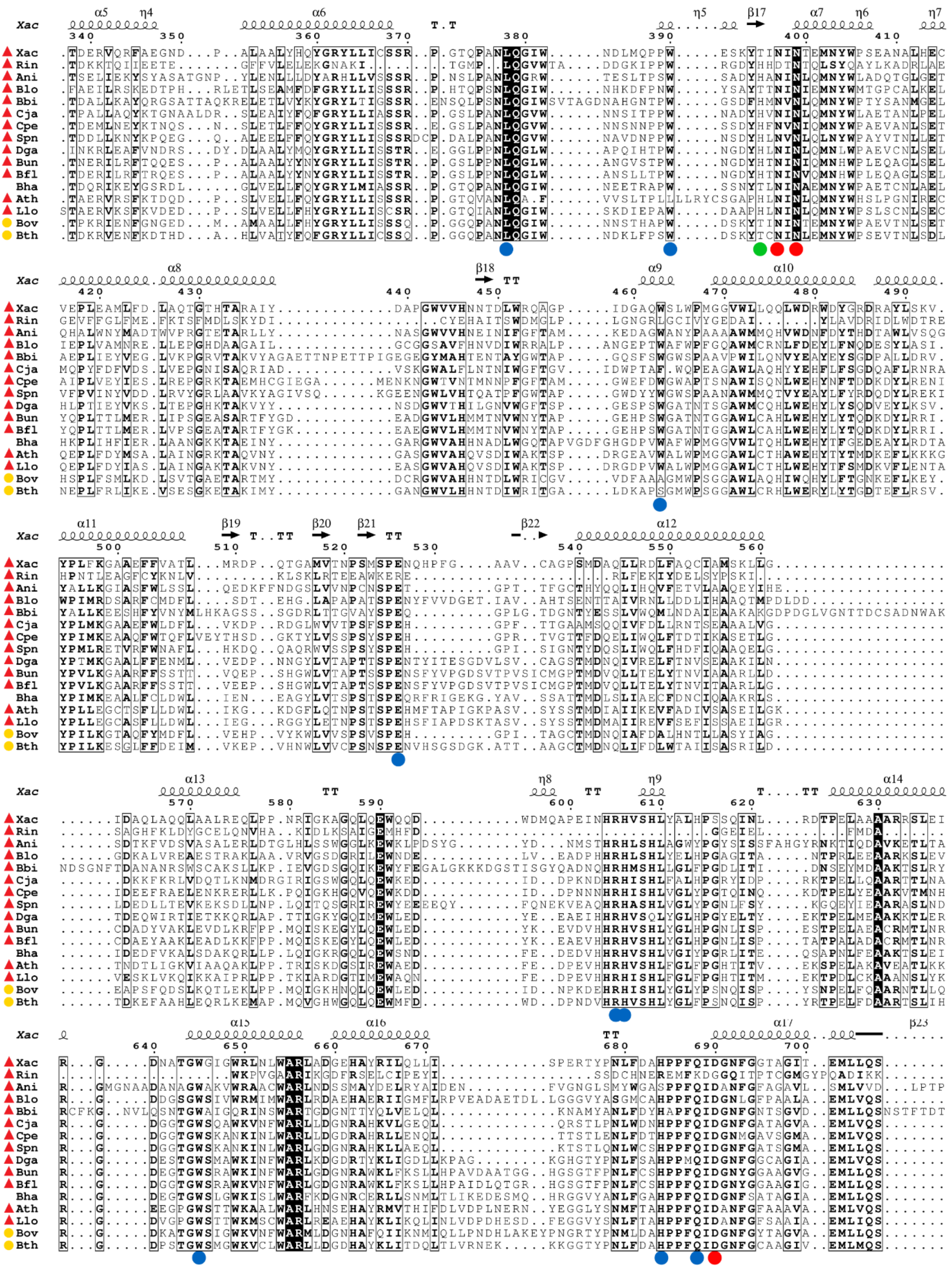

Supplementary Figure 16. Multiple sequence alignment of characterized GH95 members. Residue numbering is based on *Xac*Afc95 sequence. Secondary structure elements are represented according to the crystallographic structure of *Xac*Afc95. Arrows = β-sheets, helices = α-helices and T = β-turn. Red circles represent the catalytic residues. Blue circles mark the active-site residues (as depicted in Fig. 4b). Green circle indicates the polymorphic threonine/histidine. Before each entry, red triangles indicate α-1,2-L-fucosidases (EC 3.2.1.63) and yellow circles indicate α-L-galactosidases (EC 3.2.1.-). The species are: *Xac*, *Xanthomonas citri* pv. *citri* 306, *Rin*, *Roseburia inulinivorans* DSM 16841, *Ani*, *Aspergillus nidulans* FGSC A4, *Blo*, *Bifidobacterium longum* subsp. infantis ATCC 15697 = JCM 1222 = DSM 20088, *Bbi*, *Bifidobacterium bifidum* JCM 1254, *Cja*, *Cellvibrio japonicus* Ueda107, *Cpe*, *Clostridium perfringens* ATCC 13124, *Spn*, *Streptococcus pneumoniae* TIGR4, *Dga*, *Dysgonomonas gadei* ATCC BAA-286, *Bun*, *Bacteroides uniformis* ATCC 8492, *Bfl*, *Bacteroides fluxus* YIT 12057, *Bha*, *Bacillus halodurans* C-125, Ath, *Arabidopsis thaliana*, *Llo*, *Lilium longiflorum*, *Bov*, *Bacteroides ovatus* ATCC 8483, *Bth*, *Bacteroides thetaiotaomicron* VPI-5482. Alignment performed with Clustal Ω[1] server and image produced with ESPript 3.0 server[2].

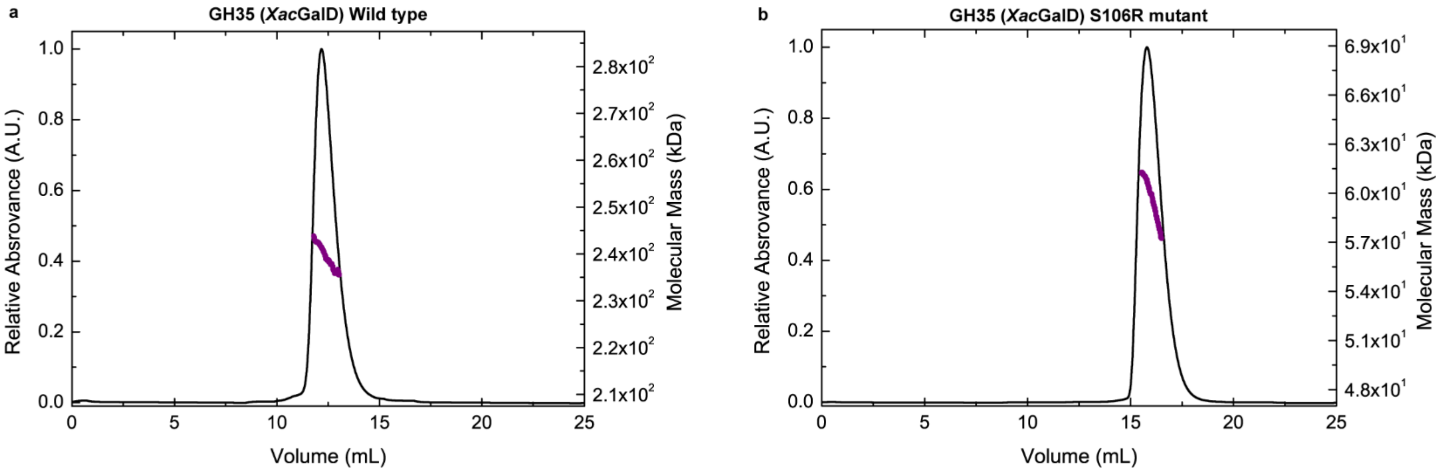

Supplementary Figure 17. Mutation S106R disrupts the GH35 *Xac*GalD tetramer. Size-exclusion chromatography with multiple-angle light scattering (SEC-MALS) analysis of (a) wild-type *Xac*GalD (tetramer) and (b) S106R mutant (monomer). Black curves represent the relative absorbance at 280 nm of the proteins eluted from a Superdex 200 HR 10/300 GL analytical size-exclusion column. Purple lines represent the molecular mass of the eluted proteins calculated from the multiple-angle light scattering measurements.

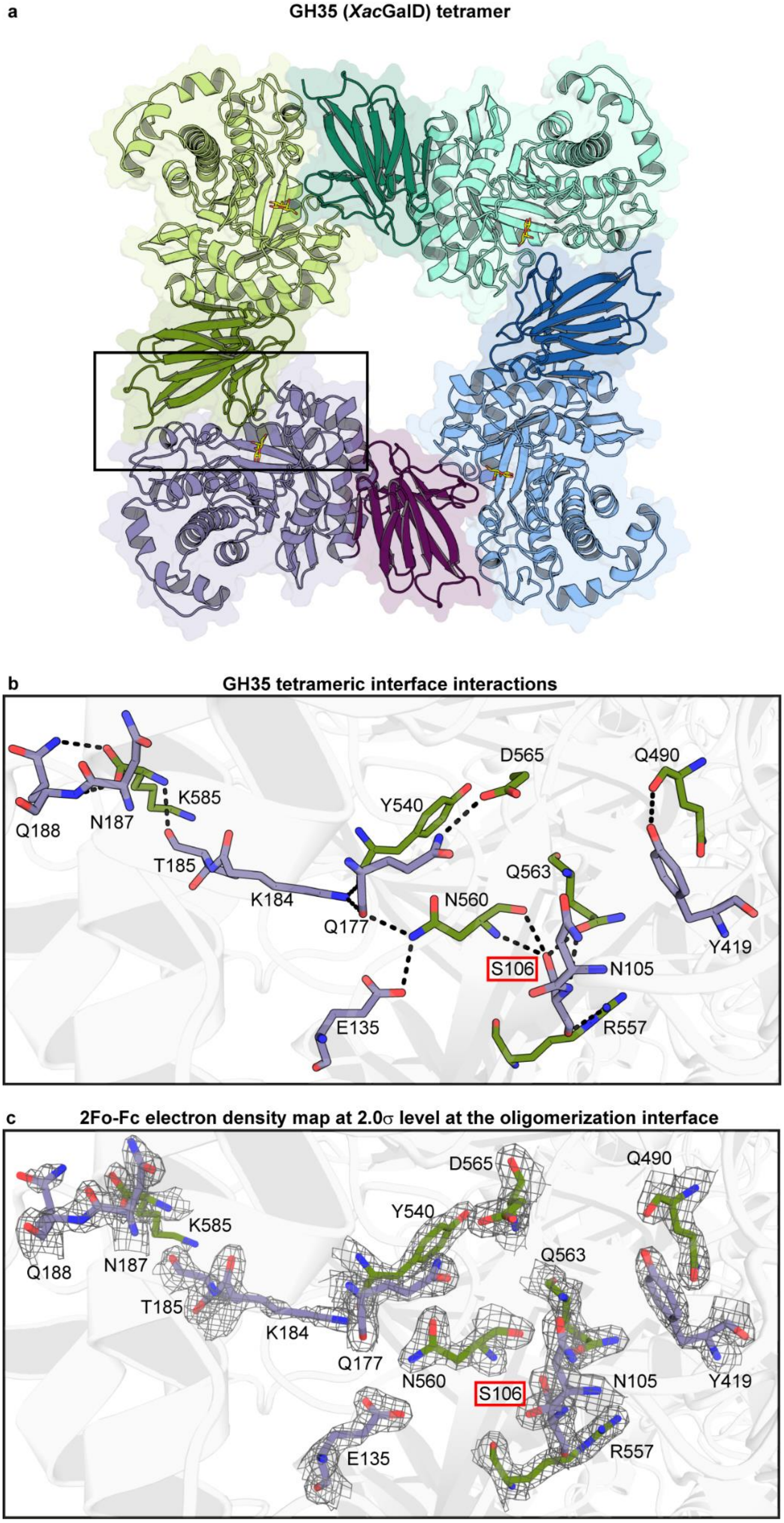

Supplementary Figure 18. GH35 *Xac*GalD tetramer. (a) Crystal structure of *Xac*GalD tetramer (cartoon) with each protomer represented in shades of a distinct color to highlight the catalytic domain (light shades) and the β-sandwich domain (dark shades). Sticks represent D-galactose (yellow C-atoms). The rectangle delimits the oligomeric interface, repeated between every two protomers. (b) Amplified view of the oligomeric interface with residues represented as sticks and C-atoms colored as in (a). Dashed lines represent hydrogen bonds. The residue S106, which disrupts the interface when mutated to arginine, is marked with a red box. (c) Oligomeric interface as in (b), with $2F_O$-F_C electron density map contoured at 2σ level.

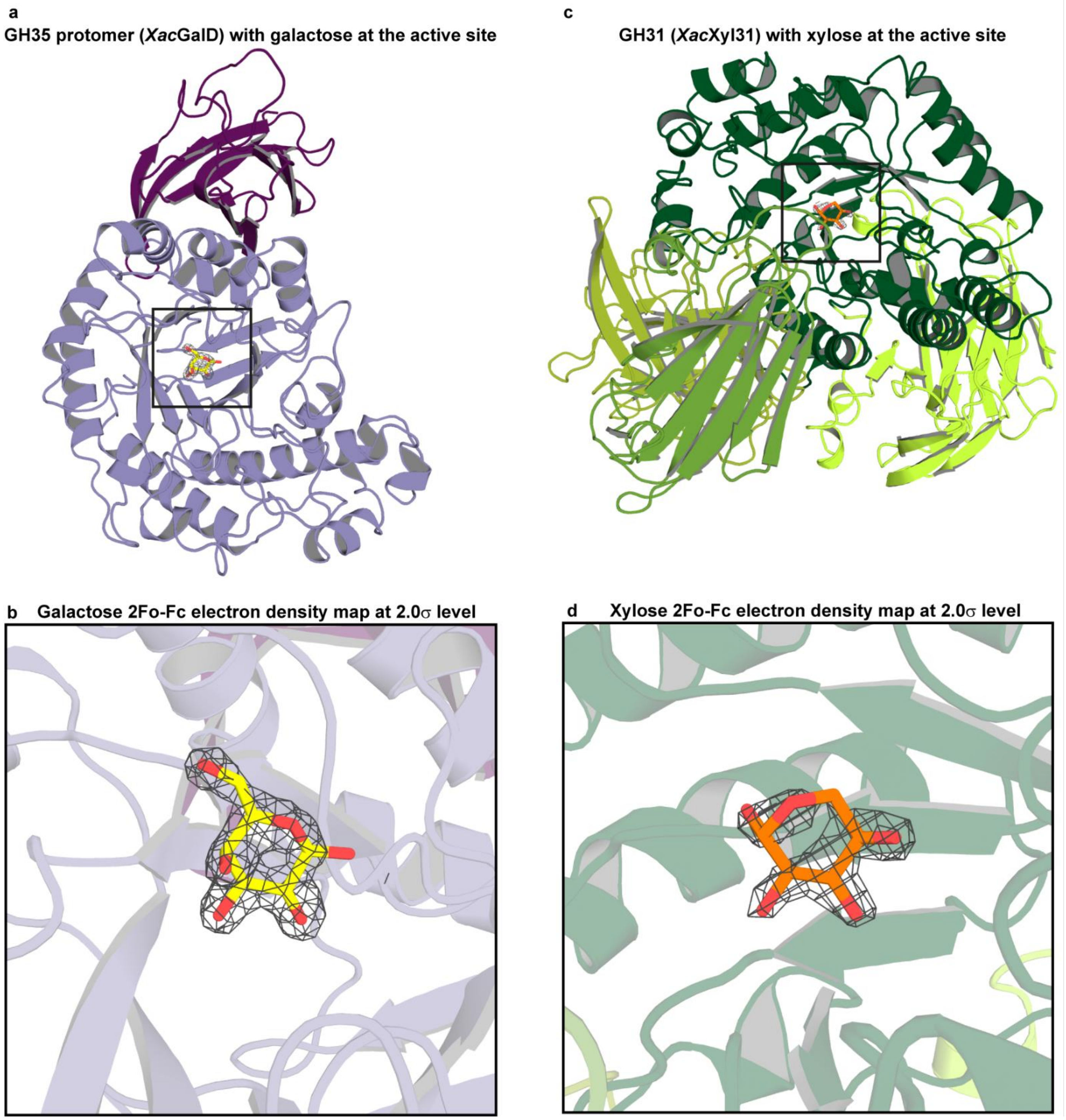

Supplementary Figure 19. GH35 *Xac*GalD and GH31 *Xac*Xyl31 crystallographic structures in complex with products. *Xac*GalD (a) and *Xac*Xyl31 (c) protomers are represented in cartoon, with the bound galactose (yellow C-atoms) or xylose (orange C-atoms) shown as sticks, respectively. Domains are colored in shades of purple in *Xac*GalD or green in *Xac*Xyl31. The $2F_O$-F_C electron density maps contoured at 2σ level of galactose (b) and xylose (d) in the active site of *Xac*GalD and *Xac*Xyl31, respectively.

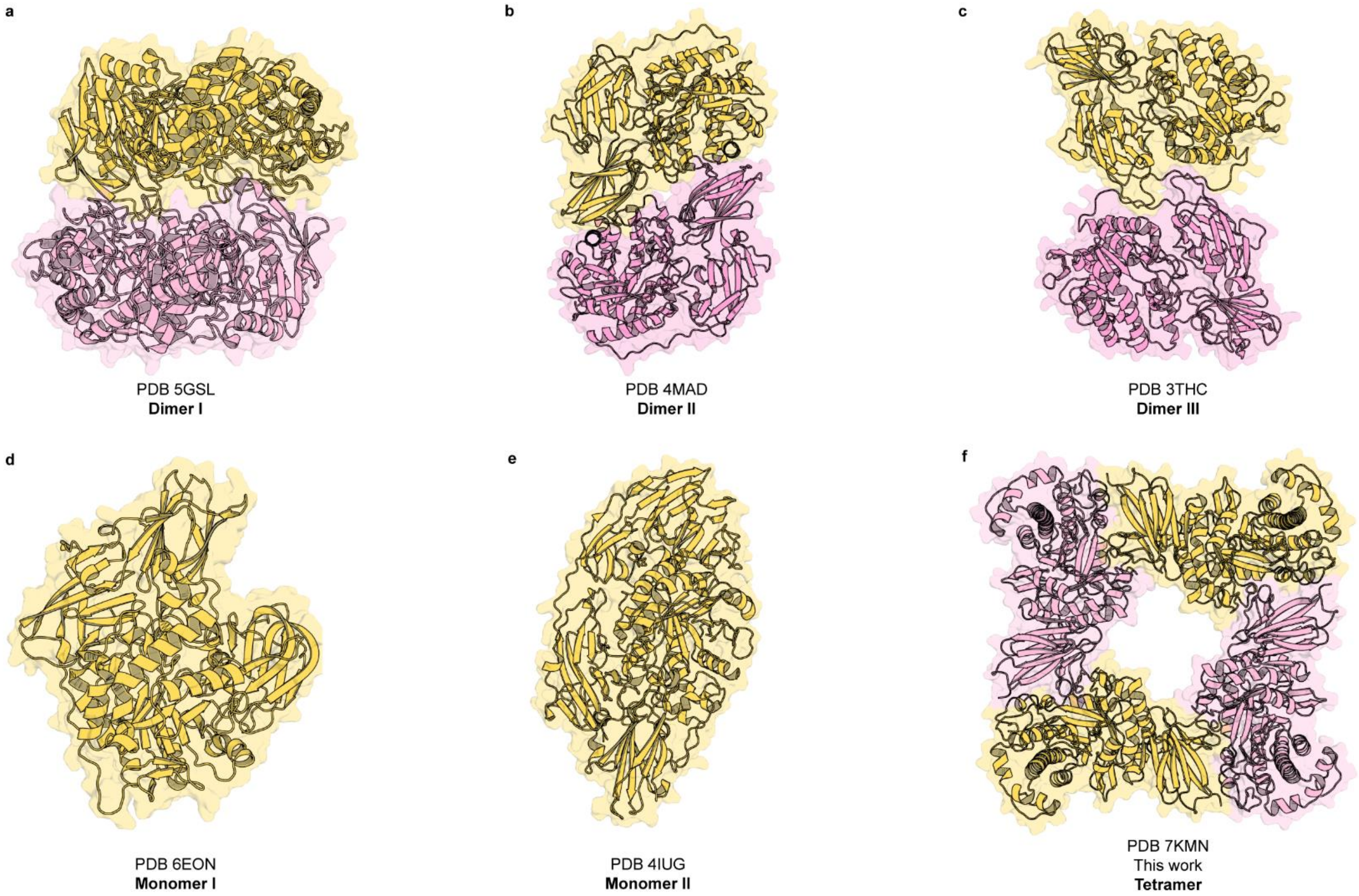

Supplementary Figure 20. The diversity of oligomeric arrangements in the GH35 family. Each protomer (or monomer) is represented in yellow or pink cartoon and transparent surface. Structural analysis is also provided in Supplementary Table 11. (a) Archaea - *Pyrococcus horikoshii* OT3 (PDB 5GSL) dimer interface type I, (b) Firmicutes - *Bacillus circulans* ATCC 31382 (PDB 4MAD[15]) dimer interface type II, (c) Chordate - *Homo sapiens* (PDB 3THC[16]) dimer interface type III, (d) Bacteroidetes - *Bacteroides thetaiotaomicron* VPI-5482 (PDB 6EON[17]) monomer type I (with 3 accessory domains), (e) Fungi - *Aspergillus oryzae* RIB40 (PDB 4IUG[18]) monomer type II (with 4 accessory domains), and (f) Proteobacteria - *Xanthomonas citri* pv. *citri* 306 tetramer (this work, Fig. 4).

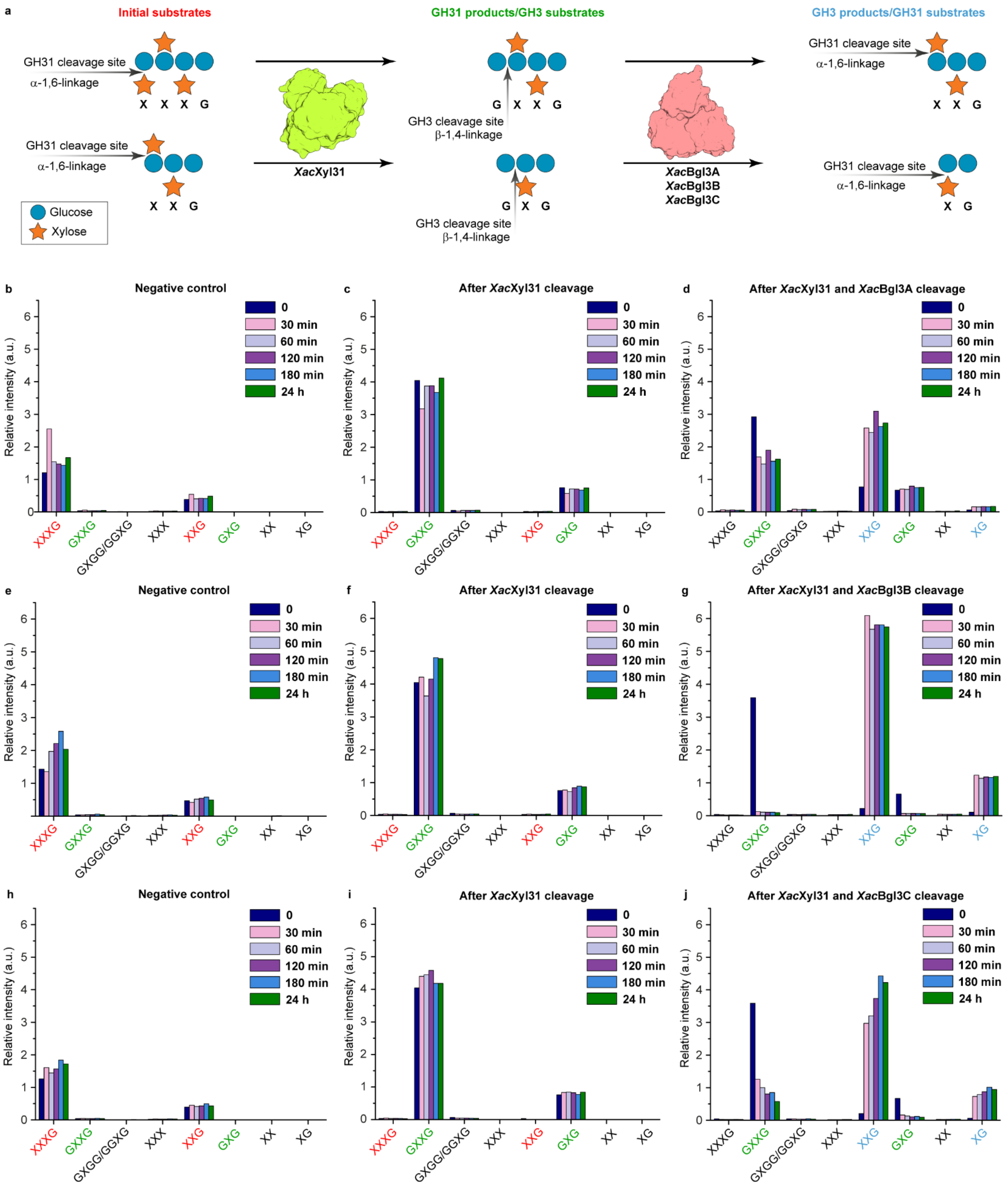

Supplementary Figure 21. GH3 β-glucosidases from *X. citri* are active on xyloglucan oligosaccharides pre-treated with *Xac*Xyl31. (a) Schematic representation of the XyGOs used as substrates (*left*) for *Xac*Xyl31 (green surface), which removes the xylosyl moiety at the non-reducing end, generating products (*middle*) that are substrates for GH3 β-glucosidases (pink surface), which remove the glycosyl moiety at the non-reducing end from the backbone, releasing shorter substrates for the further action of *Xac*Xyl31 (*right*). (b-j) Initial substrates (red labels), *Xac*Xyl31 products (green labels), and GH3 products (cyan labels) detected by mass spectrometry at several incubation times without enzyme (negative control, b, e and h*), with Xac*Xyl31 (c, f and i), and after thermal inactivation of *Xac*Xyl31 and subsequent incubation with each one of the three GH3 β-glucosidases from *X. citri* (d, g and j). The relative intensity of the peaks was normalized using an internal standard. Carbohydrates are represented by geometric shapes (glucose: blue circles and xylose: orange stars).

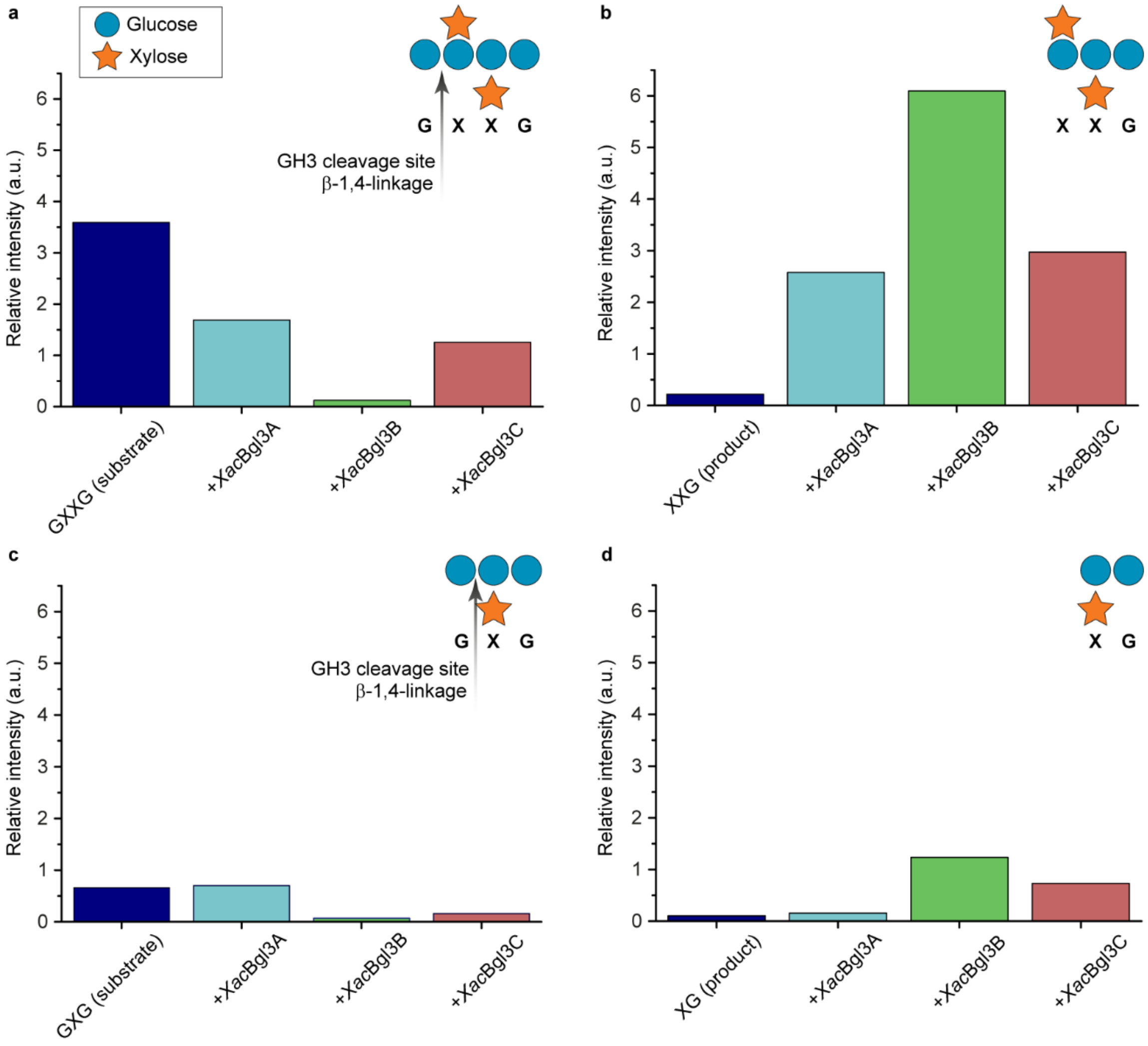

Supplementary Figure 22. A simplified view of the results shown in the previous Supplementary Fig. 21, highlighting substrate depletion (a and c) and product generation (b and d) after 30 min incubation with 100 µg of GH3 β-glucosidases from *X. citri*. The negative control (reaction without enzyme) is shown in blue and the other reactions are colored according to the respective enzyme indicated in the X axis. The relative intensity of the peaks was normalized using an internal standard. Carbohydrates are represented by geometric shapes (glucose: blue circles and xylose: orange stars).

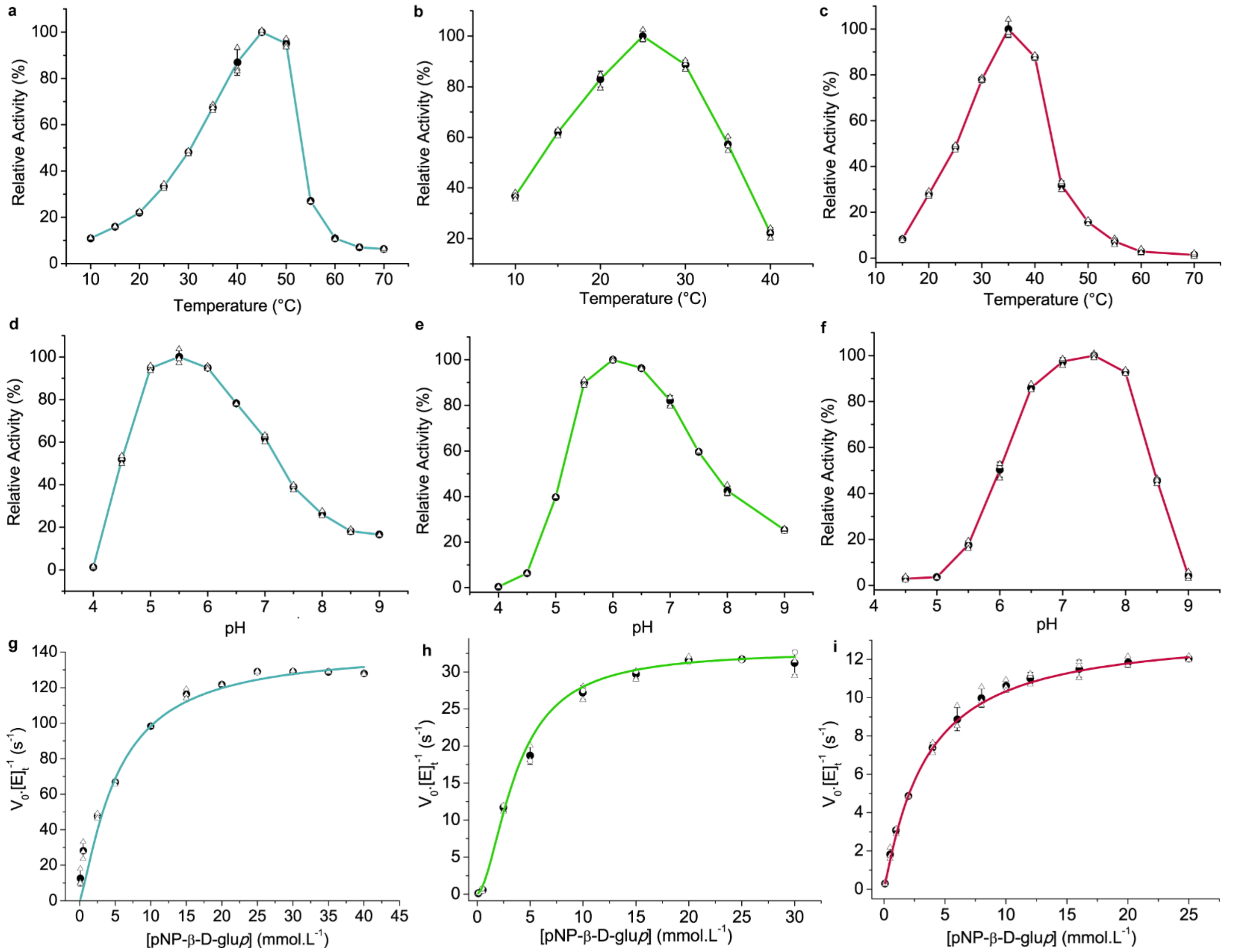

Supplementary Figure 23. Biochemical characterization of the GH3 β-glucosidases *Xac*Bgl3A, *Xac*Bgl3B and *Xac*Bgl3C. Temperature dependence curves of *Xac*Bgl3A (a), *Xac*Bgl3B (b) and *Xac*Bgl3C (c). pH dependence curves of *Xac*Bgl3A (d), *Xac*Bgl3B (e) and *Xac*Bgl3C (f). Substrate saturation curves using *para*-nitrophenyl-β-D-glucopyranoside for *Xac*Bgl3A (g), *Xac*Bgl3B (h) and *Xac*Bgl3C (i). The curves are colored according to the enzyme: *Xac*Bgl3A (light blue), *Xac*Bgl3B (green) and *Xac*Bgl3C (red). Data are shown as mean ± SD from three independent experiments (n=3). (V_0=initial velocity and $[E]_t$=enzyme concentration). Mean values are shown as filled circles, while measured data are shown as empty circles. Standard deviations are shown as black lines. Source data are provided as a data file.

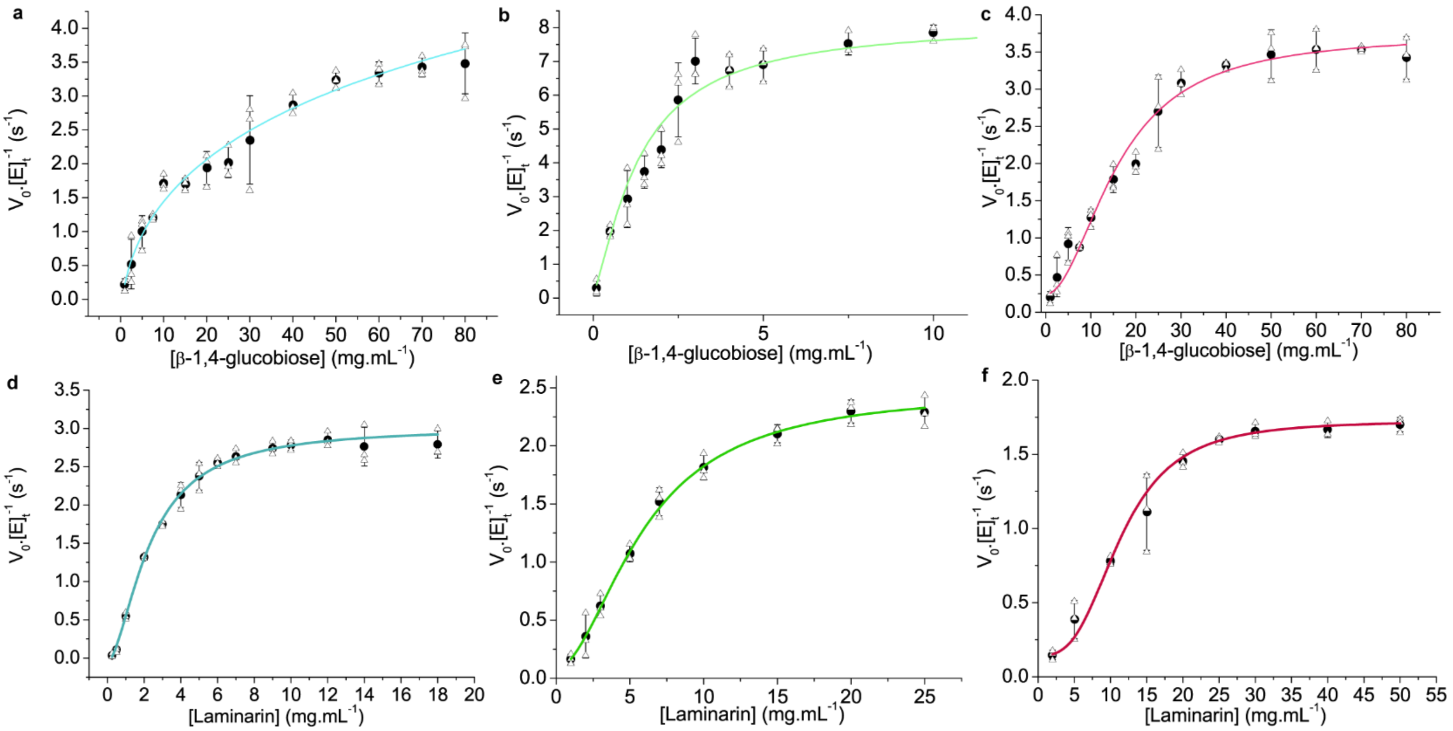

Supplementary Figure 24. Biochemical characterization of the GH3 β-glucosidases *Xac*Bgl3A, *Xac*Bgl3B and *Xac*Bgl3C. Substrate saturation curves using β-1,4-glucobiose for *Xac*Bgl3A (a), *Xac*Bgl3B (b) and *Xac*Bgl3C (c). Substrate saturation curves using polymeric laminarin for *Xac*Bgl3A (d), *Xac*Bgl3B (e) and *Xac*Bgl3C (f). Note that these enzymes cleave both β-1,4 and β-1,3 linkages between glucosyl residues from cellooligosaccharides (β-1,4) or laminarin (β-1,3), indicating they might play a role in the final steps not only of XyG or cellulose depolymerization but also of other glucans such as callose (β-1,3 glucan backbone). The curves are colored according to the enzyme: *Xac*Bgl3A (light blue), *Xac*Bgl3B (green) and *Xac*Bgl3C (pink). Data are shown as mean ± SD from three independent experiments (n=3). (V_0=initial velocity and $[E]_t$=enzyme concentration). Mean values are shown as filled circles, while measured data are shown as empty circles. Standard deviations are shown as black lines. Source data are provided as a data file.

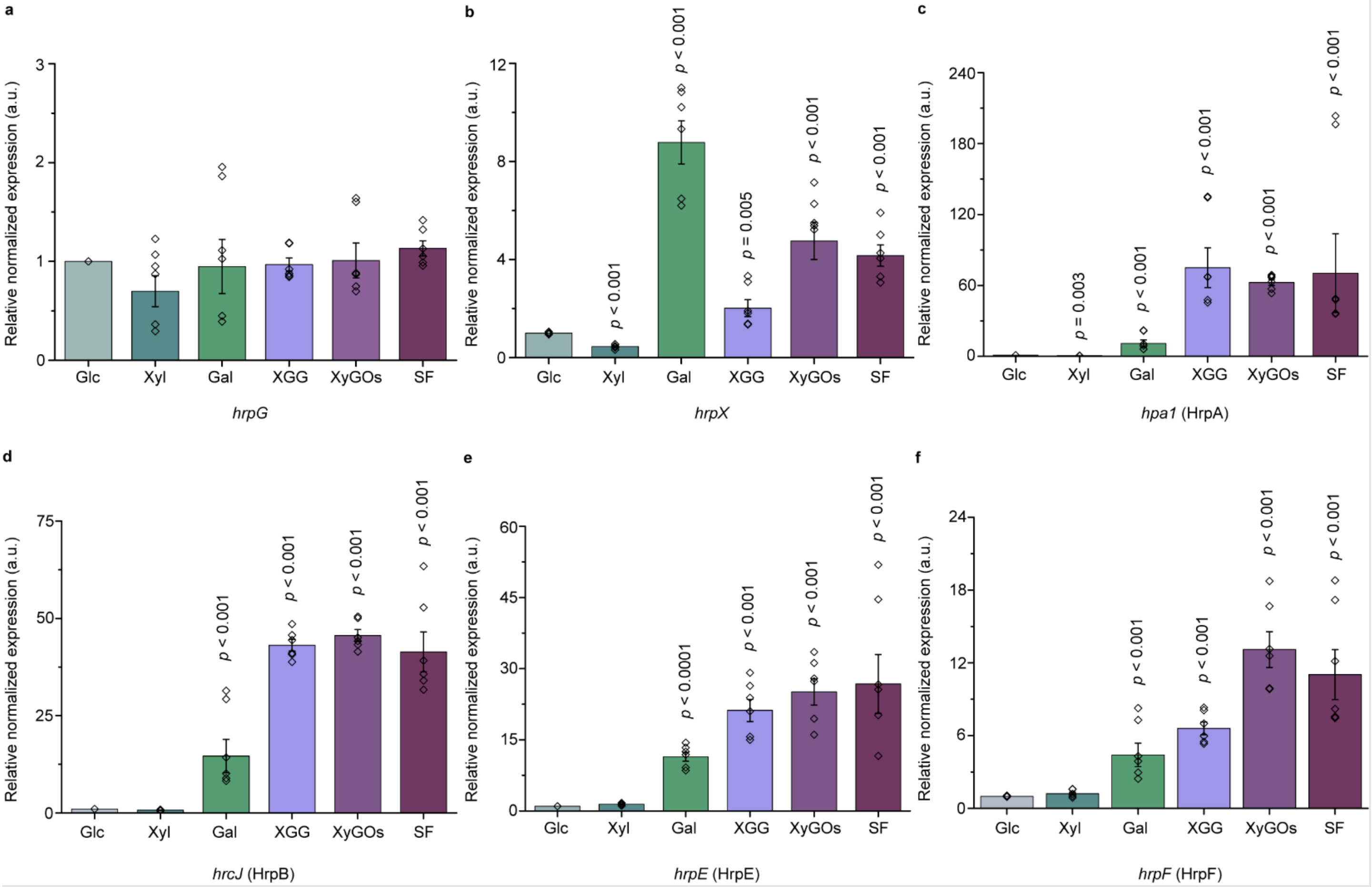

Supplementary Figure 25. Expression profile of *hrpG*, *hrpX* and representative genes of the *hrp* cluster of *X. citri* under different culture conditions. RT-qPCR data analysis showing the normalized relative expression of *hrpG*, *hrpX*, *hpa1*, *hrcJ*, *hrpE* and *hrpF* genes in a minimal medium XVM2[19], termed here as SF (saccharose plus fructose), or modified XVM2 in which sucrose and fructose were replaced by different carbohydrates: Glc, glucose; Xyl, xylose; Gal, galactose; XGG, sugar mix of xylose, galactose and glucose; and XyGOs, xyloglucan oligosaccharides. Bars are colored according to the respective condition indicated in the X axis. Values were normalized using two experimentally validated reference genes (XAC4218 and XAC2177). Data are shown as mean values (± SEM) from three independent biological experiments with technical duplicates. *p*-values indicate significant differences compared to the Glc condition as determined by two-tailed *t*-test. The operons corresponding to each gene are indicated in parentheses. Measured data are shown as empty circles. SEM values are shown as black lines. Source data are provided as a source data file.

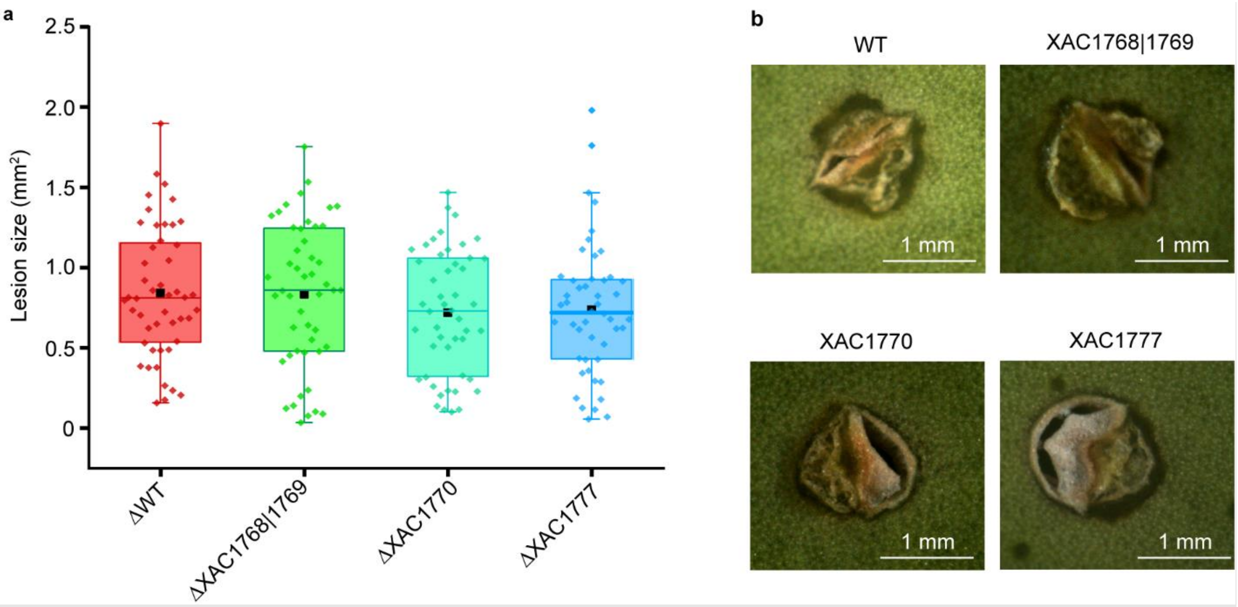

Supplementary Figure 26. The knockout of selected XyGUL genes is insufficient to abolish virulence. (a) Box plot showing the area of canker lesions resulting from pinprick inoculations with *X. citri* in *Citrus sinensis* leaves imaged at 12 days post-infection. Each box plot presents the median (horizontal line inside the box), the mean value (black square), the first (Q_1) and third (Q_3) quartiles (lower and upper edges of the box), and whiskers (vertical lines) extending to the most extreme values inside lower [$Q_1 - 1.5(Q_3\text{-}Q_1)$] and upper [$Q_3 + 1.5(Q_3\text{-}Q_1)$] fences. Dots represent measured data and those outside the whiskers are outliers. (b) A representative image of the lesion promoted by each strain. WT = wild-type. The assays were performed with three independent biological samples, each composed of 16 technical replicates. Tukey test indicates no significant difference between knockout and WT strains at 0.05 level.

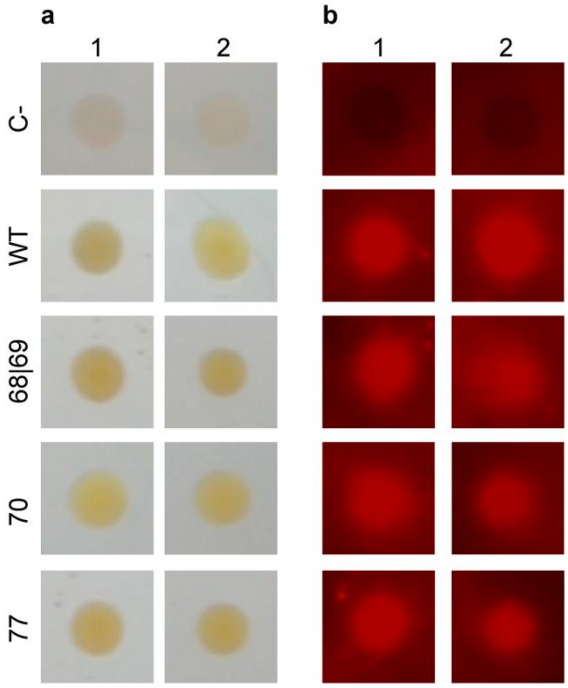

Supplementary Figure 27. The deletion of *Xac*Xeg74 does not abolish the xyloglucanase activity of *X. citri*. (a) Colonies of *X. citri* strains and *E. coli* (C-) growth during 30 h in NYG medium containing 0.5% tamarind seed xyloglucan. 68|69=ΔXAC1768-69 (TonB-dependent transporters); 77=ΔXAC1777 (MFS transporter) and 70=ΔXAC1770 (GH74 xyloglucanase). (b) The same plate showed in panel (a) stained with congo red after removal of bacterial colonies to evidence the clearer halo corresponding to xyloglucan degradation. Note that the xyloglucan degradation halo is absent in the negative control (C-) and is not affected by the deletion of XyGUL genes, including XAC1770, which encodes a GH74 xyloglucanase. The plate is representative of three independent assays.

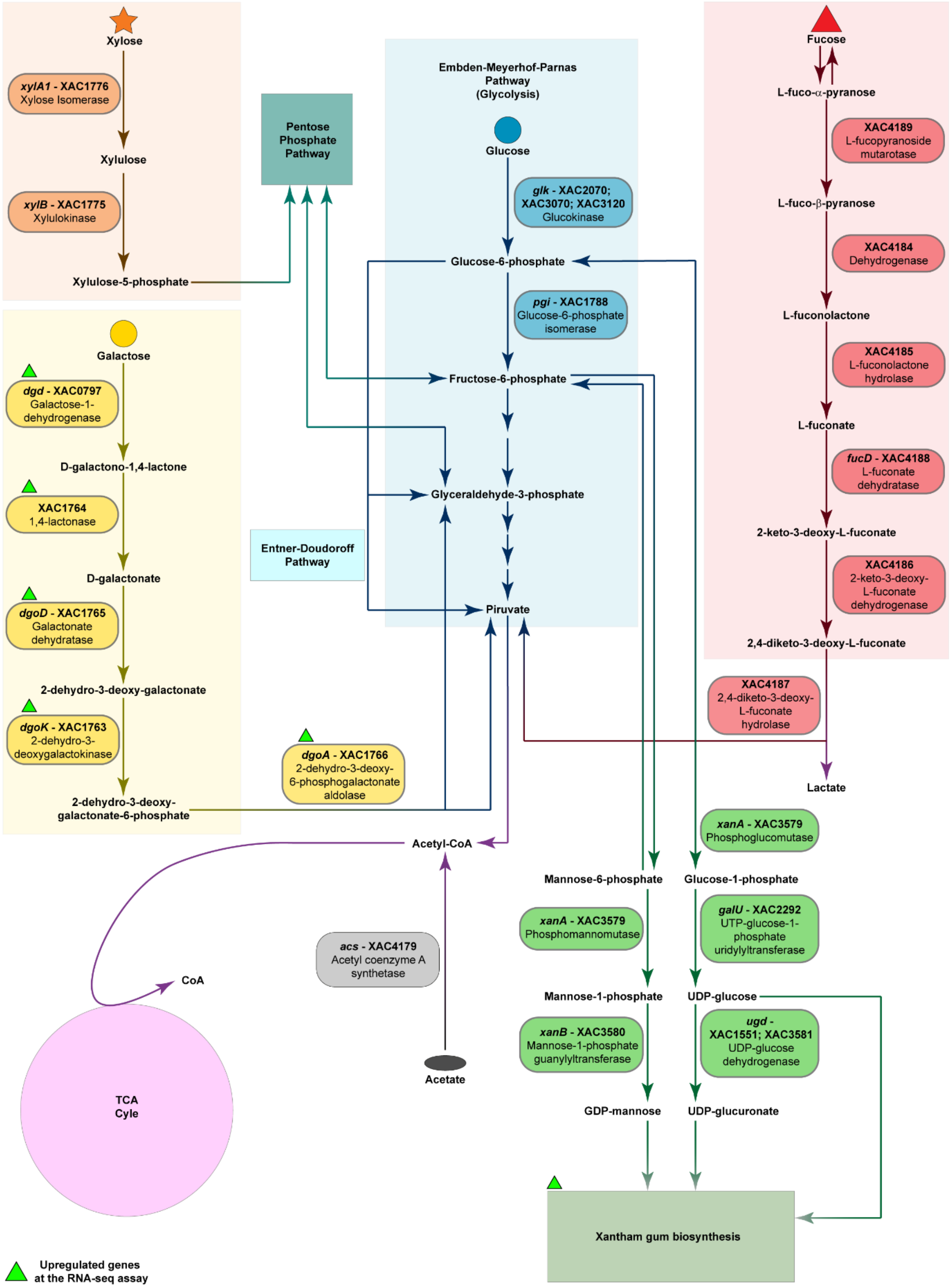

Supplementary Figure 28. Schematic representation of *X. citri* metabolism for XyG-released products. In the *X. citri* cytoplasm, monosaccharides released from XyG can be metabolized by specific pathways (boxes colored in light shades of orange, red, blue and yellow) that converge to the pentose phosphate pathway or to the tricarboxylic acid (TCA) cycle. Intermediate metabolites from glycolysis can also be converted into precursors for xanthan gum biosynthesis. The acetate released by the xyloglucan acetylesterase *Xac*XaeA is possibly imported to the cytoplasm through the cation/acetate symporter *ppA* (XAC4176) and then converted in acetyl-CoA to enter the TCA cycle. The reconstruction of *X. citri* main reactions of the central metabolism was based on previous works with *X. campestris* pv. *campestris* B100[20–23] and *X. campestris* pv. *campestris* ATCC 33913[24]. Arrows represent enzyme-catalyzed metabolic interconversions. Rounded corner rectangles highlight the genes and respective enzymes associated with peripheral reactions. Co-factors were omitted to improve clarity. Carbohydrates and acetate are represented by geometric shapes (glucose: blue circle, xylose: orange star, fucose: red triangle, galactose: yellow circle and acetate: gray ellipse). Green triangles indicate genes upregulated in the XyGOs condition, as identified at the RNA-seq assay presented in this work.

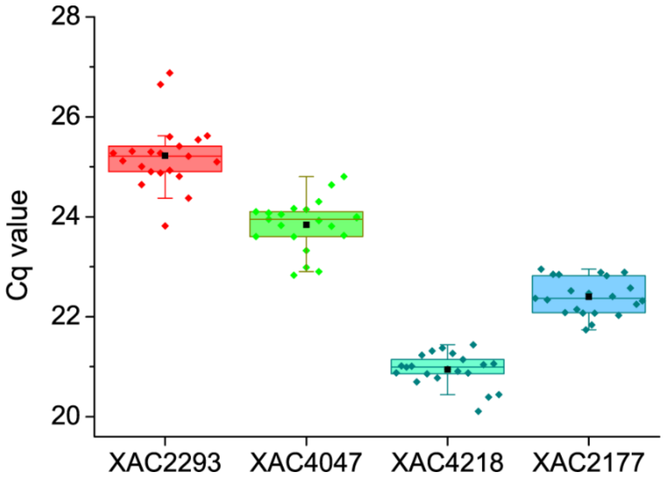

Supplementary Figure 29. Variation in the expression of candidate reference genes. Each box plot of Cq data (dots) presents the median (horizontal line inside the box), the mean value (black square), the first (Q_1) and third (Q_3) quartiles (lower and upper edges of the box), and whiskers (vertical lines) extending to the most extreme values inside lower [$Q_1 - 1.5(Q_3\text{-}Q_1)$] and upper [$Q_3 + 1.5(Q_3\text{-}Q_1)$] fences. Dots outside the whiskers are considered outliers. The variation in the expression levels was evaluated in seven different culture conditions for each gene. Statistics are derived from three independent experiments with technical duplicates. Cq: quantification cycle.

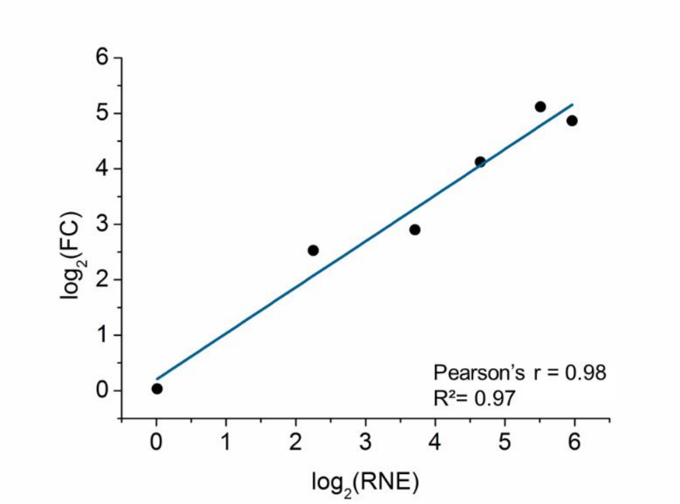

Supplementary Figure 30. Pearson's correlation between RNA-seq and RT-qPCR data. Log_2 (FC) values from RNA-seq assays and log_2 (RNE) values from RT-qPCR assays were analyzed for 6 genes (black dots) using Pearson´s correlation. r = Pearson correlation coefficient. R^2 = coefficient of determination (*p*-value <0.001, two-tailed). Blue line = linear regression fit. Gene expression data were obtained for *X. citri* growth in minimal medium XVM2m containing XyGOs in comparison to XVM2m glucose medium. FC: fold change and RNE: relative normalized expression.

Supplementary Table 1. Sequence-based prediction of subcellular localization of proteins from the *Xanthomonas* XyGUL and accessory enzymes for xyloglucan depolymerization. Of note, the position of the start codon originally annotated for these ORFs was revised based on the reference sequences whose accession codes are shown in NR ID. [a]SPII signal predicts for lipoproteins, *i.e* membrane-tethered proteins via N-terminal lipidation. [b]Position +2 different from D (aspartic acid) after a SPII cleavage site indicates outer-membrane anchoring[25]. TMH = transmembrane helix. CYT = cytoplasm, EXT = extracellular, IM = inner membrane, PER = periplasm, OM = outer membrane.

Enzymes encoded by the XyGUL

Gene	Protein	NR ID	Description	SignalP 5.0[26]	TMHMM[27]	CELLO v.2.5[28]	Consensus	Criterium
**XAC1770**	*Xac*Xeg74	WP_011051128.1	Endo-β-1,4-Xyloglucanase	Sec/SPI (1-38**)**	1 TMH (13-32)	EXT	EXT	SPI site accessible
**XAC1771**	*Xac*XaeA	WP_011051129.1	Xyloglucan Acetylesterase	Sec/SPI (1-23)	1 TMH (9-31)	PER	PER	SPI site accessible (region 24-31 is seen in the crystal structure and is not helical)
**XAC1772**	*Xac*GalD	WP_033485297.1	β-Galactosidase	Sec/SPII[a] (1-34) +2=Q	-	PER	OM	N-terminal lipid anchor / +2≠D[b]
**XAC1773**	*Xac*Xyl31	WP_011051131.1	α-Xylosidase	Sec/SPI (1-36)	-	PER	PER	SPI site accessible/PER
**XAC1774**	*Xac*Afc95	WP_011051132.1	α-L-1,2-Fucosidase	Tat/SPI (1-27)	-	PER	PER	SPI site accessible/PER

Putative accessory enzymes encoded outside the XyGUL

Gene	Protein	NR ID	Description	SignalP 5.0[26]	TMHMM[27]	CELLO v.2.5[28]	Consensus	Criterium
**XAC0029**	*Xac*Egl5B	WP_011050033.1	Endo-β-1,4-glucanase	Sec/SPI (29-30)	**-**	OM	EXT[29]	SPI site accessible and literature
**XAC1448**	*Xac*Bgl3A	WP_040107594.1	β-Glucosidase	Sec/SPI (1-16)	**-**	PER	PER	SPI site accessible/PER
**XAC1793**	*Xac*Bgl3B	WP_011051143.1	β-glucosidase	Sec/SPII[a] (1-30) +2=Q	-	PER	OM	N-terminal lipid anchor pos+2≠D[b]
**XAC2522**	*Xac*Egl9	WP_011051647.1	Endo-β-1,4-glucanase	Sec/SPII[¥] (1-19) +2=A	-	PER	OM	N-terminal lipid anchor pos+2≠D[b]
**XAC3869**	*Xac*Bgl3C	WP_011052578.1	β-Glucosidase	-	**-**	CYT	CYT	Lack of signal peptide
**XAC4183**	*Xac*Abf43A	WP_011052791.1	α-L-arabinofuranosidase	TAT/SPI (1-30)	-	PER	PER	SPI site accessible/PER

Supplementary Table 2. BLAST search using *Xac*XaeA sequence as query against the Protein Data Bank database. Note the low sequence coverage along with the low sequence identity.

Description	PDB code	Sequence identity (%)	E-value	Sequence Coverage (%)
GH2 β -glucuronidase from *Bacteroides uniformis*	6D8G[30]	32.63	2e-05	14
GH2 β-Galactosidase from *Thermotoga maritma*	6SD0[31]	27.12	0.017	23
GH2 β-Galactosidase from *Bacillus circulans*	4YPJ[32]	28.57	0.072	15
GH2 β-Galactosidase from *Bacteroides vulgatus*	3GM8	24.03	0.23	17
GH2 β-Glucuronidase from *Ruminococcus gnavus*	6JZ1	30.14	0.27	11
CE6 member from *Arabidopsis thaliana*	2APJ[10]	26.05	0.35	17

Supplementary Table 3. List of *Xanthomonas* species and information about the disease and tissue/host specificity.

Specie	Host	Disease	Local of infection	Reference
X. *campestris* pv. *campestris* 3811	Brassicaceae; *Arabidopsis thaliana*	Black rot	Vascular	[33,34]
X. campestris pv. *raphani* 756C	Brassicaceae; *Arabidopsis thaliana*	Bacterial spot	Non-vascular	[35,36]
X. vesicatoria ATCC 35937 LMG911	Tomato and pepper	Bacterial spot	Non-vascular	[37–39]
X. vesicatoria LM159	Tomato and pepper	Bacterial spot	Non-vascular	[37–39]
X. cucurbitae ATCC 23378	Pumpkin	Bacterial spot	Non-vascular	[39–41]
X. campestris pv. *badrii* NEB122	*Xanthium strumarium, Pisum sativum*	Unknown	Unknown	[42]
X. hortorum B07-007	Lettuce (*Lactuca sativa*)	Bacterial spot	Unknown	[43]
X. arboricola 17	Unknown	Unknown	Unknown	-
X. arboricola pv. *pruni* 15-088	*Prunus* species	Bacterial spot	Non-vascular	[44,45]
X. arboricola pv. *juglandis*	Walnut	Walnut blight	Non-vascular	[45]
X. sp. CPBF 424	Walnut	Walnut blight	Unknown	[46]
X. fragariae	Strawberry	Bacterial angular spot	Non-vascular	[39,47,48]
X. axonopodis pv. *commiphoreae* LMG26789	Guggal (*Commiphora wightii*)	Gumming of Guggal	Unknown	[49]
X. axonopodis pv. *citrumelo* F1	*Citrus* species	Bacterial spot	Non-vascular	[50,51]
X. *campestris* pv. *vesicatoria* str. 85-10	Pepper	Bacterial spot	Non-vascular	[52]
X. euvesicatoria LMG930	Tomato	Bacterial spot	Non-vascular	[37–39]
X. perforans 91-118	Tomato	Bacterial spot	Non-vascular	[39,53]
X. phaseoli pv. *phaseoli* CFBP412	Common bean (*Phaseolus vulgaris*)	Bacterial blight	Vascular	[54]
X. citri pv. *fuscans* 4834-R	Common bean (*Phaseolus vulgaris*)	Bacterial blight	Vascular	[55]
X. citri pv. *phaseoli* var. *fuscans* CFBP4885*	Common bean (*Phaseolus vulgaris*)	Bacterial blight	Vascular	[55,56]
X. citri pv. *vignicola* CFBP7113	Cowpea (*Vigna unguiculata*)	Bacterial blight	Non-vascular	[57,58]
X. citri pv. *aurantifolii* 1566	*Citrus* species	Citrus Canker	Non-vascular	[39,59]
X. citri pv. *citri* Aw12879	*Citrus* species	Citrus Canker	Non-vascular	[39,60]
X. axonopodis Xac29-1	*Citrus* species	Citrus Canker	Non-vascular	[39,61]
X. citri pv. *citri* 306	*Citrus* species	Citrus Canker	Non-vascular	[62,63]

Supplementary Table 3. Continued.

Specie	Host	Disease	Local of infection	Reference
X. citri pv. *malvacearum* AR81009	Cotton (*Gossypium hirsutum*)	Bacterial blight	Non-vascular	[64,65]
X. citri pv. *punicae* LMG7439	Pomegranate (*Punica granatum L.*)	Bacterial blight	Non-vascular	[66,67]
X. citri pv. *glycines* strain 12-2	Soybean (*Glycine max*)	Bacterial pustule	Non-vascular	[68]
X. axonopodis pv. *vasculorum* NCPPB 796	Sugarcane, Corn	Gumming disease/Bacterial leaf streak	Unknown	[69,70]
X. vasicola NCPPB 1060	Sorghum (Sorghum vulgare)	Bacterial leaf streak	Unknown	[39,70]
X.vasicola pv. *vasculorum* Xv1601	Corn (*Zea mays*)	Bacterial leaf streak	Unknown	[39,71]
X. campestris pv. *musacearum* NCPPB4379	Banana (*Musa* species)	Enset wilt	Vascular	[72]
X. vasicola NCPPB 902	*Tripsacum laxum*	Unknown	Unknown	[70]
X. vasicola pv. *arecae* NCPPB 2649	Betel palm (*Areca catechu*)	Unknown	Unknown	[70]
X. oryzae BAI23	Weed	Unknown	Unknown	[73,74]
X. oryzae pv. *oryzae* PXO99A	Rice (*Oryza sativa L.*)	Bacterial blight	Vascular	[39,75]
X. oryzae pv. *oryzicola* BLS256	Rice (*Oryza sativa L.*)	Bacterial leaf streak	Non-vascular	[75,76]
X.translucens pv. *translucens* DSM 18974	Barley (*Hordeum vulgare*)	Black chaff	Vascular	[39,77,78]
X. translucens pv. *undulosa* P3	Wheat (*Triticum* sp.)	Black chaff	Non-vascular	[78–80]
Xanthomonas hyacinthi CFBP 1156	Jacinth (*Hyacinthus*)	Yellow disease	Non-vascular	[39,81]
X. sacchari R1	Isolated from rice seeds	Nonpathogenic		[82,83]
X. albilineans Xa-FJ1	Sugarcane	Leaf scald	Vascular	[84]
X. albilineans GPE PC73	Sugarcane	Leaf scald	Vascular	[84]

Supplementary Table 4. Data collection and data refinement statistics for crystallographic data. Values in parenthesis represent the higher resolution shell. Carbohydrate validations were performed by Privateer software[85] (see details in Supplementary Table 5).

	***Xcc*Xeg74 (GH74)**	***Xac*XaeA (CE20)**	***Xac*GalD (GH35)**	***Xac*GalD (GH35)**
**Ligand**	XG	-	-	Galactose
**PDB Code**	7KN8	7KMM	7KMN	7KMO
**Data collection**				
Space group	P 21 21 21	P 1 21 1	I 4	I 4
Cell dimensions				
a, *b*, *c* (Å)	81.79, 100.43, 168.27	77.85, 63.50, 88.28	115.82, 115.82, 97.22	116.68, 116.68, 97.77
α, β, γ (°)	90.00	90.00, 98.15, 90.00	90.00	90.00
Resolution (Å)	38.80 - 1.95 (2.07 - 1.95)	87.39 - 1.90 (2.01 - 1.90)	19.86 - 1.80 (1.91 - 1.80)	46.03 - 1.75 (1.85 - 1.75)
R_{meas}	0.20 (1.09)	0.14 (1.55)	0.11 (2.13)	0.16 (2.94)
$I / \sigma(I)$	11.02 (2.50)	12.01 (1.36)	12.80 (0.85)	8.92 (0.64)
$CC_{1/2}$ (%)	99.7 (88.8)	99.8 (61.7)	99.9 (37.6)	99.8 (32.7)
Completeness (%)	98.1 (93.7)	95.6 (94.6)	100.0 (99.9)	97.4 (99.8)
Redundancy	12.19 (10.12)	7.13 (7.04)	6.58 (6.07)	6.47 (5.96)
**Refinement**				
Resolution (Å)	19.99 - 1.95	19.97 - 1.90	19.86 - 1.80	19.45 - 1.75
No. reflections	99535	62206	59288	60752
R_{work} / R_{free}	0.216/0.252	0.190/0.238	0.177/0.210	0.193/0.242
No. atoms				
Protein	10852	6607	4040	4040
Ligand/ion	100/16	0/13	0/20	42/10
Water	1084	381	520	506
B-factors ($Å^2$)				
Protein	19.29	26.43	31.47	37.10
Ligand/ion	20.56/35.47	0/54.02	0/62.94	46.68/100.25
Water	20.28	28.59	36.83	41.88
R.m.s. deviations				
Bond lengths (Å)	0.007	0.007	0.007	0.007
Bond angles (°)	0.876	0.88	0.880	0.896
Carbohydrate validation				
Stereochemical problems	0	-	-	0
Unphysical puckering	0	-	-	0
Unlikely conformations	0	-	-	0

Supplementary Table 4. Continued.

	***Xac*Xyl31 (GH31)**	***Xac*Xyl31 (GH31)**	***Xac*Afc95 (GH95)**
**Ligand**	-	Xylose	-
**PDB Code**	7KMP	7KNC	7KMQ
**Data collection**			
Space group	P 21 21 2	P 21 21 2	P 21 21 21
Cell dimensions			
a, *b*, *c* (Å)	117.10, 145.69, 62.87	117.49, 146.63, 63.04	103.91, 103.91, 173.75
α, β, γ (°)	90.00	90.00	90.00
Resolution (Å)	45.81 - 1.56 (1.64 - 1.56)	38.63 - 1.86 (1.97 - 1.86)	20.00 – 2.05 (2.17 – 2.05)
R_{meas}	0.07 (0.28)	0.09 (0.35)	0.08 (1.05)
I / σ(I)	20.15 (6.96)	17.65 (6.66)	13.15 (1.31)
$CC_{1/2}$ (%)	99.9 (97.9)	99.8 (96.2)	99.8 (46.5)
Completeness (%)	99.7 (98.7)	98.3 (91.2)	96.8 (96.1)
Redundancy	9.71 (9.64)	9.70 (9.41)	3.50 (3.22)
**Refinement**			
Resolution (Å)	45.81 - 1.56	38.63 - 1.87	19.78 – 2.05
No. reflections	155561	88980	117432
R_{work} / R_{free}	0.163/0.185	0.191/0.225	0.175/0.191
No. atoms			
Protein	7414	7348	11750
Ligand/ion	78/1	16/1	36/2
Water	1132	797	202
B-factors (Å^2)			
Protein	20.09	24.42	37.90
Ligand/ion	30.43/25.55	31.04/29.52	40.79/45.24
Water	31.73	30.17	36.27
R.m.s. deviations			
Bond lengths (Å)	0.007	0.004	0.002
Bond angles (°)	0.850	0.713	0.591
Carbohydrate validation			
Stereochemical problems	-	0	-
Unphysical puckering	-	0	-
Unlikely conformations	-	0	-

Supplementary Table 5. Privateer[85] analysis of the carbohydrates complexed in the crystallographic structures. Detailed monosaccharide data for *Xcc*Xeg74 (7KN8), *Xac*GalD (7KMO) and *Xac*Xyl31 (7KNC) carbohydrates complexes (Supplementary Table 4). The chain identifiers represent each ligand as defined in the respective PDB file. Names refer to the three-letter code correspondent to the monosaccharide in the model: BGC = β-glucose, XYS = α-xylose, GLA = α-galactose. The puckering amplitude Q is represented in Angstroms, and the angles φ and θ are represented in degrees. Puckering parameters were used to determine the conformations for pyranoses, as defined by Cremer-Pople[86]. Anomer is the α/β geometric variation of the epimer, considering the anomeric carbon in the pyranoses. D/L is the isomeric form of the carbohydrate handedness found in the structure (D = right-handed, L = left-handed, N = unable to determine based solely in the structure). Conformation refers to the overall tridimensional structure adopted by the monosaccharide. RSCC is short for Real Space Correlation Coefficient, which measures the agreement between model and positive omit density. A RSCC below 0.8 is typically considered poor. *B*-factor column is the corresponding value for the monosaccharide, considering the overall crystallographic structure. Finally, the Diagnostic column indicates whether the monosaccharide is in a stereochemical and conformationally acceptable configuration, as evaluated by Privateer.

***Xcc*Xeg74 complexed with xyloglucan oligosaccharide (7KN8)**										
**Chain**	**Name**	**Q**	**φ**	**θ**	**Conformation**	**Anomer**	**D/L**	**RSCC**	**<*B*-factor>**	**Diagnostic**
C	BGC	0.551	50.2387	15.6044	$^4C_1$	β	D	0.80	22.4058	OK
C	BGC	0.535	224.838	2.65641	$^4C_1$	β	D	0.89	19.0155	OK
C	XYS	0.530	109.429	7.7146	$^4C_1$	α	N	0.84	22.1111	OK
D	BGC	0.529	161.316	7.70464	$^4C_1$	β	D	0.84	21.8817	OK
D	BGC	0.552	269.654	4.8517	$^4C_1$	β	D	0.89	19.2345	OK
D	XYS	0.550	89.8472	12.1197	$^4C_1$	α	N	0.81	21.6567	OK
***Xac*GalD complexed with galactose (7KMO)**										
**Chain**	**Name**	**Q**	**φ**	**θ**	**Conformation**	**Anomer**	**D/L**	**RSCC**	**<*B*-factor>**	**Diagnostic**
C	GLA	0.552	261.192	8.60379	$^4C_1$	α	D	0.79	35.3717	Ok
***Xac*Xyl31 complexed with xylose (7KNC)**										
**Chain**	**Name**	**Q**	**φ**	**θ**	**Conformation**	**Anomer**	**D/L**	**RSCC**	**<*B*-factor>**	**Diagnostic**
C	XYS	0.546	121.95	8.0779	$^4C_1$	α	N	0.82	27.137	Ok

Supplementary Table 6. List of synthetic substrates used for activity detection screening of XyGUL and accessory enzymes. N.d. - activity not detected for the enzyme on the substrate. The values are qualitative and describe the relative activity in percentage. 100 µL of 1 mol.L^{-1} sodium tetraborate was added to stop the enzymatic reaction and the released *para*-nitrophenol (pNP) was measured at 400 nm using an Infinite® 200 PRO microplate reader (Tecan).

**Substrates**	**Enzymes**										
	**GH74**	**CE20**	**GH35**	**GH31**	**GH95**	**GH3**	**GH3**	**GH3**	**GH43**	**GH3**	**GH3**
	***Xac*Xeg74**	***Xac*XaeA**	***Xac*GalD**	***Xac*Xyl31**	***Xac*Afc95**	***Xac*Bgl3A**	***Xac*Bgl3B**	***Xac*Bgl3C**	***Xac*Abf43A**	***Xac*Xyl3A**	***Xac*Xyl3B**
pNP-α-D-galactopyranoside	n.d.	n.d.	n.d.	n.d.	n.d.	n.d.	n.d.	n.d.	n.d.	n.d.	n.d.
pNP-α-D-glucopyranoside	n.d.	n.d.	n.d.	n.d.	n.d.	n.d.	n.d.	n.d.	n.d.	n.d.	n.d.
pNP-α-D-mannopyranoside	n.d.	n.d.	n.d.	n.d.	n.d.	n.d.	n.d.	n.d.	n.d.	n.d.	n.d.
pNP -α-D-xylopyranoside	n.d.	n.d.	n.d.	100	n.d.	n.d.	n.d.	n.d.	n.d.	n.d.	n.d.
pNP-α-L-arabinofuranoside	n.d.	n.d.	n.d.	n.d.	n.d.	n.d.	n.d.	n.d.	100	n.d.	20
pNP-α-L-arabinopyranoside	n.d.	n.d.	87	n.d.	n.d.	n.d.	n.d.	n.d.	n.d.	n.d.	n.d.
pNP-α-L-fucopyranoside	n.d.	n.d.	n.d.	n.d.	100	n.d.	n.d.	n.d.	n.d.	n.d.	n.d.
pNP-α-L-rhamnopyranoside	n.d.	n.d.	n.d.	n.d.	n.d.	n.d.	n.d.	n.d.	n.d.	n.d.	n.d.
pNP-β-D-cellobioside	n.d.	n.d.	n.d.	n.d.	n.d.	2	4	n.d.	n.d.	n.d.	n.d.
pNP-β-D-fucopyranoside	n.d.	n.d.	100	n.d.	n.d.	n.d.	n.d.	n.d.	n.d.	n.d.	n.d.
pNP-β-D-galactopyranoside	n.d.	n.d.	83	n.d.	n.d.	n.d.	n.d.	n.d.	n.d.	n.d.	n.d.
pNP-β-D-glucopyranoside	n.d.	n.d.	n.d.	n.d.	n.d.	100	100	100	n.d.	n.d.	6
pNP-β-D-mannopyranoside	n.d.	n.d.	n.d.	n.d.	n.d.	n.d.	n.d.	n.d.	n.d.	n.d.	n.d.
pNP-β-D-xylopyranoside	n.d.	n.d.	n.d.	n.d.	n.d.	n.d.	n.d.	n.d.	10	100	100
pNP-metanoate	-	19	-	-	-	-	-	-	-	-	-
pNP-acetate	-	100	-	-	-	-	-	-	-	-	-
pNP-butirate	-	n.d.	-	-	-	-	-	-	-	-	-
pNP-pentanoate	-	n.d.	-	-	-	-	-	-	-	-	-
pNP-octanoate	-	n.d.	-	-	-	-	-	-	-	-	-
pNP-decanoate	-	n.d.	-	-	-	-	-	-	-	-	-
pNP-dodecanoate	-	n.d.	-	-	-	-	-	-	-	-	-
pNP-tetradecanoate	-	n.d.	-	-	-	-	-	-	-	-	-
pNP-hexadecanoate	-	n.d.	-	-	-	-	-	-	-	-	-

Supplementary Table 7. List of polymeric substrates used for activity detection screening of XyGUL and accessory enzymes. N.d. - activity not detected for the enzyme on the substrate. The values are qualitative and describe the relative activity in percentage. The amount of reducing sugar released from polysaccharides was assessed by the 3,5-dinitrosalicylic acid method[87] and measured at 540 nm using an Infinite® 200 PRO microplate reader (Tecan).

**Substrates**	**Enzymes**								
	**GH74**	**CE20**	**GH35**	**GH31**	**GH95**	**GH3**	**GH3**	**GH3**	**GH43**
	***Xac*Xeg74**	***Xac*XaeA**	***Xac*GalD**	***Xac*Xyl31**	***Xac*Afc95**	***Xac*Bgl3A**	***Xac*Bgl3B**	***Xac*Bgl3C**	***Xac*Abf43A**
Arabinan	n.d.	n.d.	n.d.	n.d.	n.d.	n.d.	n.d.	n.d.	n.d.
Arabinogalactan	n.d.	n.d.	n.d.	n.d.	n.d.	n.d.	n.d.	n.d.	n.d.
Arabinoxylan	n.d.	n.d.	n.d.	n.d.	n.d.	n.d.	n.d.	n.d.	n.d.
Avicel PH-101	20	n.d.	n.d.	n.d.	n.d.	n.d.	n.d.	n.d.	n.d.
β-1,4-glucobiose	n.d.	n.d.	n.d.	n.d.	n.d.	91	100	100	-
CM-cellulose	18.	n.d.	n.d.	n.d.	n.d.	n.d.	n.d.	n.d.	n.d.
CM-curdlan	n.d.	n.d.	n.d.	n.d.	n.d.	n.d.	n.d.	n.d.	n.d.
Curdlan	n.d.	n.d.	n.d.	n.d.	n.d.	n.d.	n.d.	n.d.	n.d.
Galactan	n.d.	n.d.	n.d.	n.d.	n.d.	n.d.	n.d.	n.d.	n.d.
Galactomannan	n.d.	n.d.	n.d.	n.d.	n.d.	n.d.	n.d.	n.d.	n.d.
Polygalacturonic acid	n.d.	n.d.	n.d.	n.d.	n.d.	n.d.	n.d.	n.d.	n.d.
Glucomannan	n.d.	n.d.	n.d.	n.d.	n.d.	n.d.	n.d.	n.d.	n.d.
Galactan	n.d.	n.d.	n.d.	n.d.	n.d.	n.d.	n.d.	n.d.	n.d.
Laminarin	n.d.	n.d.	n.d.	n.d.	n.d.	100	20	5	n.d.
Lichenan	n.d.	n.d.	n.d.	n.d.	n.d.	n.d.	n.d.	n.d.	n.d.
Mannan	n.d.	n.d.	n.d.	n.d.	n.d.	n.d.	n.d.	n.d.	n.d.
Pachyman	n.d.	n.d.	n.d.	n.d.	n.d.	n.d.	n.d.	n.d.	n.d.
CM-pachyman	n.d.	n.d.	n.d.	n.d.	n.d.	n.d.	n.d.	n.d.	n.d.
Pectin	n.d.	n.d.	n.d.	n.d.	n.d.	n.d.	n.d.	n.d.	n.d.
Pululan	n.d.	n.d.	n.d.	n.d.	n.d.	n.d.	n.d.	n.d.	n.d.
Reduced pululan	n.d.	n.d.	n.d.	n.d.	n.d.	n.d.	n.d.	n.d.	n.d.
Rhamnogalacturan I	n.d.	n.d.	n.d.	n.d.	n.d.	n.d.	n.d.	n.d.	n.d.
Rhamnogalacturan	n.d.	n.d.	n.d.	n.d.	n.d.	n.d.	n.d.	n.d.	n.d.
Xanthan Gum	n.d.	n.d.	n.d.	n.d.	n.d.	n.d.	n.d.	n.d.	n.d.
Xylan	n.d.	n.d.	n.d.	n.d.	n.d.	n.d.	n.d.	n.d.	n.d.
Amyloid Xyloglucan	100	n.d.	100	100	n.d.	n.d.	n.d.	n.d.	n.d.
Xyloglucan	96	n.d.	74	50.	n.d.	n.d.	n.d.	n.d.	n.d.
β-glucan	n.d.	n.d.	n.d.	n.d.	n.d.	n.d.	n.d.	n.d.	n.d.

Supplementary Table 8. Biochemical parameters of XyGUL and accessory enzymes. Kinetic parameters were obtained in the preferred conditions. pNP = *para*-nitrophenyl, Gal*p* = galactopyranoside, Xyl*p* = xylopyranoside, Fuc*p* = fucopyranoside and Glu*p* = glucopyranoside.

Enzyme	Substrate	pH	T (°C)	$K_{0.5}$ (mmol.L^{-1} or mg.mL^{-1}*)	k_{cat} (s^{-1})	$k_{cat}/K_{0.5}$ (L.mmol^{-1}.s^{-1} or L.g^{-1}.s^{-1}*)
*Xac*Xeg74	Tamarind xyloglucan	6.0	35	3.34 ± 0.58*	4.40 ± 0.26	1.32*
*Xac*XaeA	pNP-acetate	7.5	20	0.66 ± 0. 05	1.59x10^{4} ± 244	2.41.10^{4}
*Xac*GalD	pNP-β-D-Gal*p*	5.0	50	1.06 ± 0.09	1.42x10^{6} ± 21807	1.34.10^{6}
*Xac*Xyl31	pNP-α-D-Xyl*p*	6.0	45	1.65 ± 0.19	1.29x10^{5} ± 3997	7.81.10^{4}
*Xac*Afc95	pNP-α-L-Fuc*p*	6.0	55	3.24 ± 0.37	3.13x10^{4} ± 1757	9.66.10^{3}
*Xac*Bgl3A	pNP-β-D-Glu*p*	5.5	45	5.23 ± 0.19	141.55 ± 4.42	27.07
	β-1,4-glucobiose			38.72 ± 24.03*	5.65 ± 1.32	0.15*
	Laminarin			2.30 ± 0.07*	3.01 ± 0.08	1.31*
*Xac*Bgl3B	pNP-β-D-Glu*p*	6.0	25	3.68 ± 0.25	32.83 ± 0.54	8.92
	β-1,4-glucobiose			1.35 ± 0.15*	8.16 ± 0.19	6.04*
	Laminarin			5.93 ± 0.20*	2.47 ± 0.06	0.42*
*Xac*Bgl3C	pNP-β-D-Glu*p*	7.5	35	3.36 ± 0.19	13.46 ± 0.32	4.01
	β-1,4-glucobiose			16.08 ± 1.10*	3.73 ± 0.09	0.23*
	Laminarin			11.39 ± 0.26*	1.73 ± 0.03	0.15*
*Xac*Egl9	Tamarind xyloglucan	6.0	35	4.82 ± 0.11*	4.04 ± 0.07	0.84*

Supplementary Table 9. Composition, structure and nominal masses of ions generated by ESI(+)-MS of xyloglucan oligosaccharides obtained from *Arabidopsis thaliana* cell wall, as depicted in the supplementary Fig. 8. Letters indicate the type of substitutions appended to the glucose backbone of the identified oligosaccharides. G = non-substituted glucose, X = glucose substituted with a xylose at C-6, L = X with a galactose appended at xylose C-2 and F = L with a fucose appended at galactose C-2. OAc indicates the *O*-acetyl substituent at galactose C-6. Hex = hexose, Pen = pentose and Dox = deoxyhexose. M = nominal mass.

Nominal mass	Composition	Suggested structure
1247	Hex_5Pen_3 $[M + Na]^+$	XXLG/XLXG
1289	$Hex_5Pen_3OAc_1$ $[M + Na]^+$	XXLG/XLXG + 1OAc
1393	$Hex_5Pen_3Dox_1$ $[M + Na]^+$	XXFG
1435	$Hex_5Pen_3Dox_1OAc_1$ $[M + Na]^+$	XXFG + 1OAc
1555	$Hex_6Pen_3Dox_1$ $[M + Na]^+$	XLFG
1597	$Hex_6Pen_3Dox_1OAc_1$ $[M + Na]^+$	XLFG + 1OAc

Supplementary Table 10. **SAXS analysis of XyGUL enzymes.** The molecular weight calculated from the data by SAXSMoW[88] server (MW_{MoW}) was compared to the predicted molecular weight derived from the amino acid sequence (MW_{AA}). Maximum diameter (D_{max}), radius of gyration (R_g), molecular volume (V_m) and Porod volume (V_P) were calculated by Primusqt software[89]. If it applies, oligomeric interface interaction energy was also calculated by PDBePISA server[90]. Negative values in this field represent favorable quaternary structure assemblies. *Xac*GalD was analyzed by SEC-MALS experiments and the results are not listed in this table (see Supplementary Fig. 17).

Enzyme	D_{max} (nm)	R_g (nm)	V_m (nm^3)	V_P (nm^3)	MW_{MoW} (kDa)	MW_{AA} (kDa)	Δ^iG (kcal.mol^{-1})
*Xac*Xeg74	9.00	3.16	98.20	97.11	81.02	79.44	-
*Xac*XaeA	10.60	3.28	87.32	92.09	72.04	68.48	-
*Xac*GalD	-	-	-	-	-	59.58	-4.4
*Xac*Xyl31	9.80	3.61	121.28	127.30	100.06	118.4	-
*Xac*Afc95	14.8	4.09	227.05	226.03	187.32	86.68	-5.7

Supplementary Table 11. GH35 structures reported in the CAZy database. The table lists at least one representative structure (when available) for each organism in the GH35 family (PDB entry column), sorted by Domain and Phylum. The quaternary structure types were determined using the PDBePISA server[90] (See Supplementary Fig. 20 for the examples highlighted in bold in the table), with each respective assembly solvation free energy gain presented as ΔG^{int} (kcal.mol^{-1}). A negative value in this column means a favorable calculated quaternary structure. The D562 residue from the other subunit that interacts with the galactose at the -1 subsite is indicated when conserved. The experimentally determined activity is also listed as annotated in the CAZy database.

Domain	Organism	PDB entry	Quaternary structure	ΔG^{int} (PISA)	Accessory domains	D562 conservation	Function	Reference
Archaea (Thermococci)	*Pyrococcus furiosus* DSM 3638	6JOW	Dimer (I)	-59.8	2	-	-	-
Archaea (Thermococci)	*Pyrococcus horikoshii* OT3	**5GSL**	Dimer (I)	-31.4	2	-	exo-β-glucosaminidase (chitosan)	-
Archaea (Thermococci)	*Thermococcus kodakarensis* KOD1	5GSM	Dimer (I)	-79	2	-	exo-β-glucosaminidase (chitosan)	-
Bacteria (Firmicutes)	*Bacillus circulans* ATCC 31382	**4MAD**	Dimer (II)	-2.7	2	-	β-galactosidase (β-1,3)	[15]
Bacteria (Firmicutes)	*Streptococcus pneumoniae* TIGR4	4E8D	Dimer (II)	-10.9	2	-	β-galactosidase (β-1,3)	[91]
Bacteria (Bacteroidetes)	*Bacteroides thetaiotaomicron* VPI-5482	**6EON**	Monomer (I)	-	3	-	-	[17]
Bacteria (Proteobacteria)	*Caulobacter vibrioides* CB15	3U7V	Tetramer	-26.7	1	D527	β-galactosidase	-
Bacteria (Proteobacteria)	*Cellvibrio japonicus* Ueda107	5JAW	Tetramer	-156.4	1	D550	β-galactosidase (Xyloglucan)	[92]
Bacteria (Proteobacteria)	*Xanthomonas citri* pv. citri 306	**7KMN**	Tetramer	-284.9	1	D562	β-galactosidase (Xyloglucan)	This work
Eurkaryota (Fungi)	*Aspergillus oryzae* RIB40	**4IUG**	Monomer (II)	-	4	-	β-galactosidase (lactose, β-1,3 and β-1,4)	[18]
Eurkaryota (Fungi)	*Penicillium* sp.	1TG7	Monomer (II)	-	4	-	β-galactosidase	[93]
Eurkaryota (Fungi)	*Trichoderma reesei*	3OG2	Monomer (II)	-	4	-	β-galactosidase	[94]
Eurkaryota (Viridiplantae)	*Solanum lycopersicum*	3W5F	Monomer (II)	-	4	-	β-galactosidase (β-1,3 and β-1,4, pectin)	-
Eurkaryota (Metazoa)	*Homo sapiens*	**3THC**	Dimer (III)	-41.4	2	-	β-galactosidase	[16]

Supplementary Table 12. Expression profile of GH3 β-glucosidase genes from *X. citri* in RNA-seq assays. Data are shown as mean values of TPM ± SD from four independent biological replicates. TPM: Transcripts Per Million reads.

Locus	**Protein**	**TPM ± SD**
XAC1448	*Xac*Bgl3A	2.8 ±0.8
XAC1793	*Xac*Bgl3B	15.3 ± 4.6
XAC3869	*Xac*Bgl3C	35.5 ± 19.4

Supplementary Table 13. Differentially expressed genes in RNA-seq assays corresponding to the type III effector proteins (T3E) from *X. citri* pv. *citri* 306. Genes were considered differentially expressed according to the Wald test implemented in the DESeq2 package. *p*-values were adjusted for multiple tests using Benjamini-Hochberg (BH) method also implemented in DESeq2 package. Thresholds: $|\log_2$ fold change$| \geq 1$ and p adjusted ≤ 0.05.The overview of T3E can be assessed in *Xanthomonas* Resource (http://www.xanthomonas.org/t3e.html).

Locus	**$\log_2$ fold change**	***p*-adjusted**	**Description**
XAC0277	3.03	1.75E-17	XopR
XAC0286	3.59	2.41E-34	XopE1
XAC0754	2.76	5.74E-11	XopI
XAC1171	3.81	4.65E-19	XopAU
XAC1172	3.36	3.00E-19	XopAV
XAC1208	1.64	4.76E-02	XopP
XAC2009	2.23	3.68E-07	XopZ1
XAC2786	2.70	1.37E-16	XopN
XAC2922	3.67	2.28E-28	HrpW
XAC3085	2.67	2.77E-09	XopK
XAC3090	2.95	2.03E-13	XopL
XAC3666	2.54	1.18E-10	XopAK
XAC4213	2.11	4.65E-19	XopAD
XAC4333	3.03	4.17E-11	XopQ

Supplementary Table 14. Putative major facilitator superfamily (MFS) transporters from *X. citri* pv. *citri* 306. Corresponding genes were identified by search in the Pfam database (https://pfam.xfam.org/).

Locus	**Gene**	**Description**
XAC0110	*proP*	proline-betaine transporter
XAC0229		MFS transporter
XAC0303	*opdE*	transcriptional regulator
XAC0317	*ynfM*	MFS transporter
XAC0349	*vanK*	MFS transporter
XAC0507	*aas*	2-Acylglycerophosphoethanolamine acyltransferase
XAC0509		MFS transporter
XAC0642	*rmrB*	MFS transporter
XAC0712	*gluP*	glucose-galactose transporter
XAC1215	*bcr*	MFS transporter
XAC1363	*araJ*	MFS transporter
XAC1446	*pmrB*	multidrug resistance membrane translocase
XAC1450	*ygdR*	oligopeptide transporter
XAC1556	*fucP*	glucose-galactose transporter
XAC1705		MFS transporter
XAC1777	*xylE*	MFS transporter
XAC1801	*proP*	Prop transport protein
XAC2161	*tetV*	MFS transporter
XAC2234	*cynX*	MFS transporter
XAC2340	*cynX*	MFS transporter
XAC2356	*tetV*	drug/proton antiporter
XAC2474	*rmrB*	transport protein
XAC2484	*yhjE*	metabolite transport protein
XAC2488	*yhjX*	integral membrane transporter
XAC2494	*yieO*	drug resistance translocase
XAC2597	*suc1*	transport protein
XAC2837	*araJ*	MFS transporter
XAC3001	*ptr*	MFS transporter
XAC3027	*emrA*	MFS transporter

Supplementary Table 14. (Continued).Putative major facilitator superfamily (MFS) transporters from *X. citri* pv. *citri* 306. Corresponding genes were identified by search in the Pfam database (https://pfam.xfam.org/).

Locus	**Gene**	**Description**
XAC3056		conserved hypothetical protein
XAC3157	*ycaD*	transmembrane transport protein
XAC3179	*yceE*	transport protein
XAC3474	*cit1*	citrate carrier protein
XAC3488	*suc1*	sugar transporter
XAC3901	*ampG*	signal transducer
XAC4190	*fucP*	fucose permease
XAC4196	*ynaJ*	cation symporter
XAC4255	*exuT*	hexuranate transporter
XAC4295	*tetA*	tetracycline-efflux transporter
XAC4308	*kgtP*	dicarboxylate transport protein
XAC4361	*ttuB*	MFS transporter

Supplementary Table 15. Specific activity of endoglucanases from *X. citri* on XyG. Tamarind xyloglucan (2.5 $mg.mL^{-1}$) was used for the assay in conditions of pH and temperature detailed in the Supplementary Table 21. All constructs lack the N-terminal signal peptide. [a] catalytic domain. [b] Deletion of 27 residues of high hydrophobicity at C-terminus. N.D. = not detected. [c] [95] (V_0=initial velocity and $[E]_t$=enzyme concentration)

Gene	Protein	Construct (a.a.)	Family	$V_0/[E]_t$ (s^{-1})
XAC2522	*Xac*Egl9	(22-586)	GH9	1.00 ± 0.03
XAC0029	*Xac*Egl5B	(27-350)	GH5_5	0.34 ± 0.03
XAC0612	*Xac*EngXCA	(26-368)[a]	GH5_1	0.10 ± 0.07
XAC0030	*Xac*Egl5C	(37-357)	GH5_5	N.D.
XAC0028	*Xac*Egl5A	(31-350)[b]	GH5_5	N.D.
XAC0346	-	(53-453)	GH5	N.D.
XAC3516	*Xac*Cel8	(26-384)	GH8	N.D.[c]

Supplementary Table 16. List of primers and vectors used in gene cloning for the heterologous expression of XyGUL and accessory enzymes. Restriction sites are underlined. pET28a-XAC0346, pET28a-XAC1275 and pET28a-XAC3869 clones were purchased from GenScript.

Protein	Vector	Primer sequence (5'→3')	Restriction site
*Xac*Bgl3A	pET28a	F: GGAATTC<u>CATATG</u>CAGGGCGCGCCATCTTCGC	NdeI
		R: CCG<u>CTCGAG</u>TTACGGCAACTGTGCCGC	XhoI
*Xcc*Xeg74	pETM11	F: CAGGGCGCCATGGCCACGTCCGGGC	-
		R: GACCCGACGCGGTTATCTCGGATCGCCGTAG	-
*Xac*Xeg74	pET28a	F: <u>CATATG</u>GATGAGCCAGGTACGCCA	NdeI
		R: <u>AAGCTT</u>TTATCGTGGATCGCCATAGAAGAT	HindIII
*Xac*XaeA	pET28a	F: <u>CATATG</u>GTGCCAACACTACCGCTG	NdeI
		R: <u>AAGCTT</u>TTACCAGGTATCGGTGCG	HindIII
*Xac*GalD	pET28a	F: <u>CATATG</u>CAGACGCCGATGCCGCA	NdeI
		R: <u>AAGCTT</u>TTATTTGTAGGTTGCCAGTTTGATCTTGAGCAG	HindIII
*Xac*Xyl31	pET28a	F: <u>CATATG</u>CAGGAAGTGCGCAAGGC	NdeI
		R: <u>CTCGAG</u>TACGCTTTGCCCGACGCAATC	XhoI
*Xac*Afc95	pET28a	F: GAATTC<u>CATATG</u>CAGGCAACGCAGGCTTCGAA	NdeI
		R: <u>GTCGAC</u>TTATTGCGTCACCAATCGGT	SalI
*Xac*Bgl3B	pET28a	F: GCGGCCGCA<u>CATATG</u>GGCAAGGACACTGCTGCC	NdeI
		R: <u>CTCGAG</u>TTACTTGACAGGGCAGTCGACGGT	XhoI
*Xac*Egl9	pET28a	F: <u>CATATG</u>GCCGAGACGCCTGGCAGG	NdeI
		R: <u>CTCGAG</u>TTAGCGAGTCGACGCCTCGAT	XhoI
*Xac*Abf43A	pET28a	F: ATATAT<u>GCTAGC</u>ATGGCGACGCAGCGCGCGGG	NheI
		R: TATATA<u>GGATCC</u>TTACGTCAGCGCGCGATAGC	BamHI
*Xac*Egl5A	pET28a	F: <u>CATATG</u>ACGCGCGCGCTCAC	NdeI
		R: <u>CTCGAG</u>TTAACGTGTGGCGAGTGTG	XhoI
*Xac*Egl5B	pET28a	F: <u>CATATG</u>CAAAGCGCCACCGGCCTGAAG	NdeI
		R: <u>CTCGAG</u>TTAATCGGTAATCCGGCGCGCA	XhoI
*Xac*Egl5C	pET28a	F: <u>CATATG</u>CTGAAGTATGTTGGCGTCAATC	NdeI
		R: <u>GTCGAC</u>TTATGCGTACTTGCTAGGATC	SalI
*Xac*EngXCA	pET28a	F: <u>CATATG</u>ATGTATTCGGTCAGCAATAA	NdeI
		R: <u>AAGCTT</u>TCACCACAGCGTCCGCAA	HindIII

Supplementary Table 17. List of primers used for site-directed mutagenesis of XyGUL enzymes.

Protein	Residue change	Primer sequence (5'→3')
*Xac*GalD	D562A	F: CCGTAATCGGTCTGGGCGCCATTCCAGTTGC
		R: GCAACTGGAATGGCGCCCAGACCGATTACGG
	S106R	F: CCGGGATAGTTGCTGCGGTTGTTGACCTGCGC
		R: GCGCAGGTCAACAACCGCAGCAACTATCCCGG
*Xac*Xyl31	W328A	F: TGATTCCACGGATTCGCGTTCTGGCGCCAGCG
		R: CGCTGGCGCCAGAACGCGAATCCGTGGAATCA
*Xac*Afc95	T395H	F: CTCGGTGTTGATGTTGATGTGGTACTTGCTTTCCCACGGC
		R: GCCGTGGGAAAGCAAGTACCACATCAACATCAACACCGAG

Supplementary Table 18. Protein expression conditions.

Protein	Strain	Temperature	Time	Medium	[IPTG]
XAC1275	BL21 (DE3) pRARE II	18 °C	16 h	Auto-induction	-
*Xac*Bgl3A	BL21(DE3)	20°C	20 h	TB	0.5 mM
*Xcc*Xeg74	Rosetta 2 (DE3) pLysS	20°C	25 h	Auto-induction	-
*Xac*Xeg74	BL21(DE3) pLysS	20°C	16 h	LB	0.5 mM
*Xac*XaeA	BL21(DE3) pLysS	30 °C	4 h	LB	0.5 mM
*Xac*GalD	BL21(DE3) pLysS	30 °C	4h	LB	0.5 mM
*Xac*Xyl31	BL21(DE3) pLysS	20°C	16 h	LB	0.5 mM
*Xac*Afc95	BL21(DE3) pLysS	20°C	16 h	LB	0.5 mM
*Xac*Bgl3B	BL21(DE3)	20°C	20 h	TB	0.5 mM
*Xac*Egl9	BL21(DE3)	20°C	16 h	TB	0.2 mM
*Xac*Bgl3C	BL21(DE3)	20°C	20 h	TB	0.5 mM
*Xac*Abf43A	BL21(DE3)	18 °C	16 h	TB	0.5 mM
*Xac*Egl5A	BL21(DE3) SHuffle	20 °C	16 h	TB	0.2 mM
*Xac*Egl5B	BL21(DE3)	20 °C	16 h	LB	0.1 mM
*Xac*Egl5C	BL21(DE3)	20 °C	16 h	LB	0.1 mM
*Xac*EngXCA	BL21(DE3) ΔslyD pRARE II	20 °C	16 h	Auto-induction	-
XAC0346	BL21(DE3)	20°C	20 h	TB	0.5 mM

Supplementary Table 19. Protein purification conditions.

Protein	Lysis buffer	Affinity buffer	Gradient of imidazole	Size-exclusion buffer
*Xac*Bgl3A	20 mmol.L^{-1} sodium phosphate pH 7.4, 300 mM NaCl, 20 mmol.L^{-1} imidazole, 1 mmol.L^{-1} PMSF, and 0.1 mg. mL^{-1} lysozyme	20 mmol.L^{-1} sodium phosphate pH 7.4, 300 mmol.L^{-1} NaCl	20-500 mmol.L^{-1}	20 mmol.L^{-1} sodium phosphate, pH 7.4 and 150 mmol.L^{-1} NaCl
*Xcc*Xeg74[a]	20 mmol.L^{-1} Tris–HCl pH 8.0, 300 mmol.L^{-1} NaCl, 1 mmol.L^{-1} PMSF	20 mmol.L^{-1} Tris–HCl pH 8.0, 150 mmol.L^{-1} NaCl,	50-300 mmol.L^{-1}	20 mmol.L^{-1} Tris–HCl pH 8.0, 150 mmol.L^{-1} NaCl
*Xac*Xeg74	20 mmol.L^{-1} sodium phosphate pH 7.4, 5 mmol.L^{-1} imidazole, 150 mmol.L^{-1} NaCl, 1 mmol.L^{-1} PMSF	20 mmol.L^{-1} sodium phosphate pH 7.4, 150 mmol.L^{-1} NaCl	5-1000 mmol.L^{-1}	20 mmol.L^{-1} sodium citrate pH 5.5, 150 mmol.L^{-1} NaCl
*Xac*XaeA[b]	20 mmol.L^{-1} sodium phosphate pH 7.4, 5 mmol.L^{-1} imidazole, 150 mmol.L^{-1} NaCl	20 mmol.L^{-1} sodium phosphate pH 7.4, 150 mmol.L^{-1} NaCl	5-1000 mmol.L^{-1}	20 mmol.L^{-1} sodium acetate 5.5, 150 mmol.L^{-1} NaCl
*Xac*GalD	20 mmol.L^{-1} sodium phosphate pH 7.4, 5 mmol.L^{-1} imidazole, 150 mmol.L^{-1} NaCl, 1 mmol.L^{-1} PMSF	20 mmol.L^{-1} sodium phosphate pH 7.4, 150 mmol.L^{-1} NaCl	5-1000 mmol.L^{-1}	20 mmol.L^{-1} Hepes pH 7.5, 150 mmol.L^{-1} NaCl
*Xac*Xyl31	20 mmol.L^{-1} sodium phosphate pH 7.4, 5 mmol.L^{-1} imidazole, 150 mmol.L^{-1} NaCl, 1 mmol.L^{-1} PMSF	20 mmol.L^{-1} sodium phosphate pH 7.4, 150 mmol.L^{-1} NaCl	5-1000 mmol.L^{-1}	20 mmol.L^{-1} Hepes pH 7.5, 150 mmol.L^{-1} NaCl
*Xac*Afc95	20 mmol.L^{-1} sodium phosphate pH 7.4, 5 mmol.L^{-1} imidazole, 150 mmol.L^{-1} NaCl, 1 mmol.L^{-1} PMSF	20 mmol.L^{-1} sodium phosphate pH 7.4, 150 mmol.L^{-1} NaCl	5-1000 mmol.L^{-1}	20 mmol.L^{-1} Hepes pH 7.5, 150 mmol.L^{-1} NaCl
*Xac*Bgl3B	20 mmol.L^{-1} sodium phosphate pH 7.4, 300 mmol.L^{-1} NaCl, 20 mmol.L^{-1} imidazole, 1 mmol.L^{-1} PMSF, and 0.1 mg. mL^{-1} lysozyme	20 mmol.L^{-1} sodium phosphate pH 7.4, 300 mmol.L^{-1} NaCl	20-500 mmol.L^{-1}	20 mmol.L^{-1} sodium phosphate, pH 7.4 and 150 mmol.L^{-1} NaCl
*Xac*Egl9	20 mmol.L^{-1} sodium phosphate pH 7.4, 700 mmol.L^{-1} , NaCl, 5 mmol.L^{-1} imidazole, 5 % glycerol, pH 7.4, 1 mmol.L^{-1} PMSF	20 mmol.L^{-1} sodium phosphate pH 7.4, 700 mmol.L^{-1} NaCl, 5% glycerol	5-500 mmol.L^{-1}	20 mmol.L^{-1} MES, pH 6.0, 700 mmol.L^{-1} NaCl

[a]*Xcc*Xeg74 was subjected to removal of the 6xHis-tag by TEV protease after affinity purification and dialysis to 20 mmol.L^{-1} Tris-HCl at pH 8.0, 300 mmol.L^{-1} NaCl buffer solution. TEV protease reaction was carried for 24 hours at 8 ºC. The protein solution was submitted to size-exclusion chromatography.

[b]*Xac*XaeA was subjected to limited proteolysis after nickel chromatography using 0.01% (*m*/*m*) trypsin for 4 hours at 4°C. Proteolysis reaction was stopped by adding 1 mmol.L^{-1} phenylmethylsulfonil fluoride (PMSF) and the resulting sample was purified by anion exchange chromatography using NaCl linear gradient from 100 mmol.L^{-1} to 1 mol.L^{-1} in a HiTrapQ HP column coupled to an ÄKTA purifier system, using 50 mmol.L^{-1} acetate buffer at pH 5.5. Fractions were analyzed by SDS-PAGE and submitted to size-exclusion chromatography.

Supplementary Table 19. Continued.

Protein	Lysis buffer	Affinity buffer	Gradient of imidazole	Size-exclusion buffer
*Xac*Bgl3C	20 mmol.L^{-1} sodium phosphate, pH 7.4, 300 mmol.L^{-1}NaCl, 20 mmol.L^{-1} imidazole, 1 mmol.L^{-1} PMSF, and 0.1 mg.mL^{-1} lysozyme	20 mmol.L^{-1} sodium phosphate, pH 7.4, 300 mmol.L^{-1} NaCl	20-500 mmol.L^{-1}	20 mmol.L^{-1} sodium phosphate buffer, pH 7.4 and 150 mmol.L^{-1} NaCl
*Xac*Abf43A	20 mmol.L^{-1} sodium phosphate pH 7.4, 150 mmol.L^{-1} NaCl, 5% glycerol, 5 mmol.L^{-1} imidazole, 4 mmol.L^{-1} PMSF, 2 mmol.L^{-1} benzamidine and 0.1 mg.mL^{-1} lysozyme	20 mmol.L^{-1} sodium phosphate pH 7.4, 150 mmol.L^{-1} NaCl and 5% glycerol	5-500 mmol.L^{-1}	20 mmol.L^{-1} Hepes pH 7.5, 150 mmol.L^{-1} NaCl and 10% glycerol
*Xac*Egl5A	20 mmol.L^{-1} sodium phosphate pH 7.4, 500 mmol.L^{-1} NaCl, 5 mmol.L^{-1} imidazole, 1 mmol.L^{-1} PMSF	20 mmol.L^{-1} sodium phosphate pH 7.4, 500 mmol.L^{-1} NaCl and 5% glycerol	5-500 mmol.L^{-1}	20 mmol.L^{-1} sodium phosphate buffer, pH 7.4 and 150 mmol.L^{-1} NaCl
*Xac*Egl5B	20 mmol.L^{-1} sodium phosphate pH 7.4, 500 mmol.L^{-1} NaCl, 5 mmol.L^{-1} imidazole, 1 mmol.L^{-1} PMSF	20 mmol.L^{-1} sodium phosphate pH 7.4, 500 mmol.L^{-1} NaCl	5-500 mmol.L^{-1}	20 mmol.L^{-1} sodium phosphate buffer, pH 7.4 and 150 mmol.L^{-1} NaCl
*Xac*Egl5C	20 mmol.L^{-1} sodium phosphate pH 7.4, 500 mmol.L^{-1} NaCl, 5 mmol.L^{-1} imidazole, 1 mmol.L^{-1} PMSF	20 mmol.L^{-1} sodium phosphate pH 7.4, 500 mmol.L^{-1} NaCl	5-500 mmol.L^{-1}	20 mmol.L^{-1} sodium phosphate buffer, pH 7.4 and 150 mmol.L^{-1} NaCl
*Xac*EngXCA	20 mmol.L^{-1} sodium phosphate pH 7.4, 500 mmol.L^{-1} NaCl, 5 mmol.L^{-1} imidazole, 1 mmol.L^{-1} PMSF	20 mmol.L^{-1} sodium phosphate pH 7.4, 500 mmol.L^{-1} NaCl	5-500 mmol.L^{-1}	20 mmol.L^{-1} sodium phosphate buffer, pH 7.4 and 150 mmol.L^{-1} NaCl
XAC0346	20 mmol.L^{-1} sodium phosphate pH 7.4, 300 mmol.L^{-1} NaCl, 20 mmol.L^{-1} imidazole, 1 mmol.L^{-1} PMSF, and 0.1 mg. mL^{-1} lysozyme	20 mmol.L^{-1} sodium phosphate pH 7.4, 300 mmol.L^{-1} NaCl	20-500 mmol.L^{-1}	20 mmol.L^{-1} sodium phosphate, pH 7.4 and 150 mmol.L^{-1} NaCl

Supplementary Table 20. Crystallization conditions. Proteins were concentrated in the final purification buffer (Supplementary Table 19). Ligand introduced by cocrystallization were incubated for 1 h at 4 °C under mild agitation and centrifuged for 15 min (4 °C) at 14,000 *g* prior to the experiment. Crystallization experiments were performed at 18 °C in a temperature-controlled environment. A 1:1 drop ratio of protein to crystallization solution was employed. All proteins were crystallized by the vapor diffusion technique in hanging or sitting drops.

Enzyme	Enzyme concentration (µmol.L^{-1})	Ligand	Condition	Method
*Xcc*GH74	245	XXG[1]<br>Cocrystallization<br>5 mmol.L^{-1}	0.2 mol.L^{-1} sodium iodide<br>20% (*v*/*v*) PEG 3350<br>0.1 mol.L^{-1} bis-tris propane pH 7.5	Sitting drop
*Xac*XaeA	160	-	0.01 mol.L^{-1} zinc chloride<br>20% (*v*/*v*) PEG 6000<br>0.1 mol.L^{-1} tris pH 7.0	Hanging drop
*Xac*GalD	336	-	0.1 mol.L^{-1} lithium sulfate<br>1.26 mol.L^{-1} ammonium sulfate<br>0.1 mol.L^{-1} tris pH 8.5	Sitting drop
*Xac*GalD	235	Galactose[2]<br>Cocrystallization<br>5 mmol.L^{-1}	1.26 mol.L^{-1} ammonium sulfate<br>8% (*v*/*v*) glycerol<br>0.1 mol.L^{-1} tris pH 8.5	Sitting drop
*Xac*Xyl31	312	-	0.2 mol.L^{-1} potassium nitrate<br>20% (*v*/*v*) PEG 3350	Sitting drop
*Xac*Xyl31	312	Xylose[2]<br>4 h soaking<br>10 mmol.L^{-1}	0.2 mol.L^{-1} potassium formate<br>20% (*v*/*v*) PEG 3350	Sitting drop
*Xac*Afc95	185	-	20% (*v*/*v*) 2-methyl-2,4-pentanediol (MPD)<br>0.1 mol.L^{-1} tris pH 8.5	Sitting drop

[1]Megazyme
[2]Sigma-Aldrich

Supplementary Table 21. Enzymatic reaction conditions of XyGUL and accessory enzymes used for activity detection screening or specific activity assays.

Protein	Enzyme concentration	Buffer	Reaction with polymeric substrates	Reaction with synthetic substrates
*Xac*Bgl3A	100 µg.mL^{-1}	40 mmol.L^{-1} McIlvaine buffer pH 6.0	2.5 mg.mL^{-1} of substrate, 20 hours at 35 °C	2 mmol.L^{-1} of substrate, 1 hour at 35 °C
*Xac*Bgl3B	100 µg.mL^{-1}	40 mmol.L^{-1} McIlvaine buffer pH 6.0	2.5 mg.mL^{-1} of substrate, 20 hours at 35 °C	2 mmol.L^{-1} of substrate, 1 hour at 35 °C
*Xac*Bgl3C	100 µg.mL^{-1}	40 mmol.L^{-1} McIlvaine buffer pH 6.0	2.5 mg.mL^{-1} of substrate, 20 hours at 35 °C	2 mmol.L^{-1} of substrate, 1 hour at 35 °C
*Xac*Xeg74	150 µg.mL^{-1}	80 mmol.L^{-1} McIlvaine buffer pH 5.5	2 mg.mL^{-1} of substrate, 16 hours at 35 °C	2 mmol.L^{-1} of substrate, 1 hour at 35 °C
*Xac*XaeA	150 µg.mL^{-1}	80 mmol.L^{-1} HEPES buffer pH 7.5	2 mg.mL^{-1} of substrate, 16 hours at 35 °C	2 mmol.L^{-1} of substrate, 1 hour at 35 °C
*Xac*GalD	150 µg.mL^{-1}	80 mmol.L^{-1} McIlvaine buffer pH 5.5	2 mg.mL^{-1} of substrate, 16 hours at 35 °C	2 mmol.L^{-1} of substrate, 1 hour at 35 °C
*Xac*Xyl31	150 µg.mL^{-1}	80 mmol.L^{-1} McIlvaine buffer pH 5.5	2 mg.mL^{-1}of substrate, 16 hours at 35 °C	2 mmol.L^{-1} of substrate, 1 hour at 35 °C
*Xac*Afc95	150 µg.mL^{-1}	80 mmol.L^{-1} McIlvaine buffer pH 5.5	2 mg.mL^{-1} of substrate, 16 hours at 35 °C	2 mmol.L^{-1} of substrate, 1 hour at 35 °C
*Xac*Egl9	100 µg.mL^{-1}	40 mmol.L^{-1} MES buffer pH 6.0	2.5 mg.mL^{-1} of substrate, 10 min at 35 °C	-
*Xac*Abf43A	80 µg.mL^{-1}	40 mmol.L^{-1} McIlvaine buffer pH 6.0	2.5 mg.mL^{-1} of substrate, 6 hours at 37 °C	2.5 mmol.L^{-1} of substrate, 4 hours at 37 °C
*Xac*Egl5A	20 µg.mL^{-1}	40 mmol.L^{-1} McIlvaine buffer pH 6.0	2.5 mg.mL^{-1} of substrate, 10 min at 35 °C	-
*Xac*Egl5B	10 µg.mL^{-1}	40 mmol.L^{-1} McIlvaine buffer pH 5.0	2.5 mg.mL^{-1} of substrate, 10 min at 35 °C	-
*Xac*Egl5C	60 µg.mL^{-1}	40 mmol.L^{-1} McIlvaine buffer pH 5.0	2.5 mg.mL^{-1} of substrate, 10 min at 35 °C	-
*Xac*EngXCA	6 µg.mL^{-1}	40 mmol.L^{-1} McIlvaine buffer pH 5,5	2.5 mg.mL^{-1} of substrate, 10 min at 65 °C	-
XAC0346	200 µg.mL^{-1}	40 mmol.L^{-1} McIlvaine buffer pH 7.0	2.5 mg.mL^{-1} of substrate, 20 hours at 35 °C	-

Supplementary Table 22. Summary of RNA-seq data. Each sample represents a biologically independent experiment.

Sample	Input reads	QC reads	QC reads (%)	rRNA reads (%)	Mapped reads (%)
XVM2m_Glucose 1	2895851	2524189	87.17	5.64	87.68
XVM2m_Glucose 2	5660364	4990317	88.16	1.92	91.82
XVM2m_Glucose 3	5540215	5018731	90.59	2.58	68.56
XVM2m_Glucose 4	5164471	4599580	89.06	0.79	69.5
XVM2m_Glucose 5	4847539	4237597	87.42	14.36	61.53
XVM2m_Glucose 6	23704254	19279704	81.33	22.76	73.24
XVM2m_XyGOs 1	11228037	9610422	85.59	1.26	86.44
XVM2m_XyGOs 2	5107135	4483206	87.78	4.19	86.75
XVM2m_XyGOs 3	3738110	3113289	83.29	0.87	85.47
XVM2m_XyGOs 4	4970294	4324291	87.00	9.53	78.12

Supplementary Table 23. Classification of candidate reference genes based on the analysis of RNA-seq data under different culture conditions[a]. The genes are classified from the lowest to the highest coefficient of variation. Values highlighted in bold indicate genes with normal distribution according to the Shapiro-Wilk test (*p*-value > 0.05). CV: coefficient of variation; MFC: ratio of the maximum to minimum TPM value of each gene. Genes with the highest mean TPM values and *p*-value > 0.05 were considered for further analysis, except for XAC1735 due to the presence of non-specific products in preliminary RT-PCR assays.

Locus	**Description**	**Mean TPM**	**CV**	**MFC**	***p*-value**
XAC4132	acid phosphatase	1.1	0.13	1.48	2.80E-06
XAC1735	hfq	555.62	0.17	1.87	**0.48**
XAC2293	epimerase	137.66	0.18	1.9	**0.16**
XAC0145	conserved hypothetical protein	5.72	0.19	2.18	**0.73**
XAC0328	multidrug efflux transporter	5.05	0.19	2.41	**0.29**
XAC2491	conserved hypothetical protein	120.94	0.19	1.94	0.03
XAC2177	hypothetical protein	305.45	0.2	1.94	**0.08**
XAC4047	glutathione S-transferase	111.24	0.2	2.67	**0.94**
XAC4142	conserved hypothetical protein	1.32	0.2	2.01	0.01
XAC3440	H+ translocating pyrophosphate synthase	34.53	0.2	2.29	**0.33**
XAC2594	threonyl-tRNA synthetase	291.65	0.2	2.44	**0.44**
XAC2416	virulence regulator	457.34	0.2	2.26	0.02
XAC3667	outer membrane protein	115.84	0.2	2.35	**0.14**
XAC3701	Na+:H+ antiporter	37.23	0.2	2.68	**0.53**
XAC4140	ClpB	2.34	0.21	2.11	**0.11**
XAC4218	sec-independent protein translocase	584.53	0.21	2.47	**0.14**
XACb0065	avirulence protein	78.13	0.21	2.69	**0.27**
XAC3784	hypothetical protein	120.9	0.21	2.69	**0.19**
XAC0924	NAD(P) transhydrogenase subunidade β	52.1	0.21	2.16	**0.41**
XAC4116	serine/threonine kinase	2.12	0.21	2.48	**0.77**

[a] Modified XVM2 medium containing glucose, β-1,4-glucobiose, starch, xyloglucan oligosaccharides, xylan oligosaccharides, arabinoxylan oligosaccharides or galactomannan oligosaccharides as sole carbohydrate source.

Supplementary Table 24. Analysis of gene expression stability of candidate reference genes. The variation in expression levels was evaluated for *X. citri* growth in minimal medium XVM2m containing different carbohydrate sources. Results were generated by BestKeeper[96], NormFinder[97], and RefFinder[98] algorithms. The ranking of each analysis is in parentheses, with 1 being considered the most stable and 4 being the least stable.

Locus	**BestKeeper** SD [±CP]	**NormFinder** Stability value	**RefFinder** Geometric mean
XAC4218	0.24 (1)	0.087 (1)	1.68 (2)
XAC2177	0.30 (2)	0.201 (2)	1.0 (1)
XAC4047	0.39 (3)	0.245 (3)	3.22 (3)
XAC2293	0.44 (4)	0.317 (4)	3.72 (4)

Supplementary Table 25. Primers used in RT-qPCR assays. Standard curves were generated from serially diluted samples (10X) to evaluate the efficiency of RT-qPCR reactions (E) and correlation coefficients (R^2). The reactions with a final volume of 10 µL and with 2 ng of RNA were performed using the following protocol: 48 °C for 30 min (cDNA synthesis), followed by a step at 95 ° C for 10 min, and then 40 cycles at 95 °C for 15 sec (denaturation), 60 °C for 30 sec (annealing) and 72 °C for 30 sec (extension). Primers corresponding to candidate reference genes are highlighted in bold[a].

Locus	**Forward primer**	**Reverse primer**	**Concentration (µM)**	**E[a] (%)**	**R^2**	**Amplicon length (pb)**	**Reference**
XAC0394	AAGCCAGCTCCGAGATTCAC	CTTCCTTGCCATCGCCGTAA	0.15	110	0.992	96	This study
XAC0397	ACGGGCAGATGAGTTCGTTGA	GCCACGTTGAAGTCCAGATCATT	0.10	97	0.996	109	This study
XAC0409	TCCTATTCGCTGGAGGTGCTA	TTCGGTGGGTGTCGAGATCA	0.20	101	0.999	105	This study
XAC0416	CGCCAACTCGTCCTTCTTTCAG	GGTCCAGTTGCTTTTCCGAGATG	0.10	102	0.999	95	This study
XAC1265	CACCTGAGCGTTGGTCCATA	AGCCAGGCAATCGAGAACT	0.15	105	0.999	104	This study
XAC1266	AGCGATCTCTGCGTTGTCCTAC	ATACGCATCTTCGGCCTCTTCCTGA	0.10	107	0.994	211	[99]
**XAC2293**	CGGATCGTGCTGCTGGAAAT	GCCGCAATCGCCTAACACT	0.20	104	0.994	108	This study
**XAC4047**	CACCCTCAAGCCGGTCAAC	CGGTGCATGGTCGACGAT	0.10	101	0.999	103	This study
**XAC4218**	ATCGTGCTGGTGATCGTGTT	TCGTCGTGCATGCCTTTCTT	0.10	104	0.995	107	This study
**XAC2177**	GGTGTAGCCGTCTTTGTCTGC	CAGAACATCGCCTACTGCCAT	0.15	101	0.999	126	This study

[a] Only primers with amplification efficiency between 90% and 110% were considered for RT-qPCR assays.

Supplementary Table 26. List of primers used for gene knockout experiments in *X. citri*.

Construction	Primer sequence (5'→3')
ΔXAC1768-69-F1	GGATCCCGCTATGGCAAATTCCGGTACTCC
ΔXAC1768-69-F2	CATATGTTGCGGGCCAGCCTGTAGAAC
ΔXAC1768-69-R1	CATATGCATCTGAATCCCTCCCAGGACG
ΔXAC1768-69-R2	AAGCTTATCGCCAGTGGCTGGTTGTGGAAT
ΔXAC1768-F1	GGATCCCGCTATGGCAAATTCCGGTACTCC
ΔXAC1768-F2	CATATGGCGCATGAACTGGTAAGTGC
ΔXAC1768-R1	CATATGCATCTGAATCCCTCCCAGGACG
ΔXAC1768-R2	AAGCTTAGATCGGTCTGGGTGTACCA
ΔXAC1769-F1	GGATCCAGGCCAATCAGTACGACACC
ΔXAC1769-F2	CATATGTTGCGGGCCAGCCTGTAGAAC
ΔXAC1769-R1	CATATGGATCGAACGCTGACCCACAG
ΔXAC1769-R2	AAGCTTATCGCCAGTGGCTGGTTGTGGAAT
ΔXAC1770-F1	GGATCCCTGGAATTTGCGCCCAACAACATCAAC
ΔXAC1770-F2	CATATGTATGGCGATCCACGATGAGCGC
ΔXAC1770-R1	CATATGCATTCCTCCGGCTCCCCTCG
ΔXAC1770-R2	AAGCTTCTTCCACGCGGACCGCAATG
ΔXAC1777-F1	GGATCCATACCGCGTTGAACCGTGCC
ΔXAC1777-F2	CATATGGAGCAGATGGAGGGCTGATGG
ΔXAC1777-R1	CATATGCATGGGTTCACCTGGGAACATGC
ΔXAC1777-R2	AAGCTTGACCTGATACACCTTGGGCATTGAG

Supplementary Table 27. List of primers used for gene knockout verification by PCR and DNA sequencing.

Name	Primer sequence (5'→3')
1768-69_seqF	GGAATAGCAGCCTGTGAAATTTTCA
1768-69_seqR	TGTGCCACTGATACGGGTCTG
1768_seqF	GGAATAGCAGCCTGTGAAATTTTCA
1768_seqR	GTACTTCTAGTTTTTAGCGGGC
1769_seqF	ACGTTCAAATCAGTGCCATGT
1769_seqR	TGTGCCACTGATACGGGTCTG
1770_seqF	AACATCAATGATGTGTGGTCGATTT
1770_seqR	GCTTGCCGTCGAAGCTGA
1777_seqF	AGCGTTACGCCAGCTTCGACA
1777_seqR	TAAGTGCTGGATTGGCCTTCCCTT

REFERENCES

1. Sievers, F. *et al.* Fast, scalable generation of high-quality protein multiple sequence alignments using Clustal Omega. *Mol. Syst. Biol.* **7**, 539 (2011).

2. Robert, X. & Gouet, P. Deciphering key features in protein structures with the new ENDscript server. *Nucleic Acids Res.* **42**, W320–W324 (2014).

3. Hsieh, Y. S. Y. & Harris, P. J. Xyloglucans of monocotyledons have diverse structures. *Mol. Plant* **2**, 943–965 (2009).

4. Hilz, H., de Jong, L. E., Kabel, M. A., Schols, H. A. & Voragen, A. G. J. A comparison of liquid chromatography, capillary electrophoresis, and mass spectrometry methods to determine xyloglucan structures in black currants. *J. Chromatogr. A* **1133**, 275–286 (2006).

5. Lerouxel, O. *et al.* Rapid structural phenotyping of plant cell wall mutants by enzymatic oligosaccharide fingerprinting. *Plant Physiol.* **130**, 1754–1763 (2002).

6. Domon, B. & Costello, C. E. A systematic nomenclature for carbohydrate fragmentations in FAB-MS/MS spectra of glycoconjugates. *Glycoconj. J.* **5**, 397–409 (1988).

7. Jurrus, E. *et al.* Improvements to the APBS biomolecular solvation software suite. *Protein Sci.* **27**, 112–128 (2018).

8. Landau, M. *et al.* ConSurf 2005: the projection of evolutionary conservation scores of residues on protein structures. *Nucleic Acids Res.* **33**, W299–W302 (2005).

9. Glaser, F. *et al.* ConSurf: Identification of functional regions in proteins by surface-mapping of phylogenetic information. *Bioinformatics* **19**, 163–164 (2003).

10. Bitto, E. *et al.* The structure at 1.6 Å resolution of the protein product of the At4g34215 gene from *Arabidopsis thaliana*. *Acta Crystallogr. Sect. D Biol. Crystallogr.* **61**, 1655–1661 (2005).

11. Till, M. *et al.* Structure and function of an acetyl xylan esterase (Est2A) from the rumen bacterium *Butyrivibrio proteoclasticus*. *Proteins Struct. Funct. Bioinforma.* **81**, 911–917 (2013).

12. Michalak, L. *et al.* A pair of esterases from a commensal gut bacterium remove acetylations from all positions on complex β-mannans. *Proc. Natl. Acad. Sci. U. S. A.* **117**, 7122–7130 (2020).

13. Correia, M. A. S. *et al.* Crystal structure of a cellulosomal family 3 carbohydrate esterase from *Clostridium thermocellum* provides insights into the mechanism of substrate secognition. *J. Mol. Biol.* **379**, 64–72 (2008).

14. Mølgaard, A., Kauppinen, S. & Larsen, S. Rhamnogalacturonan acetylesterase elucidates the structure and function of a new family of hydrolases. *Structure* **8**, 373–383 (2000).

15. Henze, M. *et al.* Rational design of a glycosynthase by the crystal structure of β-galactosidase from *Bacillus circulans* (BgaC) and its use for the synthesis of *N*-acetyllactosamine type 1 glycan structures. *J. Biotechnol.* **191**, 78–85 (2014).

16. Ohto, U. *et al.* Crystal structure of human β-galactosidase: Structural basis of G M1 gangliosidosis and morquio B diseases. *J. Biol. Chem.* **287**, 1801–1812 (2012).

17. Cartmell, A. *et al.* A surface endogalactanase in *Bacteroides thetaiotaomicron* confers keystone status for arabinogalactan degradation. *Nat. Microbiol.* **3**, 1314–1326 (2018).

18. Maksimainen, M. M., Lampio, A., Mertanen, M., Turunen, O. & Rouvinen, J. The crystal structure of acidic β-galactosidase from *Aspergillus oryzae*. *Int. J. Biol. Macromol.* **60**, 109–115 (2013).

19. Wengelnik, K., Marie, C., Russel, M. & Bonas, U. Expression and localization of HrpA1, a protein of *Xanthomonas campestris* pv. *vesicatoria* essential for pathogenicity and induction of the hypersensitive

reaction. *J. Bacteriol.* **178**, 1061–1069 (1996).

20. Serrania, J., Vorhölter, F. J., Niehaus, K., Pühler, A. & Becker, A. Identification of *Xanthomonas campestris* pv. *campestris* galactose utilization genes from transcriptome data. *J. Biotechnol.* **135**, 309–317 (2008).

21. Vorhölter, F. J. *et al.* The genome of *Xanthomonas campestris* pv. *campestris* B100 and its use for the reconstruction of metabolic pathways involved in xanthan biosynthesis. *J. Biotechnol.* **134**, 33–45 (2008).

22. Schatschneider, S. *et al.* Metabolic flux pattern of glucose utilization by *Xanthomonas campestris* pv. *campestris*: Prevalent role of the Entner-Doudoroff pathway and minor fluxes through the pentose phosphate pathway and glycolysis. *Mol. Biosyst.* **10**, 2663–2676 (2014).

23. Schatschneider, S. *et al.* Establishment, in silico analysis, and experimental verification of a large-scale metabolic network of the xanthan producing *Xanthomonas campestris* pv. *campestris* strain B100. *J. Biotechnol.* **167**, 123–134 (2013).

24. Yew, W. S. *et al.* Evolution of enzymatic activities in the enolase superfamily: L-fuconate dehydratase from *Xanthomonas campestris*. *Biochemistry* **45**, 14582–14597 (2006).

25. Seydel, A., Gounon, P. & Pugsley, A. P. Testing the '+2 rule' for lipoprotein sorting in the *Escherichia coli* cell envelope with a new genetic selection. *Mol. Microbiol.* **34**, 810–821 (1999).

26. Almagro Armenteros, J. J. *et al.* SignalP 5.0 improves signal peptide predictions using deep neural networks. *Nat. Biotechnol.* **37**, 420–423 (2019).

27. Krogh, A., Larsson, B., Von Heijne, G. & Sonnhammer, E. L. L. Predicting transmembrane protein topology with a hidden Markov model: Application to complete genomes. *J. Mol. Biol.* **305**, 567–580 (2001).

28. Yu, C. S., Chen, Y. C., Lu, C. H. & Hwang, J. K. Prediction of protein subcellular localization. *Proteins Struct. Funct. Genet.* **64**, 643–651 (2006).

29. Ferreira, R. M. *et al.* Unravelling potential virulence factor candidates in *Xanthomonas citri* subsp. *citri* by secretome analysis. *PeerJ* **4**, e1734 (2016).

30. Pellock, S. J. *et al.* Three structurally and functionally distinct -glucuronidases from the human gut microbe *Bacteroides uniformis*. *J. Biol. Chem.* **293**, 18559–18573 (2018).

31. Míguez Amil, S. *et al.* The cryo-EM Structure of *Thermotoga maritima* β-Galactosidase: Quaternary Structure Guides Protein Engineering. *ACS Chem. Biol.* **15**, 179–188 (2020).

32. Ishikawa, K. *et al.* Crystal structure of β-galactosidase from *Bacillus circulans* ATCC 31382 (BgaD) and the construction of the thermophilic mutants. *FEBS J.* **282**, 2540–2552 (2015).

33. Kong, C. *et al.* Complete genome sequence of strain WHRI 3811 race 1 of *Xanthomonas campestris* pv. *campestris*, the causal agent of black rot of cruciferous vegetables. *Mol. Plant-Microbe Interact.* **32**, 1571–1573 (2019).

34. Lema, M., Cartea, M. E., Sotelo, T., Velasco, P. & Soengas, P. Discrimination of *Xanthomonas campestris* pv. *campestris* races among strains from northwestern Spain by *Brassica* spp. genotypes and rep-PCR. *Eur. J. Plant Pathol.* **133**, 159–169 (2012).

35. Bogdanove, A. J. *et al.* Two new complete genome sequences offer insight into host and tissue specificity of plant pathogenic *Xanthomonas* spp. *J. Bacteriol.* **193**, 5450–5464 (2011).

36. Fargier, E. & Manceau, C. Pathogenicity assays restrict the species *Xanthomonas campestris* into three pathovars and reveal nine races within *X. campestris* pv. *campestris*. *Plant Pathol.* **56**, 805–818 (2007).

37. Richard, D. *et al.* Complete genome sequences of six copper-resistant *Xanthomonas* strains causing

bacterial spot of solaneous plants, belonging to *X. gardneri*, *X. euvesicatoria*, and *X. vesicatoria*, using long-read technology. *Genome Announc.* **5**, (2017).

38. Dhakal, U., Dobhal, S., Alvarez, A. M. & Arif, M. Phylogenetic analyses of xanthomonads causing bacterial leaf spot of tomato and pepper: *Xanthomonas euvesicatoria* revealed homologous populations despite distant geographical distribution. *Microorganisms* **7**, 462 (2019).

39. Jacques, M. A. *et al.* Using ecology, physiology, and genomics to understand host specificity in *Xanthomonas*. *Annual Review of Phytopathology* **54**, 163–187 (2016).

40. Zhang, X. & Babadoost, M. Characteristics of *Xanthomonas cucurbitae* isolates from pumpkins and survival of the bacterium in pumpkin seeds. *Plant Dis.* **102**, 1779–1784 (2018).

41. Ravanlou, A. & Babadoost, M. Development of bacterial spot, incited by *Xanthomonas cucurbitae*, in pumpkin fields. *HortScience* **50**, 714–720 (2015).

42. Hayward, A. C. The hosts of *Xanthomonas*. in *Xanthomonas Ch. 1* 1–119 (Springer, Dordrecht, 1993). doi:10.1007/978-94-011-1526-1_1

43. Rani, M., Weadge, J. T. & Jabaji, S. Isolation and characterization of biosurfactant-producing bacteria from oil well batteries with antimicrobial activities against food-borne and plant pathogens. *Front. Microbiol.* **11**, (2020).

44. Garita-Cambronero, J., Palacio-Bielsa, A. & Cubero, J. *Xanthomonas arboricola* pv. *pruni*, causal agent of bacterial spot of stone fruits and almond: its genomic and phenotypic characteristics in the *X. arboricola* species context. *Mol. Plant Pathol.* **19**, 2053–2065 (2018).

45. Cesbron, S. *et al.* Comparative genomics of pathogenic and nonpathogenic strains of *Xanthomonas arboricola* unveil molecular and evolutionary events linked to pathoadaptation. *Front. Plant Sci.* **6**, (2015).

46. Fernandes, C., Blom, J., Pothier, J. F. & Tavares, F. High-quality draft genome sequence of *Xanthomonas sp.* strain CPBF 424, a walnut-pathogenic strain with atypical features. *Microbiol. Resour. Announc.* **7**, (2018).

47. Wang, H., McTavish, C. & Turechek, W. W. Colonization and movement of *Xanthomonas fragariae* in strawberry tissues. *Phytopathology* **108**, 681–690 (2018).

48. Kastelein, P. *et al.* Development of *Xanthomonas fragariae* populations and disease progression in strawberry plants after spray-inoculation of leaves. *Plant Pathol.* **63**, 255–263 (2014).

49. Samanta, J. N., Mandal, K. & Maiti, S. A novel pathovar of *Xanthomonas axonopodis* causes gumming of Guggal (*Commiphora wightii*). *Eur. J. Plant Pathol.* **135**, 115–125 (2013).

50. Jalan, N. *et al.* Comparative genomic analysis of *Xanthomonas axonopodis* pv. *citrumelo* F1, which causes citrus bacterial spot disease, and related strains provides insights into virulence and host specificity. *J. Bacteriol.* **193**, 6342–6357 (2011).

51. Gottwald, T. R., Graham, J. H., Civerolo, E. L., Barrett, H. C. & Hearn, C. J. Differential host range reaction of citrus and citrus relatives to citrus canker and citrus bacterial spot determined by leaf mesophyll susceptibility. *Plant Dis.* **77**, 1004–1009 (1993).

52. Thieme, F. *et al.* Insights into genome plasticity and pathogenicity of the plant pathogenic bacterium *Xanthomonas campestris* pv. *vesicatoria* revealed by the complete genome sequence. *J. Bacteriol.* **187**, 7254–7266 (2005).

53. Abrahamian, P. *et al.* Molecular epidemiology of *Xanthomonas perforans* outbreaks in tomato plants from transplant to field as determined by singlenucleotide polymorphism analysis. *Appl. Environ. Microbiol.* **85**, (2019).

54. Ruh, M., Briand, M., Bonneau, S., Jacques, M. A. & Chen, N. W. G. *Xanthomonas* adaptation to common bean is associated with horizontal transfers of genes encoding TAL effectors. *BMC Genomics* **18**, 670 (2017).

55. Darrasse, A. *et al.* Genome sequence of *Xanthomonas fuscans* subsp. *fuscans* strain 4834-R reveals that flagellar motility is not a general feature of xanthomonads. *BMC Genomics* **14**, 761 (2013).

56. EFSA Panel on Plant Health (PLH). Scientific Opinion on the pest categorisation of *Xanthomonas axonopodis* pv. *phaseoli* and *Xanthomonas fuscans* subsp. *fuscans*. *EFSA J.* **12**, 3856 (2014).

57. Ruh, M., Briand, M., Bonneau, S., Jacques, M. A. & Chen, N. W. G. First complete genome sequences of *Xanthomonas citri* pv. *vignicola* Strains CFBP7111, CFBP7112, and CFBP7113 obtained using long-read technology. *Genome Announc.* **5**, (2017).

58. Moretti, C., Mondjana, A. M., Zazzerini, A. & Buonaurio, R. Occurrence of leaf spot on cowpea (*Vigna unguiculata*) caused by *Xanthomonas axonopodis* pv. *vignicola* in Mozambique. *Plant Pathol.* **56**, 347 (2007).

59. Fonseca, N. P. *et al.* Analyses of seven new genomes of *Xanthomonas citri* pv. *aurantifolii* strains, causative agents of citrus canker B and C, show a reduced repertoire of pathogenicity-related genes. *Front. Microbiol.* **10**, (2019).

60. Jalan, N. *et al.* Complete genome sequence of *Xanthomonas citri* subsp. *citri* strain AW12879, a restricted-host-range citrus canker-causing bacterium. *Genome Announc.* **1**, (2013).

61. Midha, S. & Patil, P. B. Genomic insights into the evolutionary origin of *Xanthomonas axonopodis* pv. *citri* and its ecological relatives. *Appl. Environ. Microbiol.* **80**, 6266–6279 (2014).

62. Brunings, A. M. & Gabriel, D. W. *Xanthomonas citri*: Breaking the surface. *Mol. Plant Pathol.* **4**, 141–157 (2003).

63. Da Silva, A. C. R. *et al.* Comparison of the genomes of two *Xanthomonas* pathogens with differing host specificities. *Nature* **417**, 459–463 (2002).

64. Al-Mousawi, A. H. Ultrastructural studies of a compatible interaction between *Xanthomonas campestris* pv. *malvacearum* and Cotton. *Phytopathology* **72**, 1222 (1982).

65. Phillips, A. Z. *et al.* Genomics-enabled analysis of the emergent disease cotton bacterial blight. *PLoS Genet.* **13**, (2017).

66. Sharma, V., Midha, S., Ranjan, M., Pinnaka, A. K. & Patil, P. B. Genome sequence of *Xanthomonas axonopodis* pv. *punicae* strain LMG 859. *J. Bacteriol.* **194**, 2395–2395 (2012).

67. Sharma, J. *et al.* Pomegranate bacterial blight: symptomatology and rapid inoculation technique for *Xanthomonas axonopodis* pv. *punicae*. *J. Plant Pathol.* **99**, 109–119 (2017).

68. Sutruedee, P. *et al.* Hemin transported protein of *Xanthomonas axonopodis* pv. *glycines* functions on leaf colonization and virulence on soybean. *African J. Microbiol. Res.* **7**, 4990–5003 (2013).

69. G, K., Julian, S., David, S., Jerome, K. & E, K. Comparative pathogenicity studies of the *Xanthomonas vasicola* species on maize, sugarcane and banana. *African J. Plant Sci.* **9**, 385–400 (2015).

70. Studholme, D. J. *et al.* Transfer of *Xanthomonas campestris* pv. *arecae* and *X. campestris* pv. *musacearum* to *X. vasicola* (Vauterin) as *X. vasicola* pv. *arecae* comb. nov. and *X. vasicola* pv. *musacearum* comb. nov. and desc. *Phytopathology* **110**, 1153–1160 (2020).

71. Lang, J. M. *et al.* Detection and characterization of *Xanthomonas vasicola* pv. *vasculorum* (Cobb 1894) comb. nov. causing bacterial leaf streak of corn in the United States. *Phytopathology* **107**, 1312–1321 (2017).

72. Nakato, G. V., Wicker, E., Coutinho, T. A., Mahuku, G. & Studholme, D. J. A highly specific tool for

identification of *Xanthomonas vasicola* pv. *musacearum* based on five Xvm-specific coding sequences. *Heliyon* **4**, e01080 (2018).

73. Wonni, I. *et al.* Analysis of *Xanthomonas oryzae* pv. *oryzicola* population in Mali and Burkina Faso reveals a high level of genetic and pathogenic diversity. *Phytopathology* **104**, 520–531 (2014).

74. Lang, J. M. *et al.* A pathovar of *Xanthomonas oryzae* infecting wild grasses provides insight into the evolution of pathogenicity in rice agroecosystems. *Front. Plant Sci.* **10**, (2019).

75. Niño-Liu, D. O., Ronald, P. C. & Bogdanove, A. J. *Xanthomonas oryzae* pathovars: model pathogens of a model crop. *Mol. Plant Pathol.* **7**, 303–324 (2006).

76. Ryan, R. P. *et al.* Pathogenomics of *Xanthomonas*: understanding bacterium-plant interactions. *Nat. Rev. Microbiol.* **9**, 344–355 (2011).

77. Jaenicke, S. *et al.* Complete genome sequence of the barley pathogen *Xanthomonas translucens* pv. *translucens* DSM 18974T (ATCC 19319T). *Genome Announc.* **4**, (2016).

78. Sapkota, S., Mergoum, M. & Liu, Z. The translucens group of *Xanthomonas translucens*: complicated and important pathogens causing bacterial leaf streak on cereals. *Mol. Plant Pathol.* **21**, 291–302 (2020).

79. Peng, Z. *et al.* *Xanthomonas translucens* commandeers the host rate-limiting step in ABA biosynthesis for disease susceptibility. *Proc. Natl. Acad. Sci. U. S. A.* **116**, 20938–20946 (2019).

80. Langlois, P. A. *et al.* Characterization of the *Xanthomonas translucens* complex using draft genomes, comparative genomics, phylogenetic analysis, and diagnostic LAMP assays. *Phytopathology* **107**, 519–527 (2017).

81. Cohen, S. P. *et al.* High-quality genome resource of *Xanthomonas hyacinthi* generated via long-read sequencing. *Plant Dis.* **104**, 1011–1012 (2020).

82. Bansal, K. *et al.* Ecological and evolutionary insights into pathogenic and non-pathogenic rice associated *Xanthomonas*. *bioRxiv* 453373 (2018). doi:10.1101/453373

83. Fang, Y. *et al.* Genome sequence of *Xanthomonas sacchari* R1, a biocontrol bacterium isolated from the rice seed. *J. Biotechnol.* **206**, 77–78 (2015).

84. Pieretti, I. *et al.* Genomic insights into strategies used by *Xanthomonas albilineans* with its reduced artillery to spread within sugarcane xylem vessels. *BMC Genomics* **13**, 658 (2012).

85. Agirre, J. *et al.* Privateer: Software for the conformational validation of carbohydrate structures. *Nat. Struct. Mol. Biol.* **22**, 833–834 (2015).

86. Cremer, D. & Pople, J. A. A general definition of ring puckering coordinates. *J. Am. Chem. Soc.* **97**, 1354–1358 (1975).

87. Miller, G. L. Use of dinitrosalicylic acid reagent for determination of reducing sugar. *Anal. Chem.* **31**, 426–428 (1959).

88. Piiadov, V., Ares de Araújo, E., Oliveira Neto, M., Craievich, A. F. & Polikarpov, I. SAXSMoW 2.0: online calculator of the molecular weight of proteins in dilute solution from experimental SAXS data measured on a relative scale. *Protein Sci.* **28**, 454–463 (2019).

89. Konarev, P. V., Volkov, V. V., Sokolova, A. V., Koch, M. H. J. & Svergun, D. I. PRIMUS: A Windows PC-based system for small-angle scattering data analysis. *J. Appl. Crystallogr.* **36**, 1277–1282 (2003).

90. Krissinel, E. & Henrick, K. 'Protein interfaces, surfaces and assemblies' service PISA at the European Bioinformatics Institute. (http://www.ebi.ac.uk/pdbe/prot_int/pistart.html), inference of macromolecular assemblies from crystalline state. *J. Mol. Biol.* **372**, 774–797 (2007).

91. Cheng, W. *et al.* Structural insights into the substrate specificity of *Streptococcus pneumoniae* β(1,3)-

galactosidase BgaC. *J. Biol. Chem.* **287**, 22910–22918 (2012).

92. Schröder, S. P. *et al.* Towards broad spectrum activity-based glycosidase probes: Synthesis and evaluation of deoxygenated cyclophellitol aziridines. *Chem. Commun.* **53**, 12528–12531 (2017).

93. Rojas, A. L. *et al.* Crystal structures of β-galactosidase from *Penicillium sp.* and its complex with galactose. *J. Mol. Biol.* **343**, 1281–1292 (2004).

94. Maksimainen, M. *et al.* Crystal structures of *Trichoderma reesei* β-galactosidase reveal conformational changes in the active site. *J. Struct. Biol.* **174**, 156–163 (2011).

95. de Melo, R. R. *et al.* Identification of a cold-adapted and metal-stimulated β-1,4-glucanase with potential use in the extraction of bioactive compounds from plants. *Int. J. Biol. Macromol.* **166**, 190–199 (2021).

96. Pfaffl, M. W., Tichopad, A., Prgomet, C. & Neuvians, T. P. Determination of stable housekeeping genes, differentially regulated target genes and sample integrity: BestKeeper - Excel-based tool using pair-wise correlations. *Biotechnol. Lett.* **26**, 509–515 (2004).

97. Andersen, C. L., Jensen, J. L. & Ørntoft, T. F. Normalization of real-time quantitative reverse transcription-PCR data: A model-based variance estimation approach to identify genes suited for normalization, applied to bladder and colon cancer data sets. *Cancer Res.* **64**, 5245–5250 (2004).

98. Xie, F., Xiao, P., Chen, D., Xu, L. & Zhang, B. miRDeepFinder: a miRNA analysis tool for deep sequencing of plant small RNAs. *Plant Mol. Biol.* **80**, 75–84 (2012).

99. Andrade, M. O., Farah, C. S. & Wang, N. The post-transcriptional regulator *rsmA*/*csrA* activates T3SS by stabilizing the 5′ UTR of *hrpG*, the master regulator of *hrp*/*hrc* genes, in *Xanthomonas*. *PLoS Pathog.* **10**, e1003945 (2014).
