# Supplementary material for: Xyloglucan processing machinery in Xanthomonas pathogens and its role in the transcriptional activation of virulence factors
Source: Nat Commun. 2021 Jun 30;12:4049. doi: 10.1038/s41467-021-24277-4 (PMC8245568; doi:10.1038/s41467-021-24277-4)
Supplement: Supplementary file 3 — Description of Additional Supplementary Files [file 41467_2021_24277_MOESM3_ESM.pdf]

Description of Additional Supplementary Files

File Name: Supplementary Data 1
Description: Differentially expressed genes in RNA-seq assays of *X. citri* pv. *citri* 306 strain. Cells were grown in minimal medium XVM2m containing xyloglucan oligosaccharides, and the $\log_2$ fold change values are relative to the minimal medium XVM2m containing glucose as a reference. Genes were considered differentially expressed according to Wald test implemented in the DESeq2 package. p-values were adjusted for multiple tests using Benjamini-Hochberg (BH) method also implemented in the DESeq2 package. Thresholds: $|\log_2 \text{fold change}| \geq 1$ and p-adjusted ≤ 0.05.
