# Supplementary material for: Xyloglucan processing machinery in Xanthomonas pathogens and its role in the transcriptional activation of virulence factors
Source: Nat Commun. 2021 Jun 30;12:4049. doi: 10.1038/s41467-021-24277-4 (PMC8245568; doi:10.1038/s41467-021-24277-4)
Supplement: Supplementary file 5 — Reporting Summary [file 41467_2021_24277_MOESM5_ESM.pdf]

nature research

Corresponding author(s): Mario Tyago Murakami and Priscila Oliveira de Giuseppe

Last updated by author(s): Jun 1, 2021

Reporting Summary

Nature Research wishes to improve the reproducibility of the work that we publish. This form provides structure for consistency and transparency in reporting. For further information on Nature Research policies, see our Editorial Policies and the Editorial Policy Checklist.

Statistics

For all statistical analyses, confirm that the following items are present in the figure legend, table legend, main text, or Methods section.

n/a	Confirmed	
☐	☒	The exact sample size (*n*) for each experimental group/condition, given as a discrete number and unit of measurement
☐	☒	A statement on whether measurements were taken from distinct samples or whether the same sample was measured repeatedly
☐	☒	The statistical test(s) used AND whether they are one- or two-sided<br>*Only common tests should be described solely by name; describe more complex techniques in the Methods section.*
☒	☐	A description of all covariates tested
☐	☒	A description of any assumptions or corrections, such as tests of normality and adjustment for multiple comparisons
☐	☒	A full description of the statistical parameters including central tendency (e.g. means) or other basic estimates (e.g. regression coefficient) AND variation (e.g. standard deviation) or associated estimates of uncertainty (e.g. confidence intervals)
☐	☒	For null hypothesis testing, the test statistic (e.g. *F*, *t*, *r*) with confidence intervals, effect sizes, degrees of freedom and *P* value noted<br>*Give P values as exact values whenever suitable.*
☒	☐	For Bayesian analysis, information on the choice of priors and Markov chain Monte Carlo settings
☒	☐	For hierarchical and complex designs, identification of the appropriate level for tests and full reporting of outcomes
☐	☒	Estimates of effect sizes (e.g. Cohen's *d*, Pearson's *r*), indicating how they were calculated

Our web collection on statistics for biologists contains articles on many of the points above.

Software and code

Policy information about availability of computer code

Data collection

Crystallographic data were collected using Pilatus 2M detector (Dectris) and MXCuBE 2 (Qt4) software at MX2-LNLS and using a Pilatus 6M detector (Dectris) and BluIce 4.0 software at BL9-2-SSRL. Dynamic light scattering data were collected using Zetasizer (7.12). SAXS data were collected using a CCD-Mar165 detector and fit2D v. 18 software. MS data were collected using MassLynx (4.1) (Waters) and LTQ XL TM (Thermo Fisher Scientific). Spectrophotometric data were collected using the i-Control software (1.10.4.0) (Tecan). SEC-MALS Data were processed using the ASTRA 6.0 software (Wyatt Technology).RNA integrity was evaluated in the Agilent 2100 Bioanalyzer (Agilent Technologies). For RNA sequencing, libraries were sequenced on HiSeq 2500 Illumina. RT-qPCR assays were performed in Applied Biosystems ViiA 7 RealTime equipment (Life Technologies). NMR data processing was performed using VnmrJ software (4.2 Revision A). Growth curves were performed in a SpectraMax M3 Multi-Mode Microplate Reader (Molecular Devices).

Data analysis

OriginPro (8.1) was used for non-linear curve fittings. Crystallographic data were indexed and scaled using XDS (version Jan 31st 2020 Built 20200417). Molecular replacement was performed using PHASER from Phenix package (dev-3139). Refinements were carried out with phenix.refine (dev-3139), using COOT (0.8.9) for manual building. Single-wavelenght anomalous diffraction data were processed using SHELXC/D/E from CCP4i package 7.0.023. Model validations were done using Molprobity (4.5) and Check My Metal server (https://cmm.minorlab.org/). Carbohydrate structures were evaluated using Privateer software from CCP4i2 package 1.0.2 revision 5710. Figures containing crystallographic coordinates were generated using Pymol (2.3 or 1.3). SAXS data were integrated using Fit2D (18) and processed using the ATSAS package (4.8.6) programs. The programs GNOM (5.0), DAMMIN (5.3), DAMAVER (5.0), CRYSOL (2.8.3) and SUPCOMB (2.3) were used for SAXS data processing. SAXSMoW server was used for molecular weight calculation (http://saxs.ifsc.usp.br/). Proteins oligomeric interface interaction energy was calculated by PDBePISA server (https://www.ebi.ac.uk/pdbe/pisa/). Paired-end reads (2x100pb) were filtered by quality and presence of adaptors using Trimmomatic v 0.38 and rRNA reads were filtered using SortmeRNA v. 2.0. QC reads were mapped into the Xanthomonas citri pv. citri str. 306 genome using Bowtie2 v.2.2.5 algorithm. Differential gene expression analysis was based on counting data and performed using the Bioconductor DESeq2 v.1.18.1 package using the R v.3.4.1 platform, by paired comparison against the control (XVM2m glucose) condition. Gene Ontology enrichment analysis was carried out using clusterProfiler v. 3.14.3 package. RT-qPCR statistical analysis were performed with Prism 8.4.1 (GraphPad). Gene expression stability was analysed in BestKeeper v.1 and NormFinder v.0.953 algorithms, and with the online tool RefFinder (https://www.heartcure.com.au/reffinder/?type=reference, accessed in November

2019). Quantitative analyses of canker lesions were performed using ImageJ v. 1.53b. Phylogenetic analysis was performed using the UBCG pipeline v. 3.0. Individual proteins were aligned using 7.299b. Phylogenetic analysis was inferred using RAxML 8.2.0, using the PROTGAMMAWAG model.

For manuscripts utilizing custom algorithms or software that are central to the research but not yet described in published literature, software must be made available to editors and reviewers. We strongly encourage code deposition in a community repository (e.g. GitHub). See the Nature Research guidelines for submitting code & software for further information.

Data

Policy information about availability of data

All manuscripts must include a data availability statement. This statement should provide the following information, where applicable:

- Accession codes, unique identifiers, or web links for publicly available datasets
- A list of figures that have associated raw data
- A description of any restrictions on data availability

Structural data have been deposited in the Protein Data Bank (https://www.rcsb.org/) under accession codes 7KN8 (XccXeg74A + XG) [https://doi.org/10.2210/pdb7KN8/pdb], 7KMM (XacXaeA) [https://doi.org/10.2210/pdb7KMM/pdb], 7KMN (XacGalD) [https://doi.org/10.2210/pdb7KMN/pdb], 7KMO (XacGalD + galactose) [https://doi.org/10.2210/pdb7KMO/pdb], 7KMP (XacXyl31A) [https://doi.org/10.2210/pdb7KMP/pdb], 7KNC (XacXyl31A + xylose) [https://doi.org/10.2210/pdb7KNC/pdb] and 7KMQ (XacAfc95A) [https://doi.org/10.2210/pdb7KMQ/pdb]. RNA-seq data were deposited in the Gene Expression Omnibus (GEO) database under accession number GSE159288 [https://www.ncbi.nlm.nih.gov/geo/query/acc.cgi?acc=GSE159288]. All other data generated or analyzed during this study are included in this published article (and its Supplementary information files and the Source Data) or are available from the corresponding authors upon reasonable request.

Other PDB identifiers used in this work
Aspergillus aculeatus CE12 rhamnogalacturonan acetylesterase 1DEO [10.2210/pdb1DEO/pdb]
Penicillium sp. GH35 beta-galactosidase 1TG7 [10.2210/pdb1TG7/pdb]
Arabidopsis thaliana CE6 acetyl xylan esterase 2APJ [10.2210/pdb2APJ/pdb]
Acetivibrio thermocellus GH74 beta-1,4-xyloglucan hydrolase/xyloglucanase 2CN2 [10.2210/pdb2CN2/pdb]
Bifidobacterium bifidum GH95 alpha-1,2-L-fucosidase 2EAB [10.2210/pdb2EAB/pdb]
Hungateiclostridium thermocellum CE3 acetyl xylan esterase 2VPT [10.2210/pdb2VPT/pdb]
Cellvibrio japonicus GH31 alpha-xylosidase 2XVG [10.2210/pdb2XVG/pdb]
Bacteroides vulgatus GH2 glycoside hydrolase 3GM8 [10.2210/pdb3GM8/pdb]
Trichoderma reesei GH35 beta-galactosidase/galacto-beta-galactanase 3OG2 [10.2210/pdb3OG2/pdb]
Homo sapiens GH35 beta-galactosidase 3THC [10.2210/pdb3THC/pdb]
Butyrivibrio proteoclasticus B316 CE2 acetylxylan esterase 3U37 [10.2210/pdb3U37/pdb]
Caulobacter vibrioides GH35 glycoside hydrolase 3U7V [10.2210/pdb3U7V/pdb]
Solanum lycopersicum GH35 beta-galactosidase/exo-beta-1,4-galactanase 3W5F [10.2210/pdb3W5F/pdb]
Cellvibrio japonicus GH35 beta-galactosidase 4D1I [10.2210/pdb4D1I/pdb]
Streptococcus pneumoniae GH35 beta-1,3-galactosidase 4E8D [10.2210/pdb4E8D/pdb]
Aspergillus oryzae GH35 beta-galactosidase 4IUG [10.2210/pdb4IUG/pdb]
Bacillus circulans GH35 beta-galactosidase 4MAD [10.2210/pdb4MAD/pdb]
Bacteroides ovatus GH95 alpha-L-galactosidase 4UFC [10.2210/pdb4UFC/pdb]
Bacillus circulans GH2 beta-galactosidase 4YPJ [10.2210/pdb4YPJ/pdb]
Pyrococcus horikoshii GH35 exo-beta-glucosaminidase 5GSL [10.2210/pdb5GSL/pdb]
Thermococcus kodakarensis GH35 exo-beta-glucosaminidase 5GSM [10.2210/pdb5GSM/pdb]
Cellvibrio japonicus GH35 beta-galactosidase 5JAW [10.2210/pdb5JAW/pdb]
Bacteroides uniformis str. 3978 GH2 beta-glucuronidase 6D8G [10.2210/pdb6D8G/pdb]
Bacteroides thetaiotaomicron GH35 glycoside hydrolase 6EON [10.2210/pdb6EON/pdb]
Roseburia intestinalis CE17 mannan esterase 6HFZ [10.2210/pdb6HFZ/pdb]
Pyrococcus furiosus GH35 glycoside hydrolase 6JOW [10.2210/pdb6JOW/pdb]
Ruminococcus gnavus GH2 beta-glucuronidase 6JZ1 [10.2210/pdb6JZ1/pdb]
Niastella koreensis GH74 endo-xyloglucanase 6P2L [10.2210/pdb6P2L/pdb]
Caldicellulosiruptor lactoaceticus GH74 endo-xyloglucanase 6P2M [10.2210/pdb6P2M/pdb]
Thermotoga maritima GH2 beta-galactosidase 6SD0 [10.2210/pdb6SD0/pdb]

Databases used in this study:
The Polysaccharide-Utilization Loci DataBase (PULDB) server (http://www.cazy.org/PULDB/)
Carbohydrate-Active enZYmes database (CAZY) (http://www.cazy.org/)
Reference Sequence (RefSeq) database (https://www.ncbi.nlm.nih.gov/refseq/)
Protein families database (Pfam) (http://pfam.xfam.org/)
Genbank protein database (https://www.ncbi.nlm.nih.gov/protein)
The Xanthomonas Resource (http://www.xanthomonas.org/t3e.html)

Field-specific reporting

Please select the one below that is the best fit for your research. If you are not sure, read the appropriate sections before making your selection.

☒ Life sciences ☐ Behavioural & social sciences ☐ Ecological, evolutionary & environmental sciences

For a reference copy of the document with all sections, see nature.com/documents/nr-reporting-summary-flat.pdf

Life sciences study design

All studies must disclose on these points even when the disclosure is negative.

Sample size	No sample size calculation was performed in advance or required statistical pre-determination. The number of enzymatic independent experiments was determined based upon previous studies with similar methodologies (doi.org/10.1038/s41589-020-0554-5), following the standard practice in enzymology (n=3). RNA-seq were performed over at least four biological independent experiments. RT-qPCR was performed with three biological experiments with at least two technical replicates. Canker measurements (pinprick inoculations) were performed with three independent experiments and 16 technical replicates. All other experiments were qualitatively assayed and no sample size calculation was needed prior to them.
Data exclusions	No data were excluded from the analyses.
Replication	All sample sizes are indicated in the respective figure legends. All quantitative enzyme assays consist of three independent experiments (n=3). RNA-sequencing, RT-qPCR and pinprick inoculations assays were repeated with at least three biological independent samples.
Randomization	Only objective measurements were performed in this study, which are mostly based on biochemical and biophysical assays, samples derived from transcriptional analysis, bacterial cultures or virulence assays, for which randomization is not relevant.
Blinding	This study reports objective measurements. In each experiment, the samples were treated identically. Therefore, blinding was not relevant.

Reporting for specific materials, systems and methods

We require information from authors about some types of materials, experimental systems and methods used in many studies. Here, indicate whether each material, system or method listed is relevant to your study. If you are not sure if a list item applies to your research, read the appropriate section before selecting a response.

Materials & experimental systems

n/a	Involved in the study
☒	☐ Antibodies
☒	☐ Eukaryotic cell lines
☒	☐ Palaeontology and archaeology
☒	☐ Animals and other organisms
☒	☐ Human research participants
☒	☐ Clinical data
☒	☐ Dual use research of concern

Methods

n/a	Involved in the study
☒	☐ ChIP-seq
☒	☐ Flow cytometry
☒	☐ MRI-based neuroimaging
